# Transplanted Donor- or Stem Cell-Derived Cone Photoreceptors Can Both Integrate and Undergo Material Transfer in an Environment-Dependent Manner

**DOI:** 10.1016/j.stemcr.2017.12.008

**Published:** 2018-01-04

**Authors:** Paul V. Waldron, Fabiana Di Marco, Kamil Kruczek, Joana Ribeiro, Anna B. Graca, Claire Hippert, Nozie D. Aghaizu, Aikaterini A. Kalargyrou, Amanda C. Barber, Giulia Grimaldi, Yanai Duran, Samuel J.I. Blackford, Magdalena Kloc, Debbie Goh, Eduardo Zabala Aldunate, Robert D. Sampson, James W.B. Bainbridge, Alexander J. Smith, Anai Gonzalez-Cordero, Jane C. Sowden, Robin R. Ali, Rachael A. Pearson

**Affiliations:** 1 UCL Institute of Ophthalmology, 11–43 Bath Street, London EC1V 9EL, UK; 2 Stem Cells and Regenerative Medicine Section, UCL Great Ormond Street Institute of Child Health, University College London, 30 Guilford Street, London WC1N 1EH, UK

**Keywords:** photoreceptor, retina, blindness, retinal dystrophy, Nrl^−/−^, function, transplantation, material transfer, fusion, integration

## Abstract

Human vision relies heavily upon cone photoreceptors, and their loss results in permanent visual impairment. Transplantation of healthy photoreceptors can restore visual function in models of inherited blindness, a process previously understood to arise by donor cell integration within the host retina. However, we and others recently demonstrated that donor rod photoreceptors engage in material transfer with host photoreceptors, leading to the host cells acquiring proteins otherwise expressed only by donor cells. We sought to determine whether stem cell- and donor-derived cones undergo integration and/or material transfer. We find that material transfer accounts for a significant proportion of rescued cells following cone transplantation into non-degenerative hosts. Strikingly, however, substantial numbers of cones integrated into the *Nrl*^*−/−*^ and *Prph2*^*rd2/rd2*^, but not *Nrl*^*−/−*^;*RPE65*^*R91W/R91W*^, murine models of retinal degeneration. This confirms the occurrence of photoreceptor integration in certain models of retinal degeneration and demonstrates the importance of the host environment in determining transplantation outcome.

## Highlights

• Transplanted cone photoreceptors can incorporate into host retina
• Transplanted cone photoreceptors undergo material transfer with host photoreceptors
• Host retinas with disrupted cytoarchitecture facilitate cone incorporation
• Host environment affects relative contributions of integration and material transfer

## Graphical Abstract

**Figure:**
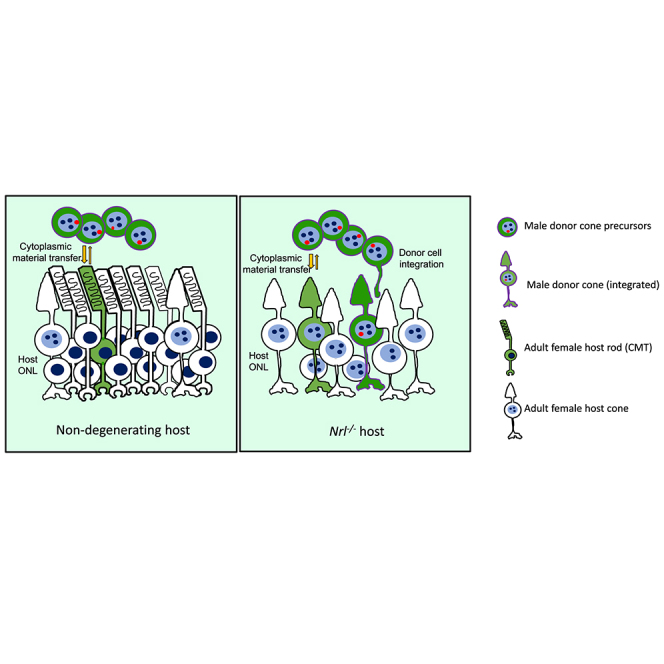

## Teaser

Pearson and colleagues demonstrate that transplanted cone photoreceptors can both undergo incorporation into the host neural retina and engage in cytoplasmic material transfer with host rod and cone photoreceptors. They show that the host environment plays a crucial role in determining the relative contributions of these two mechanisms to transplantation outcome.

## Introduction

Loss of vision due to photoreceptor degeneration is a leading cause of blindness in the developed world, and replacing lost photoreceptors by the transplantation of healthy cells represents a promising therapeutic strategy. We, and others, have previously reported the effective transplantation of post-mitotic rod precursors either isolated from developing retinas or derived from murine or human pluripotent stem cells (PSCs) (MacLaren et al., 2006, Bartsch et al., 2008, Lakowski et al., 2010, Pearson et al., 2012, Gonzalez-Cordero et al., 2013). When transplanted into murine models of retinal disease, and if present in sufficiently large numbers, these cells have been shown to improve various measures of visual function (Barnea-Cramer et al., 2016, Singh et al., 2013, Barber et al., 2013, MacLaren et al., 2006, Pearson et al., 2012). Together, these findings demonstrate that transplanted donor rod photoreceptor cells have the potential to restore vision.

Human vision relies heavily on cone photoreceptors, and diseases that lead to their loss, such as age-related macular degeneration (AMD), are particularly devastating. We previously provided the first report of cone transplantation (Lakowski et al., 2010) using a *Crx*-GFP transgenic line that labels rod and cone photoreceptors. We transplanted embryonic *Crx*-GFP^+^ donors at a stage when the majority was committed to a cone fate. While large numbers of GFP-labeled photoreceptors were found in the host outer nuclear layer (ONL), many resembled rods in their morphology. The mixed nature of the *Crx*-GFP^+^ donor population presented the question of whether the preponderance of rod-like cells was due to plasticity in the fate of the donor photoreceptors (Siegert et al., 2012) or the result of more successful integration of the rod precursors present within the mixed population.

NRL and NR2E3 act together with CRX to activate rod-specific genes and suppress cone gene expression. Rod differentiation is thus impaired in *Nrl* and *Nr2e3* deficient retinas; the *Nr2e3*^*rd7/rd7*^ mouse has increased numbers of S-opsin^+^ cone-like photoreceptors, while in the *Nrl*^*−/−*^ mouse, all photoreceptors fated to become rods instead acquire a cone-like (so-called “cod”) hybrid phenotype. In keeping with the idea that photoreceptors might retain plasticity after terminal mitosis, Ader and colleagues (Santos-Ferreira et al., 2015) noted that following transplantation of postnatally derived *Nrl*^*−/−*^GFP^+^ cone-like cells, GFP^+^ cells within the retina of *Pde6c*^*cpfl1/cpfl1*^ model of cone degeneration bore rod-like morphological features, including small spherule synapses and elongated outer segments. Strikingly, though, these cells also expressed cone arrestin (CARR) and S-OPSIN and appeared capable of driving responses to photopic stimuli (Santos-Ferreira et al., 2015). Another recent study by Wallace and colleagues (Smiley et al., 2016) described the transplantation of *Nrl*^*−/−*^ cells and those derived from a novel cone-GFP reporter mouse line (*Ccdc136*-GFP). Similarly, apparently integrated donor cells exhibited morphologies more typical of rods than cones, but in this study cone marker expression was not observed.

During our own investigations into photoreceptor transplantation, we made observations that led us to question the underlying cellular mechanisms behind functional rescue following donor photoreceptor transplantation (Pearson et al., 2016). While donor rod photoreceptor migration and integration occurs, it accounts for far fewer of the reporter-labeled cells observed than previously thought; post-mitotic rod precursors can also undergo a process of material transfer with photoreceptors within the recipient retina (Pearson et al., 2016) (see also Ortin-Martinez et al., 2017, Singh et al., 2016, Decembrini et al., 2017, Santos-Ferreira et al., 2016). The cellular mechanisms by which this occurs have yet to be determined but they do not appear to involve permanent donor-host nuclear or cell fusion, or the uptake of free protein or nucleic acid from the extracellular environment. Instead, it appears that a wide array of either RNAs and/or proteins might be exchanged between stage-specific donor rod precursors and adult host photoreceptors *in vivo* (Pearson et al., 2016), apparently in quantities sufficient to render the recipient cells functional.

Here, we sought to determine whether purified cone photoreceptors, derived either from donor retinas or from embryonic stem cell (ESC)-derived retinas, undergo cell integration and/or engage in material transfer with host photoreceptors after transplantation into different models of retinal degeneration. Specifically, we sought to determine whether the host environment influenced the relative contributions of these two mechanisms to transplantation outcome.

## Results

### Transplantation of Donor- and Stem Cell-Derived Cone Precursors into Wild-Type Recipient Results in GFP^+^ Cells within Host ONL with Rod-like Morphologies

We first assessed the outcomes of transplantation of cone photoreceptors isolated from a variety of donor- and stem cell-derived sources. To transplant purified populations of cone precursors at different stages of development, we used the *Chrnb4*-EGFP (Figures S1A and S2A) and *OPN1LW*-EGFP cone reporter mouse lines to isolate early- and late-stage cone precursors, respectively. *L/MOpsin*-GFP reporter virally labeled cones were additionally derived from murine ESC (mESC) retinal organoid cultures, as we have described previously (Kruczek et al., 2017, Gonzalez-Cordero et al., 2013). Previously we, and others, have found rod photoreceptor transplantation outcome to be significantly affected by the developmental stage of the donor cell at the time of transplantation. Therefore, GFP^+^ cone precursors were isolated at various stages of development: embryonic day 15 (E15) and post-natal day 1 (P1) (peaks of cone and rod birth [Young, 1985]), and P8 (stage most effective for rod transplantation [Pearson et al., 2012]).

**Figure 1:**
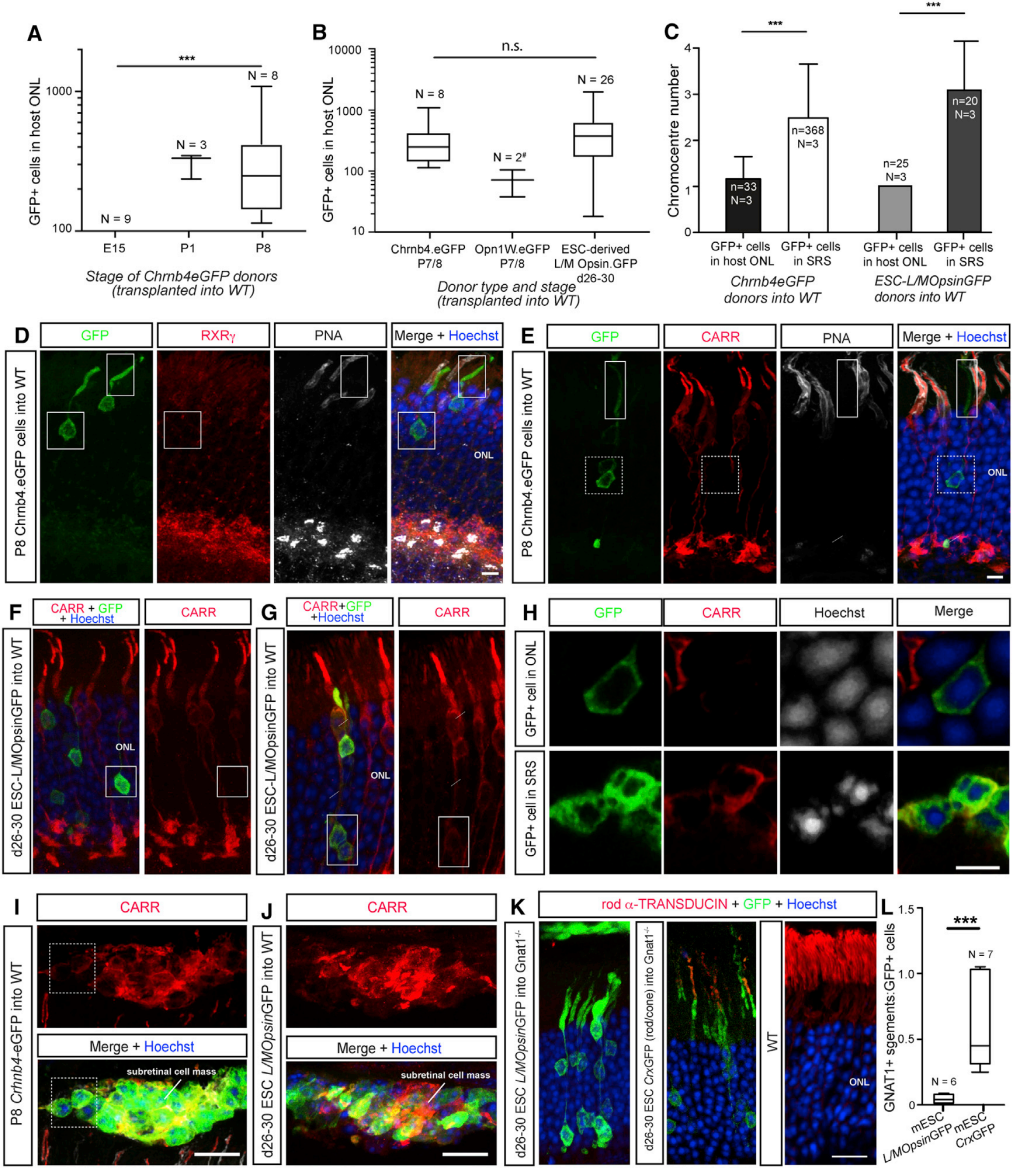
Transplantation of Donor- and Stem Cell-Derived Cone Precursors Leads to the Presence of Rod-like Cells within the Host ONL (A and B) Box-and-whiskers (SD) plot of number of GFP^+^ cells within wild-type (WT) host ONL after transplantation of (A) *Chrnb4*-EGFP^+^ cells from different stages of development and (B) donor-derived *OPN1LW*-EGFP^+^ and mESC-derived *L/MOpsin-*GFP^+^ cone precursors, compared with donor-derived *Chrnb4*-EGFP^+^ cells at an equivalent stage of development (∼P7/8). (C) Histogram of mean number of chromocenters/nucleus (mean + SD) of GFP^+^ cells within the host ONL and in the subretinal space (SRS) following transplantation of P7/8 *Chrnb4-*EGFP^+^ and day 26–30 mESC-derived *L/MOpsin*-GFP^+^ donors. (D–G) IHC shows that, regardless of donor origin, most GFP^+^ cells within host ONL are rod-like in morphology and display a heterogeneous expression profile with respect to cone markers (compare F and G). (H–J) GFP^+^ cells within the host ONL typically have single chromocenter nuclei (H), while GFP^+^ donor cells in the SRS have nuclei with multiple chromocenters and (I) and (J) express cone markers. (K) Confocal images showing rod α-transducin staining in *Gnat1*^*−/−*^ host retina after transplantation of mESC-derived *L/MOpsin*-GFP (cone) or mESC-derived *Crx-*GFP (rod and cone) donor cells. (L) Box-and-whiskers (SD) plot showing quantification of the number of ONL-located GFP^+^ cells expressing rod α-transducin. ANOVA with correction for multiple comparisons. Cells in (H) show regions of interest depicted in (E) and (I), respectively. ^∗∗∗^p < 0.001 (# indicates statistical tests not applied to *OPN1LW*-EGFP dataset due to low N). N, number of eyes; n, number of cells. n.s., not significant; CARR, cone arrestin; PNA, peanut agglutinin. Scale bars, 10 μm.

In the developing *Chrnb4*-EGFP retina, GFP expression was heterogeneous but a population of brightly fluorescent GFP^+^ cells ([GFP]_high_) could be readily isolated at P1, P8, and adult stages by fluorescence-activated cell sorting (FACS) that comprised cone precursors (Figures S1A, S1D, S1E, S2A, and S2B; Tables S1 and S2). At E15, it was not possible to isolate sufficient numbers of [GFP]_high_ cells so, for this age, all GFP^+^ cells were collected. In each case, purified GFP^+^ cells were transplanted into adult wild-type recipients and assessed 2–3 weeks after transplantation. In contrast to rod transplantation (Pearson et al., 2012), we observed a high transplantation failure rate (N = 9 successful transplants/20 total transplanted eyes). Donor cell masses, usually an indicator of successful transplantation, were frequently absent from the subretinal space (SRS), yet there was little evidence of acute rejection (see Warre-Cornish et al., 2014, West et al., 2010). In those transplants meeting the criteria (see Supplemental Experimental Procedures), a small number of GFP^+^ cells were seen in the host ONL. Similar numbers were seen using P1 (n = 306 ± 34; N = 3/6) and P8 (352 ± 112; N = 8/14) donors (Figure 1A). This is lower than that reported previously following the transplantation of *Nrl*-GFP^+^ rod photoreceptors, where thousands of GFP^+^ cells can be found in the wild-type host ONL (Warre-Cornish et al., 2014, Pearson et al., 2012). Transplanted populations of purified *OPN1LW*-EGFP^+^ donor cells yielded similarly small numbers of GFP^+^ cells within the recipient ONL (72 ± 47 cells; N = 2/6; Figure 1B), although loss of the line prevented further investigation.

To date, reports of cone transplantation have focused on donor-derived cells. We therefore sought to examine the behavior of stem cell-derived cones. mESC-derived retinal organoids were differentiated and transduced with a viral vector (ShH10.*L/MOpsin*.GFP) to label L/M cones, which could be purified by FACS, as previously described (Kruczek et al., 2017, Gonzalez-Cordero et al., 2013). *L/MOpsin-*GFP^+^ cells were taken from days 26–30 of differentiation, equivalent to ∼ P6–P8 (Kruczek et al., 2017), and transplanted into adult wild-type recipients. Similar numbers of GFP^+^ cells were found in the host wild-type ONL (466 ± 86 GFP^+^ cells; N = 26/26) (Figure 1B) as those seen following transplants of *Chrnb4*-EGFP^+^ or *OPN1LW*-EGFP^+^ donor-derived cones.

Transplantation of mESC- and donor-derived cones into the SRS both yielded the presence of GFP^+^ cells within the wild-type host ONL. However, these cells presented with morphologies more typical of rods, including some or all of: rounded cell bodies distributed throughout the ONL, rather than at the apical margin like mature cones (Figures 1D–1G and S2C–S2E); round spherule-like synapses; long segments; and highly condensed nuclei with a single chromocenter (Figures 1C, 1H, S2C, and S2D). Immunohistochemistry (IHC) showed that these cells typically did not express cone markers (Figures 1D–1F), although some examples were seen (e.g., Figure 1G). Conversely, the mass of injected donor cells that remained in the SRS had cone-like nuclei with multiple chromocenters (Figures 1C, 1H, and S2E) and many expressed the cone marker CARR (Figures 1H and 1I), consistent with the expression profile of the donor cells *in vivo* (Figure S1A) and by mESC-derived L/MOpsinGFP^+^ cells *in vitro* (Kruczek et al., 2017).

Given that cone precursors continue to express robust levels of rod-specific genes for many days after terminal mitosis (Table S2), we considered the possibility that the rod-like GFP^+^ cells located within the host ONL might co-express rod markers and represent a hybrid state. Co-staining for rod markers was attempted, but the very high levels of expression by neighboring wild-type host rods prevented us from making assessments of co-localization with any certainty. We therefore transplanted d26-29 *L/MOpsin*GFP^+^ mESC-derived cone precursors into the *Gnat1*^*−/−*^ (rod α-transducin knockout) mouse model, in which rods are non-functional but do not degenerate. Despite their rod-like appearance and condensed nuclei (Figure S2F), rod α-TRANSDUCIN expression was typically absent after transplantation of mESC-derived *L/MOpsin*-GFP^+^ cones (Figures 1K and 1L). The rare rod α-transducin^+^, GFP^+^ events seen most likely reflect inclusion of occasional rods in the transplanted donor population after FACS. Conversely, rod α-TRANSDUCIN was co-expressed by most GFP^+^ cells within the host ONL following transplantation of *Crx*-GFP^+^ ESC-derived (predominantly rod) photoreceptors (Figures 1K and 1L), as reported previously for *Nrl*-GFP^+^ (rod) donor-derived photoreceptors (Pearson et al., 2016).

### Transplantation of Cone-like Photoreceptor Precursors into Wild-Type Recipients

Given the preponderance of rod-like morphologies seen following transplantation of purified cones, we sought to genetically restrict the donor cell population's potential by deletion of key rod differentiation genes (*Nrl* and *Nr2e3*). By crossing *Nrl*^*−/−*^ mice with *Nrl-*GFP mice, all cone-like cells express GFP, albeit at a lower level than in *Nrl*-GFP (Figures S1B, S1D, and S1E). In the *Nr2e3*^*rd7/rd7*^ retina, early-born immature rods switch fate to become true S cones and late-born rods become “cone-like.” By crossing *Nr2e3*^*rd7/rd7*^ mice with *Crx-*GFP mice, both true cones and cone-like cells carry the GFP label. Interestingly, IHC and qRT-PCR for cone markers demonstrated a hybrid status of the genetically engineered GFP^+^ photoreceptors from these two crosses (Figure S1; Tables S3 and S4). For example, RXRγ was widespread in the vast majority of GFP^+^ cells in both lines, but CARR and THRβ2 were not markedly higher than in wild-type mice.

**Figure 2:**
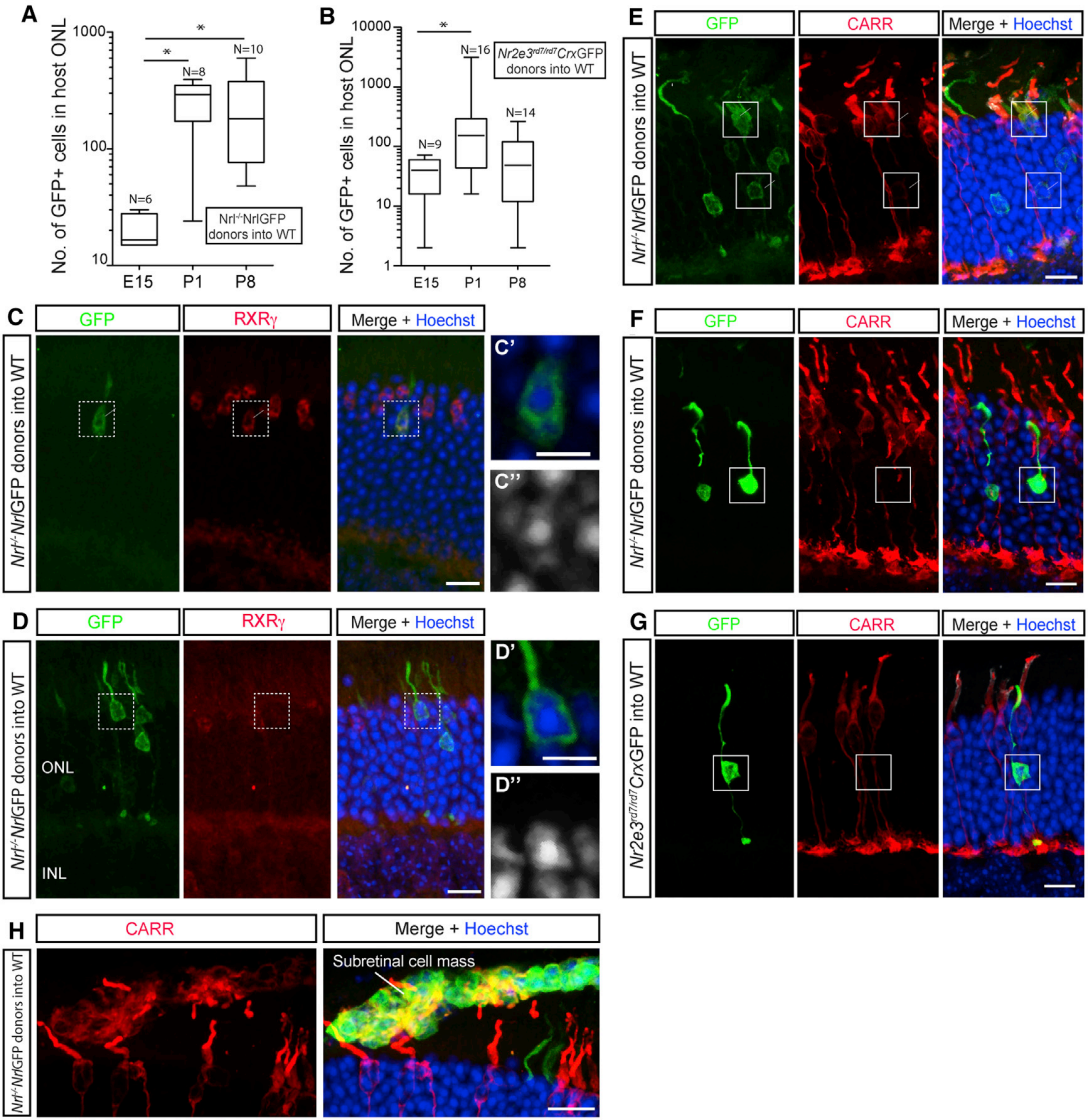
Transplantation of Genetically Engineered Cone-like Cells Leads to the Presence of Rod-like Cells within the Host ONL (A and B) Box-and-whiskers (SD) plot showing the effect of donor cells age on the number of GFP^+^ cells found within wild-type host retina after transplantation of (A) *Nrl*^*−/−*^;*Nrl-*GFP or (B) *Nr2e3*^*rd7/rd7*^;*Crx-GFP*. ANOVA with correction for multiple comparisons; ^∗^p < 0.05. N, number of eyes. (C–H) Confocal images showing heterogeneous expression profile with respect to cone markers including (C) RXRγ^+^ and (D) RXRγ^*−*^ cells, and (E) CARR^+^ and (F–H) CARR^*−*^ cells in wild-type host ONL after transplantation of *Nrl*^*−/−*^;*Nrl*-GFP donors (C–F) and *Nr2e3*^*rd7/rd7*^;*Crx*-GFP donors (G). Cells in (C′, C″) and (D′, D″) show regions of interest depicted in (C) and (D), respectively. Scale bars, 10 μm.

Transplantation of either *Nrl*^*−/−*^;*Nrl*-GFP^+^ (Figures 2A and 2C–2F) or *Nr2e3*^*rd7/rd7*^;*Crx*-GFP^+^ (Figures 2B and 2G) resulted in the presence of GFP^+^ cells in the wild-type adult host ONL. Significantly higher numbers of GFP^+^ cells were seen using post-natal, compared with embryonic (Figures 2A and 2B), donors as reported previously for rod and cone/rod populations (MacLaren et al., 2006, Lakowski et al., 2010, Gonzalez-Cordero et al., 2013) and the pure cone populations described above. There was no significant difference in outcome between the two different types of donor cell, but both yielded much lower numbers of ONL-located GFP^+^ cells than seen previously using the respective rod-only (*Nrl-*GFP) (Pearson et al., 2012) and cone/rod (*Crx*-GFP) (Lakowski et al., 2010) donor cell controls. We used IHC to examine the identity of the GFP^+^ cells. Again, most GFP^+^ cells within the host ONL had a morphological appearance consistent with rod photoreceptors (Figures 2C–2G). Often, they had condensed rod-like nuclei (Figures 2C′, 2C″, and S2F) and typically did not express RXRγ (Figure 2E). Rare examples of cone-like cells were seen (<10 cells per eye); these additionally had nuclei more typical of cones and were RXRγ^+^ (Figure 2D). Other cone photoreceptor markers were expressed in a heterogeneous manner with some (Figure 2E), but not all (Figures 2F and 2G), expressing cone markers. In keeping with the heterogeneous expression of cone markers in the donor mouse lines (Figures S1B and S1C), GFP^+^ donor cells from *Nrl*^*−/−*^;*Nrl*-GFP and *Nr2e3*^*rd7/rd7*^;*Crx*-GFP donors that remained within the SRS displayed a mixed expression profile, with some but not all expressing CARR (e.g., Figure 2H).

Together, these data confirm that regardless of origin, mESC- and donor-derived cones, as well as photoreceptors genetically restricted from becoming rods, behave in a similar manner following their transplantation into the intact wild-type retina: transplantation resulted in the presence of small numbers of predominantly rod-like GFP^+^ cells within the host ONL.

### Transplanted Cone and Cone-like Photoreceptors Engage in Material Transfer with Host Photoreceptors in the Intact Wild-Type Retina

**Figure 3:**
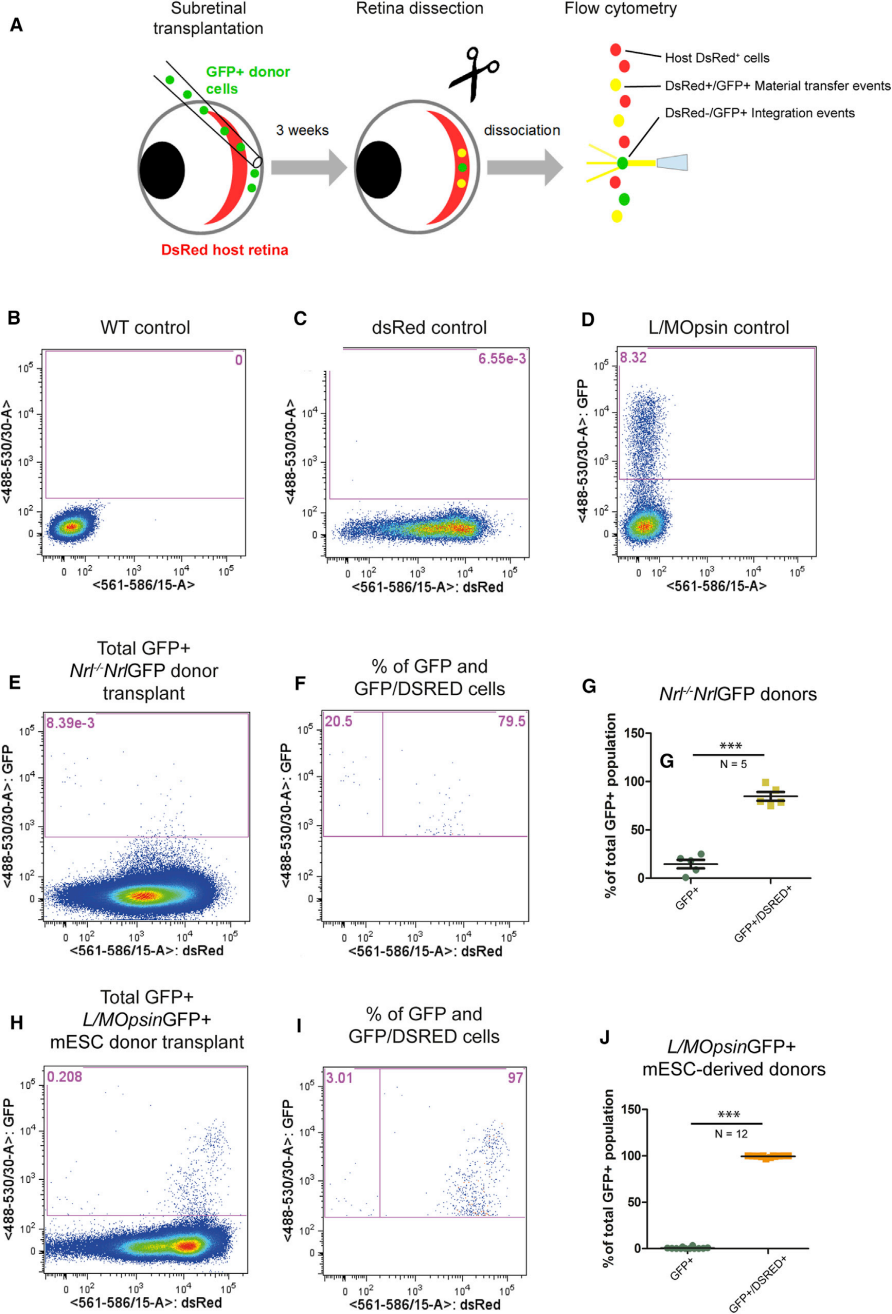
Transplanted Cone Photoreceptors Undergo Material Transfer with Wild-Type Host Photoreceptors *Nrl*^*−/−*^;*Nrl-GFP* or mESC-derived *L/MOpsin*-GFP post-mitotic photoreceptor precursor donor cells were transplanted into *dsRed* hosts and examined by flow cytometry 2–3 weeks post transplantation. (A) Schematic of the experimental protocol. (B–D) Representative flow-cytometry plots for adult (B) wild-type (negative control), (C) *dsRed* (positive control), and (D) *L/MOpsin-GFP* (positive control) retinas. Pink box shows gating for GFP^+^ cells. (E and F) Representative plots from an example of a host retina transplanted with *Nrl*^*−/−*^;*Nrl*-GFP donor cells showing (E) percentage of total retinal cells that were GFP^+^ (pink box) and (F) the proportion of these that were GFP^+^ only (left pink box) or GFP^+^/dsRed^+^ (right pink box). (G) Box-and-whiskers plot showing median and range for percentage of GFP^+^ only and GFP^+^/dsRed^+^ photoreceptors within each host retina after transplantation of *Nrl*^*−/−*^;*Nrl*-GFP donor cells. (H–J) representative plots (H and I) and box-and-whiskers (SD) plot (J) from retinas transplanted with mESC-derived *L/MOpsin*-GFP donor cells. ^∗∗∗^p < 0.001, unpaired t test. N, number of eyes.

We (Pearson et al., 2016), and others (Santos-Ferreira et al., 2016, Singh et al., 2016), recently reported that transplanted donor-derived rod photoreceptor precursors engage in material transfer with photoreceptors in the intact host retina. Given that most of the GFP^+^ cells within the wild-type recipients bore a striking morphological resemblance to rod photoreceptors, we sought to determine whether these arose from a process of material transfer. Donor-derived P8 *Nrl*^*−/−*^;*Nrl-*GFP^+^ cells or mESC-derived d26-29 *L/MOpsin*-GFP^+^ cells were transplanted into *dsRed*^+/*−*^ recipients, which have normal retinas but with all the cells ubiquitously expressing the fluorescent reporter, dsRed. At 2–3 weeks post transplantation, host retinas were carefully dissected free from any remaining SRS cell mass, dissociated, and analyzed using flow cytometry (Figure 3). As we reported previously for rods (Pearson et al., 2016), the vast majority of the apparently integrated GFP^+^ cells co-expressed dsRed (85% ± 10% SD, N = 5 following transplantation of *Nrl*^*−/−*^;*Nrl-*GFP cells and 99% ± 1%, N = 12 following transplantation of *L/MOpsin-*GFP mESC cones; see note in Supplemental Experimental Procedures). This suggests that transplanted cones and cone-like cells can undergo material transfer with rod photoreceptors in the intact wild-type host retina in a manner resembling that recently described for rod-to-rod transfer (Pearson et al., 2016, Santos-Ferreira et al., 2016, Singh et al., 2016), albeit with apparently poorer efficiency.

### Transplantation of Nrl^−/*−*^ Cone-like Photoreceptor Precursors into Different Retinal Environments

Material transfer appears to account for a significant proportion of the GFP^+^ cells found within the intact wild-type host ONL after transplantation of rod (Singh et al., 2016, Santos-Ferreira et al., 2016, Pearson et al., 2016) and cone (this paper and Ortin-Martinez et al., 2017, Decembrini et al., 2017) photoreceptors. However, we have previously demonstrated the real-time integration of rod photoreceptors into the disrupted retina of the *Prph2*^*rd2/rd2*^ (*retinal degeneration slow*, *rds*) mouse (Pearson et al., 2016) and that some degenerating retinas support better transplantation outcomes (number of GFP^+^ cells in the host ONL) than others (Barber et al., 2013).

We sought to examine what impact the recipient retinal environment has on transplantation outcome and the relative contributions of material transfer and/or integration by transplanting P8 *Nrl*^*−/−*^;*Nrl*-GFP^+^ cone-like cells into models of cone dysfunction and degeneration and into cone-enriched, rod-depleted environments. The first group included *Cnga3*^*cpfl5/cpfl5*^, which have mislocalized, non-functional cones that degenerate slowly over several weeks; *Pde6c*^*cpfl1/cpfl1*^, which have non-functional, rapidly degenerating cones; and *Prph2*^*rd2/rd2*^, in which all photoreceptors fail to produce outer segments and undergo degeneration (rods, then cones) over a period of several months. The second group included the largely non-degenerative but cone-enriched *Nr2e3*^*rd7/rd7*^ and *Nrl*^*−/−*^ models.

**Figure 4:**
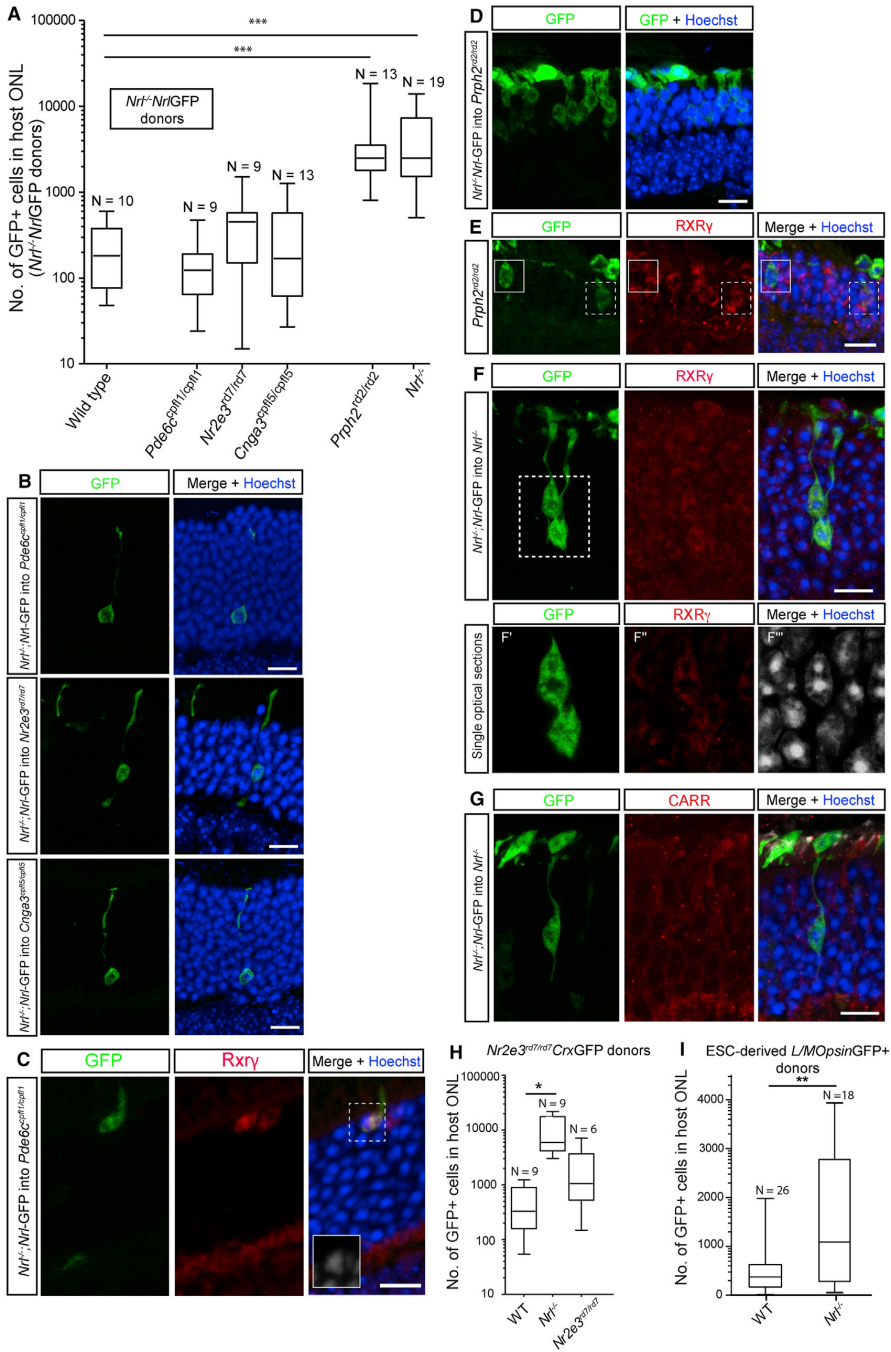
Transplantation Outcome Is Dependent on the Host Environment (A) Box-and-whiskers (SD) plot showing the influence of host environment on the number of GFP^+^ cells in the host ONL after transplantation of genetically engineered cone-like *Nrl*^*−/−*^;*Nrl*-GFP cells. Many more cells were found in the *Nrl*^*−/−*^ and *Prph2*^*rd2/rd2*^ hosts than any other model. (B–G) Confocal images showing representative examples of GFP^+^ cells in the different host retinas. In (E) and (F) solid-line box depicts GFP^+^/RXRγ^*−*^ cells and dashed-line box depicts GFP^+^/RXRγ^+^ cells. (H and I) Box-and-whiskers plots showing that the *Nrl*^*−/−*^ host environment supports similarly increased numbers of GFP^+^ cells after transplantation of (H) *Nr2e3*^*rd7/rd7*^;*Crx*-GFP photoreceptors and (I) mESC-derived *L/MOpsin*-GFP^+^ cones. ANOVA with correction for multiple comparisons: ^∗∗∗^p < 0.001, ^∗∗^p < 0.01, ^∗^p < 0.05. N, number of eyes. Scale bars, 10 μm.

At 2–3 weeks post transplantation, similar numbers of GFP^+^ cells were found in the ONL of *Cnga3*^*cpfl5/cpfl5*^ (354 ± 108, N = 13/17), *Pde6c*^*cpfl1/cpfl1*^ (149 ± 45, N = 11/13), and *Nr2e3*^*rd7/rd7*^ (476 ± 147, N = 9/11) hosts as found in wild-type hosts (242 ± 62, N = 10/15) (Figure 4A). They also presented morphological profiles resembling those seen in wild-type retinas; both rod-like (Figure 4B) and cone-like (Figure 4C) morphologies were observed, with most resembling rods in their morphology, location, and IHC profile. Rarely, GFP^+^/RXRγ^+^ cells with multichromocenter nuclei were seen correctly located at the apical margin (Figure 4C). No obvious increases in the incidence of cone-like GFP^+^ cells were seen in the *Pde6c*^*cpfl1/cpfl1*^ and *Cnga3*^*cpfl5/cpfl5*^ retinas.

In contrast, markedly higher numbers of GFP^+^ cells (3,780 ± 1,265, N = 13/15; p < 0.001) were seen in the *Prph2*^*rd2/rd2*^ recipient, compared with wild-type (Figures 4A and 4D). These cells showed a variety of morphologies and IHC profiles, with qualitatively more located at the apical margin and expressing RXRγ (Figure 4E, dotted-line box), although others did not (Figure 4E, solid-line box). Similarly, the number of GFP^+^ cells within the *Nrl*^*−/−*^ host ONL was significantly higher (4,631 ± 971, N = 19/24; p < 0.001) (Figure 4A) than that found in wild-type or any of the other models of cone degeneration, as also recently reported by others (Santos-Ferreira et al., 2015, Smiley et al., 2016). Imaging revealed that GFP^+^ cells within the *Nrl*^*−/−*^ host presented a cone-like phenotype with enlarged multichromocenter nuclei, typical of both normal cones and the host cone-like cells (Figures 4F and S2G), and expressed RXRγ (Figure 4F), while cone arrestin was more heterogeneous (Figure 4G). Transplantation of P8 *Nr2e3*^*rd7/rd7*^*Crx*-GFP^+^ cells (Figure 4H) and day 26–29 *L/MOpsin*-GFP^+^ mESC-derived cells (Figure 4I) similarly resulted in significantly higher numbers of GFP^+^ cells within the *Nrl*^*−/−*^ host ONL (9,690 ± 2,442, N = 9/9, p < 0.01 unpaired t test; and 1,415 ± 311, N = 18/18, p < 0.01 unpaired t test, respectively) compared with wild-type hosts (503 ± 144, N = 9/18; 466 ± 86, N = 26/26, respectively), indicating that the increase was a consequence of the host environment. Other recipient models were not tested with these donor cells.

### Increased Donor Cell Integration Partially Accounts for Increased Numbers of GFP^+^ Cells in Cone-Only Nrl^−/−^ Host Retina

**Figure 5:**
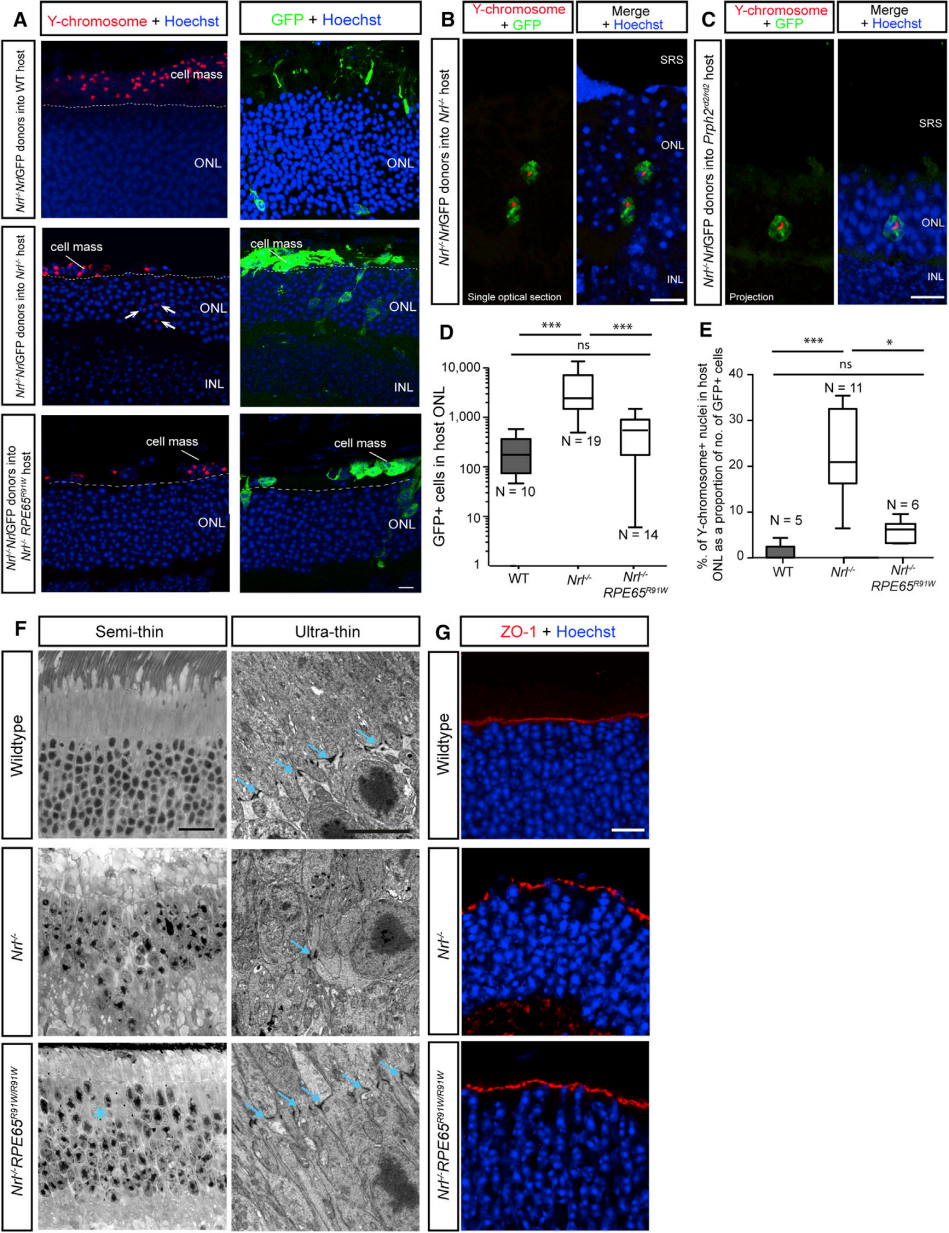
Transplanted Cone Photoreceptors Integrate in Some Models of Retinal Degeneration (A) Confocal images of FISH and GFP labeling in consecutive serial sections (exception being wild-type where GFP is from a different region), showing significant numbers of Y chromosome^+^ (red) nuclei in *Nrl*^*−/−*^ host ONL (arrows) but few, if any, in wild-type or *Nrl*^*−/−*^;*RPE65*^*R91W/R91W*^ host ONL, after transplantation of P7/8 *Nrl*^*−/−*^;*Nrl*-GFP donors. Cell masses located in SRS also demonstrate widespread labeling. (B and C) examples of retinal sections co-stained for both GFP and Y chromosome following transplantation of *Nrl*^*−/−*^;*Nrl*-GFP into (B) *Nrl*^*−/−*^ and (C) *Prph2*^*rd2/rd2*^ hosts. (D) Box-and-whiskers (SD) plot of total number of GFP^+^ cells in the Nrl^−/−^ host ONL, compared with *Nrl*^*−/−*^;*RPE65*^*R91W/R91W*^ and wild-type. ANOVA with correction for multiple comparisons: ^∗∗∗^p < 0.001; ns, not significant. (E–G) Box-and-whiskers (SD) plot (E) of total number of Y chromosome^+^ nuclei, as a proportion of total number of GFP^+^ cells, located within the host ONL in wild-type, *Nrl*^*−/−*^, and *Nrl*^*−/−*^;*RPE65*^*R91W/R91W*^ recipients. Kruskal-Wallis test: ^∗∗∗^p < 0.001; ^∗^p < 0.05; ns, not significant. N, number of eyes. Ultrastructural analysis (F) and IHC (G) for the OLM marker, ZO-1 (red) and the gliosis marker, Gfap (green) show disruption in OLM integrity in the *Nrl*^*−/−*^, but not the *Nrl*^*−/−*^;*RPE65*^*R91W/R91W*^ or wild-type, retina. Blue arrows denote adherens junctions; blue asterisk denotes region of ONL disruption. Scale bars, 10 μm (A–C, G), 25 μm (F, semi-thin), and 5 μm (F, ultrathin).

While material transfer likely explains the presence of rod-like cells in the wild-type retina following transplantation of cones, it does not necessarily explain the significantly higher numbers of GFP^+^ cells in disease models, such as the *Nrl*^*−/−*^ and *Prph*^*rd2/rd2*^ recipients. We therefore sought to directly investigate the level of donor cell integration into the recipient ONL by performing fluorescence *in situ* hybridization (FISH) for the Y chromosome (Y^+^) following the transplantation of male donor cells (Figure 5). Male *Nrl*^*−/−*^;*Nrl*-GFP^+^ donors were transplanted into either female *Nrl*^*−/−*^ or wild-type hosts. Few, if any, cells within the wild-type ONL bore the Y chromosome (Figure 5A, top). However, we observed many Y^+^ nuclei that were incontrovertibly located within the ONL of host *Nrl*^*−/−*^ retinas (Figures 5A [middle] and S3A), demonstrating that robust donor cell integration can occur into this model. Technical limitations meant that it was difficult to routinely obtain robust labeling for both GFP and the Y chromosome probe in the same retinal section, but examples are shown in Figure 5B. To quantify the proportion of events ascribable to integration, as opposed to material transfer, in this model we counted the number of GFP^+^ cells within the ONL and performed FISH on different, but consecutive, sections in the same retina. Y^+^ nuclei were found at a ratio of ∼1:5, with respect to the number of GFP^+^ cells in consecutive sections in the same eyes, compared with only ∼1:100 in wild-type hosts. When expressed as a percentage of the total GFP^+^ cells/eye, Y^+^ nuclei accounted for 23% (±3%; N = 11) of GFP^+^ cells in the same *Nrl*^−/−^ eyes, compared with 1% (±0.8%; N = 5) in the wild-type host (Figure 5B). This indicates that around one-fifth of the GFP^+^ cells seen in the *Nrl*^−/−^ host arise from donor cell integration. Similarly, we observed an increased number of Y^+^ nuclei in the *Prph2*^*rd2/rd2*^ model (14% ± 3% of total GFP^+^ cells; N = 6). We considered whether there were consistent differences in morphologies potentially arising from integrated cells versus those arising from material transfer, since integrated cells might fail to fully develop. Unfortunately, much of the finer details of the GFP signal, such as the apical and basal processes, are lost when combining with Y-probe staining in the same section (see Figure 5B). This makes it difficult to draw firm conclusions, but at a gross level the Y^+^, GFP^+^ cell bodies appear quite normal.

In contrast to a very recent report (Ortin-Martinez et al., 2017), these data strongly suggest that integration of cone photoreceptors can occur alongside material transfer, at least in certain host environments. This raises the question of why the *Nrl*^*−/−*^ retinal environment supports integration while the intact wild-type retina does not. The *Nrl*^*−/−*^ retina is notably disturbed in its cytoarchitecture: whorls and rosettes are common and the outer limiting membrane (OLM) is also disturbed in this model (Stuck et al., 2012). Since we have previously demonstrated that OLM integrity influences transplantation outcome (Pearson et al., 2010, West et al., 2008, Barber et al., 2013), we considered whether the increases in cone precursor integration observed in the *Nrl*^*−/−*^ might be explained by its disturbed cytoarchitecture. A variant of the *Nrl*^*−/−*^ model, the *Nrl*^*−/−*^;*RPE65*^*R91W/R91W*^ mouse, carries an additional point mutation on the *RPE65* gene, which is reported to prevent the appearance of whorls and rosettes and preserve the OLM (Samardzija et al., 2014). We performed ultrastructural analysis (Figure 5F) and IHC (Figure 5G) analysis of both the *Nrl*^*−/−*^ and *Nrl*^*−/−*^;*RPE65*^*R91W/R91W*^ retina. In the *Nrl*^*−/−*^, the adherens junctions are much sparser compared with wild-type, with the majority forming between Müller glial cells (Figure 5F, blue arrows; and A.B.G. and R.A.P., unpublished data). These alterations were particularly evident around regions of rosette formation. Conversely, rosettes were absent in the *Nrl*^*−/−*^;*RPE65*^*R91W/R91W*^ retina, as previously reported, although there were some subtle disturbances in ONL lamination (Figure 5F, blue asterisk). Despite this, ultrastructural analysis demonstrated the presence of typical photoreceptor-Müller glial cell adherens junctions, indicating that the OLM is largely intact in this model (Figure 5F, blue arrows). A significantly lower number of GFP^+^ cells was seen in the host ONL following transplantation of P8 *Nrl*^*−/−*^;*Nrl-*GFP donor cells into the *Nrl*^*−/−*^;*RPE65*^*R91W/R91W*^ model, compared with *Nrl*^*−/−*^ hosts (584 ± 116; N = 10/14; compared with 4,631 ± 971, N = 19/23; Figures 5A and 5D). Moreover, the proportion of these cells that were Y^+^ was significantly lower than in the *Nrl*^*−/−*^ host (6% ± 1%; N = 6) (Figures 5A and 5E) and more similar to that seen in wild-type hosts.

Together, these data indicate that the retinal environment of *Nrl*^*−/−*^ and *Prph2*^*rd2/rd2*^ hosts is able to support donor cone photoreceptor integration alongside material transfer, while the wild-type retina supports only very limited cone integration. The cytoarchitecture of the host retina is likely to play a major role in determining the relative contributions of these two mechanisms to transplantation outcome.

## Discussion

The transplantation of healthy photoreceptor precursors, derived from both stem cells and donor retinas, has been shown to restore aspects of visual function in animal models of retinal degeneration (Santos-Ferreira et al., 2015, Pearson et al., 2012, Singh et al., 2013, Barnea-Cramer et al., 2016). Previously, this was understood to occur by a process of donor cell migration and integration within the partially intact recipient retina. However, we (Pearson et al., 2016) and others (Santos-Ferreira et al., 2016, Singh et al., 2016) have recently shown that instead, when transplanted into the non-degenerative retina, where host photoreceptors remain, donor rod photoreceptors engage in a process of material transfer with host photoreceptors, which appears to lead to the exchange of RNA and/or protein in a robust, transient, and repeatable manner (Pearson et al., 2016).

Much less is known about the transplantation of cone photoreceptors and whether they could engage in a similar process. Early studies on cone transplantation reported a preponderance of GFP-labeled cells that morphologically resembled rods (Lakowski et al., 2010) but, since a mixed population of rod and cone donors was used, it was hypothesized that this may represent a change in cell fate or a preference of rods to integrate over cones. Recently, others reported similar findings using a variety of cone and cone-like donor cells (Santos-Ferreira et al., 2015, Smiley et al., 2016). In light of our recent findings regarding material transfer between rods, we sought to determine whether the rod-like cells seen after transplantation of cones also arise from material transfer. Here, we demonstrate that, irrespective of their origin, cone photoreceptors can engage in material transfer with host rod and cone photoreceptors. Given that this occurred both for stem cell-derived cones and genetically engineered cone-like cells, material transfer is likely to account for all previous reports of rod-like phenotypes following cone transplantation into the intact, non-degenerative murine retina (Smiley et al., 2016, Santos-Ferreira et al., 2015, Lakowski et al., 2010). Importantly, however, we also find that cone donors are capable of reasonably efficient integration when the host retinal structure is disrupted. This is important in the light of the recent publications on material transfer. It suggests that it may be possible to improve the levels of integration with appropriate manipulations of the host environment, as previously envisaged (see Pearson, 2014). It also highlights the need for careful characterization of the relative contributions each made by material transfer and donor integration when assessing transplantation outcome in future studies.

The recent reports of material transfer between donor and host photoreceptors (Santos-Ferreira et al., 2016, Pearson et al., 2016, Decembrini et al., 2017, Singh et al., 2016, Ortin-Martinez et al., 2017) requires a significant re-evaluation of the cellular mechanisms underlying rescue by photoreceptor transplantation, at least in those recipients where some host photoreceptors remain. While the mechanisms are at present unknown, this current study and other recently published papers begin to provide some direction. Transplantation outcome, defined as the number of GFP^+^ cells within the host ONL, is dependent upon the developmental stage of the donor cell and that post-mitotic cells yielded better outcomes than progenitors (Pearson et al., 2012, Lakowski et al., 2010, MacLaren et al., 2006, Gonzalez-Cordero et al., 2013). Although we now understand the predominant mechanism to be material transfer, the developmental correlation with this process remains true: post-mitotic rods (Pearson et al., 2016, Santos-Ferreira et al., 2016) and cones derived from post-natal stages engage in material transfer more effectively than immature retinal cells. In a very recent report, Arsenijevic and colleagues (Decembrini et al., 2017) transplanted *Chrnb4*-EGFP donor-derived cones, as we have done here, and similarly concluded that material transfer is a developmentally regulated phenomenon.

A comparison of the findings reported here and by others (Smiley et al., 2016, Ortin-Martinez et al., 2017, Decembrini et al., 2017) following transplantation of various cone and cone-like populations into the intact wild-type retina, with those following transplantation of rod photoreceptors (Santos-Ferreira et al., 2016, Pearson et al., 2016) shows that the numbers of GFP^+^ cells within the host ONL are much lower using cone, compared with rod, donors (hundreds versus many thousands). This might indicate that material transfer is less efficient from donor cones to host rods (and cones). Alternatively, material transfer may require the continued presence of donor cells in the SRS and cones may survive less well than rods, or a combination of these. Supporting the need for donor survival, we recorded a surprisingly high number of host eyes with very few donor cones within the SRS, compared with equivalent transplants made with rod or mixed populations of donors (Pearson et al., 2012, Pearson et al., 2016, Lakowski et al., 2010) or indeed the genetically engineered cone-like donor populations. Previously, we reported that manipulation of the immune system prolonged the presence of GFP^+^ cells in the wild-type host ONL (West et al., 2010), a finding that can be, perhaps, alternatively explained by prolonging the survival of donor cells available to provide material for transfer. Similarly, two other recent reports reported that modulation of the host immune environment permitted sustained survival of transplanted cells in the SRS (Zhu et al., 2017, Neves et al., 2016), and, from our interpretation of the published images, what appears to include robust levels of material transfer (see also MacLaren, 2017). The dependency on donor developmental stage and the apparent need for the sustained presence of donor cells may provide clues as to the cellular mechanisms underlying material transfer.

Transplants into wild-type hosts showed surprising heterogeneity with respect to cone marker expression by the GFP^+^ cells, with variable expression of CARR and very rare expression of RXRγ. Wallace and colleagues reported minimal cone marker expression (Smiley et al., 2016), while Ader and colleagues reported expression of a number of cone markers, although they did not indicate how common such expression was (Santos-Ferreira et al., 2015). Future investigations will require careful quantitative analysis of the marker expression profile of both donor cells and GFP^+^ within the host; these will be important to our understanding of what can, and cannot, be exchanged by material transfer and what the broader implications of material transfer and its potential utility might be.

As previously observed for rod transplantation (Barber et al., 2013), we report here that the efficacy of cone transplantation, as determined by the number of GFP^+^ cells in the host ONL, is critically dependent on the host environment. Non-degenerative models behave like wild-type hosts, resulting in the presence of small numbers of GFP^+^ cells within the ONL, the majority arising through material transfer. In another recent study, Arsenijevic and colleagues (Decembrini et al., 2017) transplanted *Chrnb4*-EGFP^+^ cones in the *Cnga3*^*−/−*^ and the *Nrl*^*−/−*^*RPE65*^*R91W/R91W*^ mouse lines. They reported low numbers of reporter-labeled cells, similar to what we report here following transplantation of genetically engineered cone-like cells into the same models. They note that GFP^+^ cell numbers could be higher in localized areas and propose that these may reflect areas of OLM disruption, possibly through injection trauma. This fits with our own observations that significantly higher numbers of GFP^+^ cells were found in the *Nrl*^*−/−*^ and *Prph2*^*rd2/rd2*^ host retinas, both models that display highly disrupted OLM (this study; also Samardzija et al., 2014, Hippert et al., 2015, Barber et al., 2013). These models are also unusual in that they are composed largely of cone, rather than rod, cells. This might suggest that a cone-enriched environment better supports cone integration. Arguing against this, however, are the low levels of integration seen in the *Nrl*^*−/−*^*RPE65*^*R91W/R91W*^ mouse retina, which is also cone enriched but whose cytoarchitecture is largely intact.

Interestingly, Wallace and colleagues (Ortin-Martinez et al., 2017) transplanted *Ccdc136-*GFP^+^ (cones) and *Nrl*^*−/−*^*Ccdc136-*GFP^+^ (cone-like) cells into the *Nrl*^*−/−*^ host retina and, like we report here, noted a significant increase in the number of GFP cells in the *Nrl*^*−/−*^ hosts compared with wild-type. However, they concluded that this increase was due entirely to an increase in material transfer and that this preferentially occurred in regions of OLM disruption. Their conclusions were based on the use of 5-ethynyl-2′-deoxyuridine (EdU) to pre-label a proportion of the donor cone nuclei and the failure to see any GFP^+^ cells within the host ONL that also bore EdU. In contrast, by using FISH to label the Y chromosome of male donors, we could conclude that at least a proportion of the increase in GFP^+^ cells in the host *Nrl*^*−/−*^ ONL is the result of actual donor cell integration within the host retina. The reasons for the discrepancy are not clear. Some reports have described potentially toxic effects of EdU, and we (Warre-Cornish et al., 2014) and others (Andersen et al., 2013, Ligasova et al., 2015, Neef and Luedtke, 2011) have reported detrimental effects on cell migration and survival. It is possible that such effects may account for the absence of integrated cells in the study by Wallace and colleagues (Ortin-Martinez et al., 2017). Conversely, our experimental design makes use of an endogenous marker revealed after transplantation, which is less likely to have any unintended impact upon the normal behavior of transplanted photoreceptors. Alternatively, the age of the host animal and concomitant stage of degeneration at the time of transplantation may affect the degree of donor cell integration, but may not be material transfer, as other potential barriers, such as glial hypertrophy, may start to impede donor cell integration (Hippert et al., 2015, Hippert et al., 2016, Barber et al., 2013). Regardless, it is important to emphasize that the increase seen in this model is not due *only* to increased integration. A significant proportion of the increase in GFP^+^ cells in *Nrl*^*−/−*^, compared with wild-type, must be due also to increased material transfer, alongside increased integration. Poor transplantation outcomes, which we currently assume encompasses a varying proportion of material transfer and donor cell integration, are often correlated with models that have high levels of CSPG deposition (Hippert et al., 2015, Barber et al., 2013) and/or maintain OLM integrity. Conversely, disruption of the OLM (Pearson et al., 2010, West et al., 2008) and breakdown of CSPGs (Singhal et al., 2008, Barber et al., 2013, Suzuki et al., 2007) each facilitate better transplantation outcomes. Future work needs to determine to what extent the parallel processes of integration and material transfer contribute to transplantation outcome. Moreover, elucidation of the cellular mechanisms behind material transfer will help establish how manipulations of the degenerative environment act to improve the efficiency of this unusual and surprising process.

Recent reports have described the transplantation of genetically engineered cone-like cells (this study and Smiley et al., 2016, Santos-Ferreira et al., 2015, Ortin-Martinez et al., 2017, Decembrini et al., 2017) and their ability to rescue cone-mediated function (Santos-Ferreira et al., 2015). Similarly, we reported restoration of rod-mediated function following the transplantation of rod donor cells into the intact, but non-functional, *Gnat1*^*−/−*^ recipient (Pearson et al., 2012). In view of the recent findings, it is likely that these rescues were achieved by material transfer mediating the restoration of functional levels of the proteins missing in the host photoreceptors. This opens new interesting avenues of investigation, as it may be possible to harness the mechanisms mediating material transfer for the restoration of visual function in progressive retinal degeneration. Regarding the current study, the numbers of GFP^+^ cells found following cone transplantation is markedly lower than that seen for rod transplantation, even in the *Nrl*^*−/−*^ host. For this reason, we did not explore whether these cells were functional, although others have reported some restoration of function following transplantation of the same donor population (*Nrl*^*−/−*^ [Santos-Ferreira et al., 2015]). In the current study we find a significant increase in the number of true integration events, compared with material transfer; it will be of significant future interest to determine to what degree these cells are capable of contributing to vision, compared with those resulting from material transfer.

Together, our data demonstrate that the transplantation of cone photoreceptors results in both material transfer and donor cell integration, and the relative contribution of these two processes is likely to depend on the etiology of disease and the host retinal environment.

## Experimental Procedures

All animal studies were carried out under the Animals (Scientific Procedures) Act 1986 under a project license PPL 70/8120 issued by the UK Government Home Office and in accordance with protocols approved by the Animal Welfare and Ethics Committee of the UCL Institute of Ophthalmology.

All means are stated ±SD except for cell counts, which are stated as ±SEM. N denotes number of eyes examined and n the number of cells, where appropriate. Full details of experimental methods are provided in Supplemental Experimental Procedures.

### Mouse ESC Culture and Retinal Differentiation

Retinal differentiation was achieved using a mouse EK.CCE ESC line (Evans and Kaufman, 1981) and an adapted 2D/3D culture system, as described in Kruczek et al. (2017), which includes the addition of 1 mM taurine and 500 nM retinoic acid from day 14 of culture onward. Embryoid bodies were labeled with ShH10.L/MOpsin.GFP on day 20 of culture and dissociated for transplantation on days 26–30.

### Dissociation and Fluorescence-Activated Cell Sorting

Embryonic or postnatal neural retinas or mESC-derived oganoids were dissociated using a papain-based Neural Tissue Dissociation Kit (Miltenyl Biotec) prior to sorting on a BD Influx Cell Sorter (Becton Dickinson). Flow-sorted GFP^+^ cells were on average >95% pure and >80% viable. Cells were resuspended at a final concentration of 200,000 live cells/μL in sterile EBSS and DNase I (50 U/mL) before injection, unless otherwise stated.

### Transplantation of Donor Photoreceptors

One microliter of cell suspension was injected subretinally into the superior retina. Adult mice from different strains were used at ages as denoted in Table S1. All animals were housed under a normal 12/12-hr light/dark cycle. Eyes were harvested 2 weeks after transplantation.

### Immunohistochemistry and Cell Counts

Eyes were fixed for 30 min in 4% formaldehyde, washed with PBS, and incubated overnight in 20% (w/v) sucrose, prior to embedding in OCT matrix. Tissue was cut into 18-μm cryosections mounted on glass slides, air-dried for 20 min, and kept frozen at −20°C for use in immunostaining. Staining protocol and the antibodies used are listed in Supplemental Experimental Procedures.

Cell counts were performed in a blinded manner after immunostaining with fluorescein isothiocyanate-conjugated anti-GFP antibody. In every third cryosection, all GFP^+^ cells with cell bodies located in the ONL were counted and assessed, when possible, for co-staining and morphology. The total number of cells for each injected eye was calculated as three times this count.

## Author Contributions

P.V.W. contributed to the conception, design, execution, and analysis of most experiments; F.d.M. contributed to the execution and analysis of several of the donor-derived transplantation experiments; K.K. contributed to the design, execution, and analysis of all mESC-derived transplantation experiments; J.R. designed and executed experiments regarding FISH and contributed to histological processing; A.B.G. designed and executed OLM experiments; C.H. generated mouse lines; N.D.A., G.G., A.A.K., and D.G. contributed to transplantation experiments; A.C.B. performed the *OPN1LW*-EGFP experiments; Y.D. contributed to histological processing and surgery; S.J.L.B. and M.K. contributed to ESC maintenance and histological processing; E.Z.A. contributed to cell preparations and animal maintenance; R.D.S. performed FACS and flow analysis; J.W.B.B. contributed to surgery; A.J.S. and A.G.-C. contributed to experimental design and interpretation of experiments; R.R.A. and J.C.S. contributed to the conception and design of experiments, manuscript writing, and funding; R.A.P. contributed to the conception, design, execution, analysis, and interpretation of experiments, subretinal surgery, and funding and wrote the manuscript.

## Supplemental Information

Document S1. Supplemental Experimental Procedures, Figures S1–S3, and Tables S1–S7

Document S2. Article plus Supplemental Information
