# Supplementary material for: Transplanted Donor- or Stem Cell-Derived Cone Photoreceptors Can Both Integrate and Undergo Material Transfer in an Environment-Dependent Manner
Source: Stem Cell Reports. 2018 Jan 4;10(2):406–21. doi: 10.1016/j.stemcr.2017.12.008 (PMC5830910; doi:10.1016/j.stemcr.2017.12.008)
Supplement: Document S1. Supplemental Experimental Procedures, Figures S1–S3, and Tables S1–S7 [file mmc1.pdf]

Stem Cell Reports, Volume *10*

Supplemental Information

Transplanted Donor- or Stem Cell-Derived Cone Photoreceptors Can Both Integrate and Undergo Material Transfer in an Environment-Dependent Manner

Paul V. Waldron, Fabiana Di Marco, Kamil Kruczek, Joana Ribeiro, Anna B. Graca, Claire Hippert, Nozie D. Aghaizu, Aikaterini A. Kalargyrou, Amanda C. Barber, Giulia Grimaldi, Yanai Duran, Samuel J.I. Blackford, Magdalena Kloc, Debbie Goh, Eduardo Zabala Aldunate, Robert D. Sampson, James W.B. Bainbridge, Alexander J. Smith, Anai Gonzalez-Cordero, Jane C. Sowden, Robin R. Ali, and Rachael A. Pearson

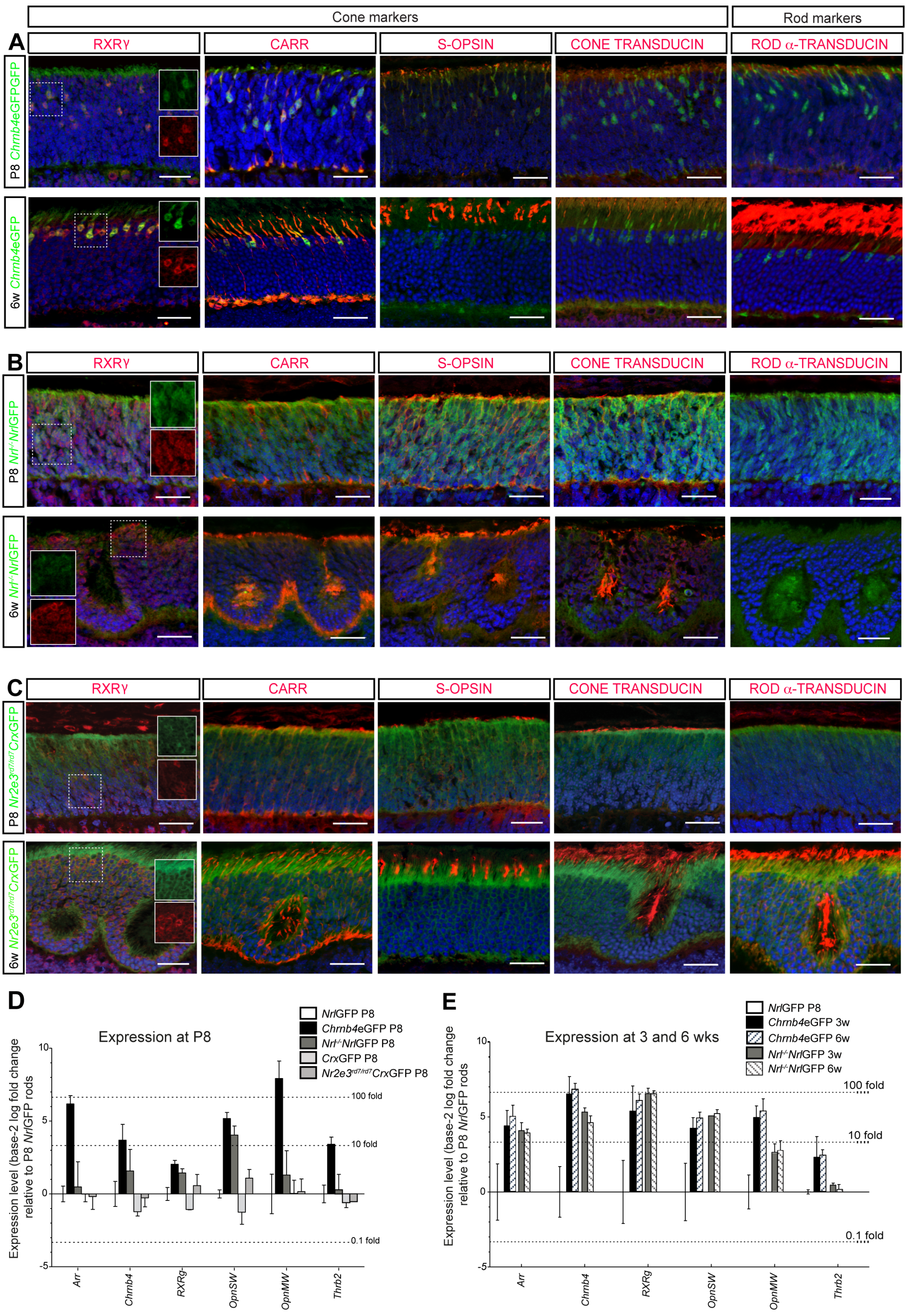

Cone markers
Rod markers
A
RXRγ
CARR
S-OPSIN
CONE TRANSDUCIN
ROD α-TRANSDUCIN
P8 Chrnb4eGFPGFP
6w Chrnb4eGFP
B
P8 Nrl-/-NrlGFP
6w Nrl-/-NrlGFP
C
P8 Nr2e3rd7/rd7CrxGFP
6w Nr2e3rd7/rd7CrxGFP
D
Expression at P8
NrlGFP P8
Chrnb4eGFP P8
Nrl-/-NrlGFP P8
CrxGFP P8
Nr2e3rd7/rd7CrxGFP P8
Expression level (base-2 log fold change relative to P8 NrlGFP rods)
100 fold
10 fold
0.1 fold
Arr
Chrnb4
RXRg
OpnSW
OpnMW
Thrb2
E
Expression at 3 and 6 wks
NrlGFP P8
Chrnb4eGFP 3w
Chrnb4eGFP 6w
Nrl-/-NrlGFP 3w
Nrl-/-NrlGFP 6w

Figure S1. Immunohistological characterization of mouse lines used for donor cell generation, related to Figs. 1-3. Images show confocal projections of the central retina of postnatal day 8 and 6 wk (adult) old (**A**) *Chrnb4*eGFP, (**B**) $Nrl^{-/-}$*;NrlGFP,* and (**C**) $Nr2e3^{rd7/rd7}$*;Crx*GFP mice. Retinal sections were stained for the cone markers RXRγ, CONE ARRESTIN (CARR), S-OPSIN, CONE TRANSDUCIN (*Gnat2*) and the rod marker ROD α-TRANSDUCIN (*Gnat1*). Note that not all photoreceptor cells in the $Nrl^{-/-}$*;NrlGFP, and* $Nr2e3^{rd7/rd7}$*;CrxGFP* lines appear to express a full complement of cone markers in the adult. Scale bar 25µm. **D, E,** Quantitative PCR analysis of cone marker gene expression in GFP-expressing donor populations. (**D**) relative expression levels of cone genes in GFP+ cells isolated from the different donor retinae at P8, compared to P8 *Nrl*GFP retina cells. (**E**) changes in relative expression levels of cone genes in *Chrnb4e*GFP cones and $Nrl^{-/-}$*;NrlGFP* cone-like cells between 3 and 6 wks of age, compared to *Nrl*GFP rod precursor cells. Adult cells were taken from mice between 5.5 - 9 weeks old. Values given are -ΔΔCt, the difference in signal detection found for the gene and the housekeeping gene β-actin, compared to that in *Nrl*GFP cells of similar age, which was set to zero. This corresponds to the base-2 log of the fold change. Measurements were taken as the means of duplicates and the plotted values are +/- S.D. Statistical analysis is shown in **Supplemental Tables 1-4**.

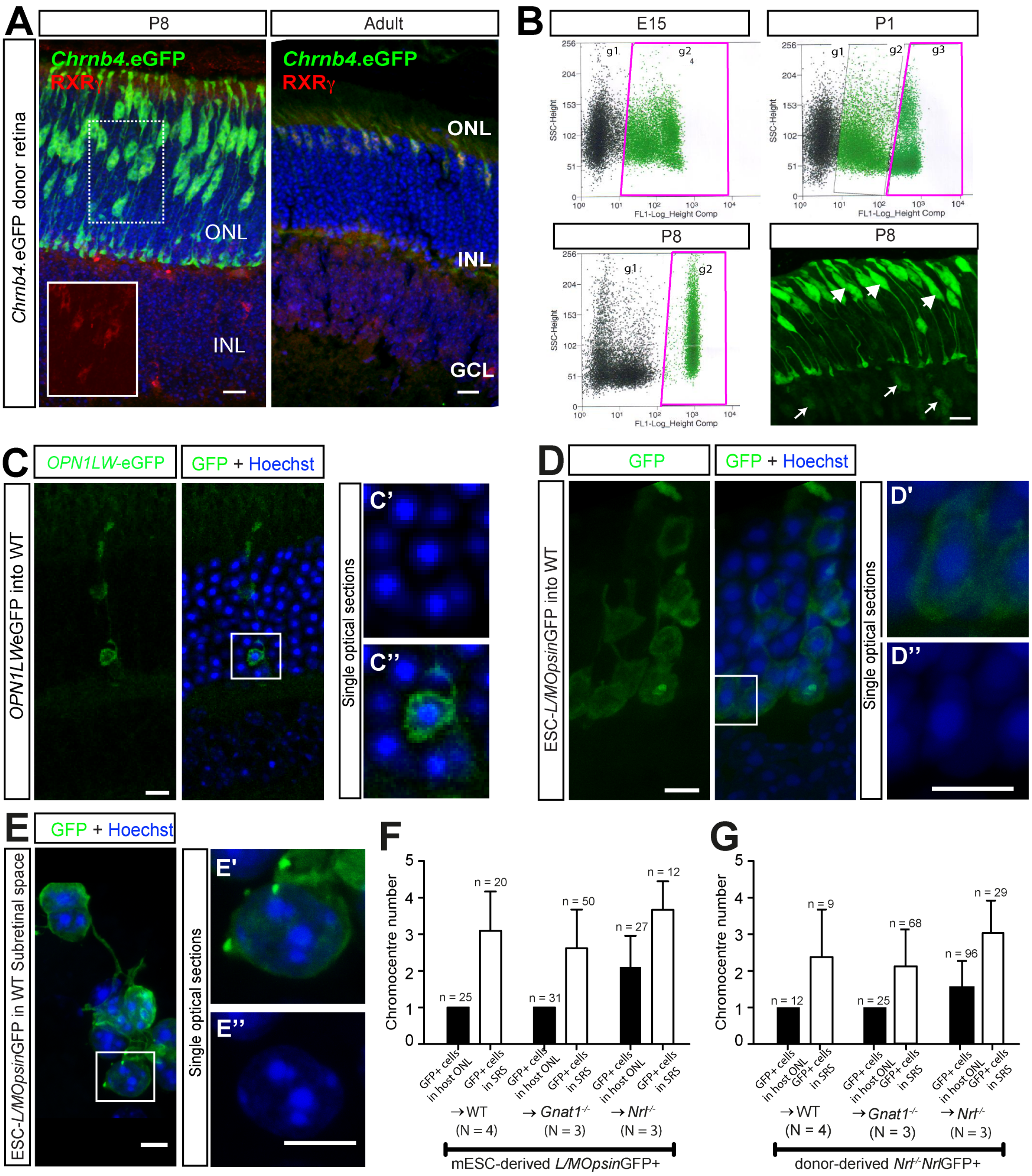
A
P8
Adult
Chrnb4.eGFP donor retina
Chrnb4.eGFP
RXRγ
ONL
INL
GCL
B
E15
P1
P8
g1
g2
g3
SSC-Height
FL1-Log_Height Comp
C
OPN1LW-eGFP
GFP + Hoechst
OPN1LWeGFP into WT
Single optical sections
C'
C''
D
GFP
GFP + Hoechst
ESC-L/MOpsinGFP into WT
Single optical sections
D'
D''
E
GFP + Hoechst
ESC-L/MOpsinGFP in WT Subretinal space
Single optical sections
E'
E''
F
Chromocentre number
n = 25
n = 20
n = 31
n = 50
n = 27
n = 12
GFP+ cells in host ONL
GFP+ cells in SRS
→WT (N = 4)
→ Gnat1-/- (N = 3)
→ Nrl-/- (N = 3)
mESC-derived L/MOpsinGFP+
G
Chromocentre number
n = 12
n = 9
n = 25
n = 68
n = 96
n = 29
GFP+ cells in host ONL
GFP+ cells in SRS
→WT (N = 4)
→Gnat1-/- (N = 3)
→ Nrl-/- (N = 3)
donor-derived Nrl-/-NrlGFP+

Figure S2. Transplantation of donor- and mESC-derived GFP+ cone precursors leads to the presence of small numbers of predominantly rod-like cells within the host ONL, related to Fig. 1. (A) confocal images of P8 and adult *Chrnb4*-eGFP retinal sections co-stained with the cone and ganglion cell marker Rxr- γ. The $[GFP]_{high}$ cells become restricted to the apical margin of the ONL and are RXRγ+. $[GFP]_{low}$ cells were predominantly found in the inner retina. (**B**) FACS plots show the presence of different populations of GFP+ cells within the *Chrnb4*-EGFP+ population during embryonic and early postnatal development. Magenta boxes denote the population collected for transplantation at each age. **C-D**) confocal images of rod-like GFP+ cells within host ONL after transplantation of (**C**) *OPN1LW*-eGFP+ donor-derived cone photoreceptors and (**D**) mESC-derived *L/MOpsin*-GFP+ cone precursors. (**E**) confocal image of mESC-derived GFP+ cells within host subretinal space after transplantation. (**F-G**) chromocentre counts for GFP+ cells within the ONL and subretinal space of wildtype, *Gnat1*$^{-/-}$ and *Nrl*$^{-/-}$ hosts following transplantation of, (**F**) mESC-derived or (**G**) *Nrl*$^{-/-}$*Nrl*GFP donor-derived cone precursors. N = no. of eyes; n = no. of cells. Stats not applied. Scale bars 10 μm (**B, C, G**) and 5 μm (**E-F**).

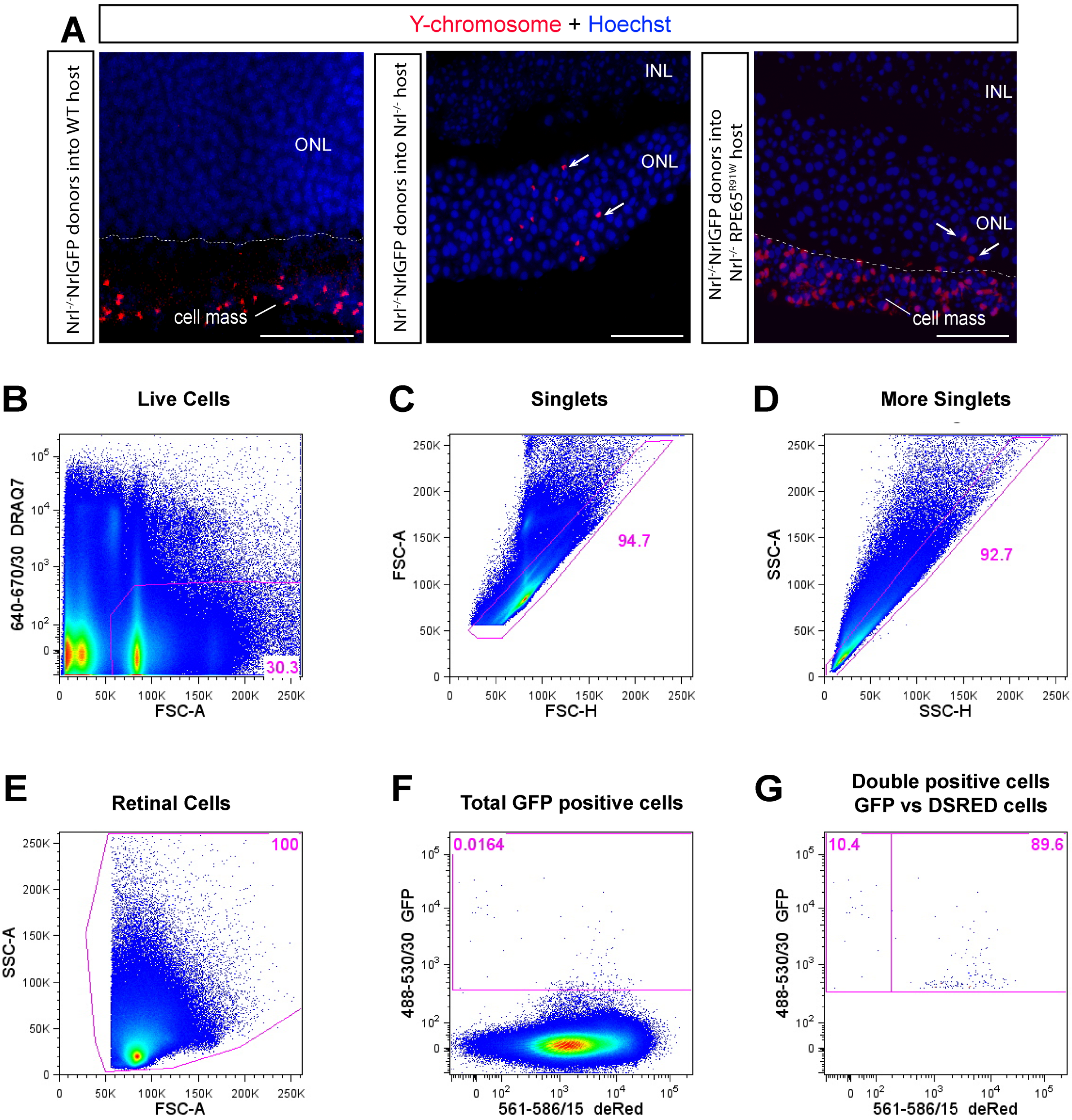
A
Y-chromosome + Hoechst
Nrl-/-NrlGFP donors into WT host
ONL
cell mass
Nrl-/-NrlGFP donors into Nrl-/- host
INL
ONL
Nrl-/-NrlGFP donors into Nrl-/- RPE65R91W host
INL
ONL
cell mass
B
Live Cells
640-670/30 DRAQ7
FSC-A
30.3
C
Singlets
FSC-A
FSC-H
94.7
D
More Singlets
SSC-A
SSC-H
92.7
E
Retinal Cells
SSC-A
FSC-A
100
F
Total GFP positive cells
488-530/30 GFP
561-586/15 deRed
0.0164
G
Double positive cells
GFP vs DSRED cells
488-530/30 GFP
561-586/15 deRed
10.4
89.6

Figure S3. FISH and Flow cytometry analysis of integration and material transfer, related to Fig. 3 and 5 and Supplemental Methods. A, Cone photoreceptors can integrate in some models of retinal degeneration. Confocal images of FISH labelling, showing significant numbers of Y-chromosome+ (*red*) nuclei in $Nrl^{-/-}$ host ONL but few, if any, in wildtype or $Nrl^{-/-};RPE65^{R91W/R91W}$ host ONL, after transplantation of P8 $Nrl^{-/-};Nrl$GFP donors. Cell masses located in SRS also demonstrate widespread labelling. **B-G,** Flow cytometry gating strategy for analysis of host (*DsRed* or $Nrl^{-/-};DsRed$) and donor-derived ($Nrl^{-/-};NrlGFP$) reporter proteins following transplantation. This example displays a *DsRed* host retina examined by flow cytometry 2-3 wks post-transplantation of $Nrl^{-/-};NrlGFP$ post-mitotic cone-like donor cells. (**B**) representative plot showing a dissociated retina stained for SYTOX Blue Dead Cell Stain™ used to exclude dead cells and cellular debris and identify the live cell population. (**C-D**) plots showing the exclusion of cellular aggregates to isolate single cells. (**E**) a representative plot depicting the relative size and granularity of single, live retinal cells. (**F-G)** plots of a representative transplanted sample showing (**F**) total GFP^+ cells (*pink box*) and (**G**) subsequent gating showing the % of GFP^+-only (*left pink box*) and $GFP^+/DsRed^+$ (*right pink box*). Gates were set based on controls, as presented in **Fig. 3** in the main text. Abbreviation: MFI (mean fluorescent intensity).

Supplemental Tables 1-4. qPCR comparisons of the expression of various cone and rod photoreceptor-specific marker genes in developing (P8) and Adult FACS purified GFP+ cells. Cell sources chosen for comparison are cone photoreceptors from *Chrnb4*eGFP retinas, cone like photoreceptors from *Nrl*$^{-/-}$*NrlGFP*, a mixed photoreceptor population from *Crx*GFP and a mixed photoreceptor population with increased numbers of cone like cells from *Nr2e3*$^{rd7/rd7}$*Crx*GFP. For *Chrnb4*eGFP and *Nrl*$^{-/-}$*NrlGFP* cells, 3 wk old cells were also obtained and compared with Adult NrlGFP cells. Groupwise and individual comparisons can be found in the tables below. Separate comparisons with *Nrl*GFP cells were made with *Chrnb4*eGFP, and with *Nrl*$^{-/-}$*NrlGFP* cells. *Nr2e3*$^{rd7/rd7}$*Crx*GFP cells were compared with *Crx*GFP cells as a closer control. Tables show the results of two-way ANOVAs, the effects of Cell type, Gene, and the interaction between the two, and post-test comparisons. Statistical comparisons are made using the base-2 log of the fold change of expression, as shown in **Supplemental Fig. S1D, E**.

**Chrnb4.eGFP - P8**	**Cone markers**					
	Effects					
	Cell		Gene		Interaction	
	F(1,54)	p	F(5,54)	p	F(5,54)	p
	636.62	<0.0001 ****	21.39	<0.0001 ****	21.39	<0.0001 ****
	Post-test comparisons (Bonferroni-corrected) – significance with p values					
	Arr	*Chrnb4*	*Rxrγ*	*OpnSW*	*OpnMW*	*Thrβ2*
	<0.0001	<0.0001	<0.001	<0.0001	<0.0001	<0.0001
**Chrnb4.eGFP - 3 weeks and Adult**	**Cone markers**					
	Effects					
	Cell		Gene		Interaction	
	F(2,36)	p	F(5,36)	p	F(10,36)	p
	100.46	<0.0001 ****	5.83	0.0005 ****	1.49	0.184 n.s.
	Post-test comparisons (Bonferroni-corrected) – significance with p values					
	Arr	*Chrnb4*	*Rxrγ*	*OpnSW*	*OpnMW*	*Thrβ2*
	3 wks: <0.001	<0.0001	<0.0001	<0.01<0.001	<0.001	n.s.
	6 wks: <0.001	<0.0001	<0.0001	<0.001	<0.0001	n.s.

Table S1. qPCR comparisons of cone marker expression between (top) P8 Chrnb4.eGFP and P8 NrlGFP flow sorted cells and (bottom) between 3 wk and adult Chrnb4.eGFP and adult NrlGFP flow sorted cells

<table>
<tr><td rowspan="10">Chrnb4.eGFP - P8</td><td colspan="6">Rod markers</td></tr>
<tr><td colspan="6">Effects</td></tr>
<tr><td colspan="2">Cell</td><td colspan="2">Gene</td><td colspan="2">Interaction</td></tr>
<tr><td>F(1,27)</td><td>p</td><td>F(2,27)</td><td>p</td><td>F(2,27)</td><td>p</td></tr>
<tr><td>13</td><td>0.0012
**</td><td>0.06</td><td>0.9384
n.s</td><td>0.07</td><td>0.936
n.s.</td></tr>
<tr><td colspan="6"></td></tr>
<tr><td colspan="6">Post-test comparisons (Bonferroni-corrected) – significance with p values</td></tr>
<tr><td colspan="2">Nrl</td><td colspan="2">Nr2e3</td><td colspan="2">Rhodopsin</td></tr>
<tr><td colspan="2">n.s</td><td colspan="2">n.s</td><td colspan="2">n.s</td></tr>
<tr><td colspan="6" style="display:none"></td></tr>
<tr><td rowspan="10">Chrnb4.eGFP - 3 weeks and Adult</td><td colspan="6">Rod markers</td></tr>
<tr><td colspan="6">Effects</td></tr>
<tr><td colspan="2">Cell</td><td colspan="2">Gene</td><td colspan="2">Interaction</td></tr>
<tr><td>F(2,18)</td><td>p</td><td>F(2,18)</td><td>p</td><td>F(2,18)</td><td>p</td></tr>
<tr><td>48.11</td><td><0.0001
****</td><td>0.27</td><td>0.7694
n.s.</td><td>0.13</td><td>0.9679
n.s.</td></tr>
<tr><td colspan="6"></td></tr>
<tr><td colspan="6">Post-test comparisons (Bonferroni-corrected) – significance with p values</td></tr>
<tr><td colspan="2">Nrl</td><td colspan="2">Nr2e3</td><td colspan="2">Rhodopsin</td></tr>
<tr><td colspan="2">3 wks: n.s</td><td colspan="2">n.s</td><td colspan="2"><0.05</td></tr>
<tr><td colspan="2">6 wks: <0.001</td><td colspan="2"><0.0001</td><td colspan="2"><0.001</td></tr>
</table>

Table S2. qPCR comparisons of rod marker expression (top) between P8 *Chrnb4*-eGFP and P8 *Nrl*GFP flow sorted cells and (bottom) between 3 wk and adult *Chrnb4*-eGFP and adult *Nrl*GFP flow sorted cells

**$Nrl^{-/-}$NrlGFP - P8**	**Cone markers**					
	Effects					
	Cell		Gene		Interaction	
	F(1,42)	p	F(5,42)	p	F(5,42)	P
	30.37	<0.0001	3.97	<0.0049	3.97	0.0049
	Post-test comparisons (Bonferroni-corrected) – significance with p values					
	Arr	*Chrnb4*	*Rxrγ*	*OpnSW*	*OpnMW*	*Thrβ2*
	n.s	n.s	n.s	<0.0001	n.s	n.s
**$Nrl^{-/-}$NrlGFP – 3 weeks and adult**	**Cone markers**					
	Effects					
	Cell		Gene		Interaction	
	F(2,36)	p	F(5,36)	p	F(10,36)	p
	95.81	<0.0001 ****	19.67	<0.0001 ****	5	0.0002 ***
	Post-test comparisons (Bonferroni-corrected) – significance with p values					
	Arr	*Chrnb4*	*Rxrγ*	*OpnSW*	*OpnMW*	*Thrβ2*
	3 wks: <0.001	<0.0001	<0.0001	<0.0001	<0.05	n.s.
	6 wks: <0.001	<0.0001	<0.0001	<0.0001	<0.05	n.s.

Table S3. qPCR comparisons of cone marker expression (top) between P8 *$Nrl^{-/-}$Nrl*GFP and P8 *Nrl*GFP flow sorted cells and (bottom) between 3 wk and adult *$Nrl^{-/-}$Nrl*GFP and adult *Nrl*GFP flow sorted cells.

**Nr2e3$^{rd7/rd7}$CrxGFP - P8**	**Cone markers**					
	Effects					
	Cell		Gene		Interaction	
	F(1,24)	p	F(5,24)	p	F(5,24)	p
	4.81	0.0382 *	0.49	0.7837 n.s	1.18	0.3494 n.s
	Post-test comparisons (Bonferroni-corrected) – significance with p values					
	Arr	Chrnb4	Rxrγ	OpnSW	OpnMW	Thrβ2
	n.s	n.s	n.s	n.s	n.s	n.s
**Nr2e3rd7/rd7CrxGFP - Adult**	**Cone markers**					
	Effects					
	Cell		Gene		Interaction	
	F(1,24)	p	F(5,24)	p	F(5,24)	p
	10.64	0.0033 **	11.05	<0.0001 ****	0.85	0.5271 n.s.
	Post-test comparisons (Bonferroni-corrected) – significance with p values					
	Arr	*Chrnb4*	*Rxrγ*	*OpnSW*	*OpnMW*	*Thrβ2*
	6 wks: n.s.	n.s.	n.s.	n.s.	n.s.	n.s.

Table S4. qPCR comparisons of cone marker expression (top) between P8 *Nr2e3*$^{rd7/rd7}$*Crx*GFP and P8 *Crx*GFP flow sorted cells and (bottom) between adult *Nr2e3*$^{rd7/rd7}$*Crx*GFP and adult *Crx*GFP flow sorted cells.

Mouse model	Donor/ Recipient	Key features	Degeneration	Age at time of use
*Nrl*GFP	Donor	Normal retina, all rods are GFP+	None	E15.5 - P8
$Nrl^{-/-}$*Nrl*GFP	Donor	Cone-like (‘cod’) only retina. All cone-like cells are GFP+, true cones are GFP-	Mild	E15.5 - P8
$Nr2e3^{rd7/rd7}$ *Crx*GFP	Donor	Normal cones and supra-normal numbers of S cone-like hybrid cells. All PRs are GFP+	Mild, late	E15.5 - P8
*Chrnb4*GFP	Donor	Normal retina. All cones are GFP+ (RGCs also weakly GFP+)	None	E15.5 - P8
Wildtype (*C57Bl/6*)	Recipient	Normal retina	None	2 - 3 mnth
$Prph2^{rds/rds}$	Recipient	Inherited RP model. Moderate rate of degeneration - 50% loss of rods by 8 wks. Secondary loss of cones	Moderate	2 - 3 mnth
$Nrl^{-/-}$	Recipient	Cone-like (‘cod’) only retina. Rosettes, mild degeneration completed by ~6wks	Mild	2 - 3 mnth
$Nr2e3^{rd7/rd7}$	Recipient	Cone-only retina comprising mix of cods and supra-normal number of S cones. Mild degeneration at very late stages	Mild, late	2 - 4 mnth
cpfl5	Recipient	Missense mutation in CNGA3 gene. Cones are non-functional and degenerate over a period of 2-5 months. Loss of S cones precedes M/L cones	Moderate (cones only)	2 - 4 mnth
cpfl1	Recipient	$PDE6c^{cpfl1}$ spontaneous mutation causing frame shift and premature stop in PDE6c. Non-functional cones, which degenerate over a period of 5 months	Moderate (cones only)	2 - 4 mnth

Table S5. Key features of the donor and recipient models and the ages at time of use.

Primary antibody Target	Animal	Source	Concentration	Block	Secondary antibody (AlexaFluor)
RXRγ	Rabbit	Abcam (ab15518)	1:200	10% FBS<br>1% BSA<br>0.1% Triton	Goat anti-rabbit 546
Biotinylated Peanut agglutinin (PNA)	N/A (plant lectin)	Vector Labs (L-1070)	1:500	10% FBS<br>1% BSA<br>0.1% Triton	Streptavidin 633
S-OPSIN (Opn1sw)	Goat	Santa Cruz (sc-14365)	1:100	2% NDS<br>1% BSA<br>0.1% Triton	Donkey anti-goat 546
PERIPHERIN2	Rabbit	Gift from G.Evans	1:500	10% FBS<br>1% BSA<br>0.1% Triton	Goat anti-rabbit 546
ROD α-TRANSDUCIN	Rabbit	Santa Cruz (sc389)	1:500	2% NDS<br>1% BSA<br>0.1% Triton	Donkey anti-goat 546
CONE ARRESTIN (CARR)	Rabbit	Millipore (ab15282)	1:200	2% NDS<br>1% BSA<br>0.1% Triton	Donkey anti-rabbit 647
PROTEIN KINASE C-α	Mouse	Millipore (05-983)	1:500	10% NGS<br>1% BSA<br>0.1% Triton	Goat anti-mouse 546
GFP.FITC	Goat	Abcam (ab6662)	1:200	10% FBS<br>1% BSA<br>0.1% Triton	N/A

Table S6. Antibodies and blocking conditions used for immunohistochemistry. FBS – foetal bovine serum; BSA- Bovine Serum Albumin; NDS – Normal Donkey Serum; NGS – Normal Goat Serum

Gene	F primer (5' to 3')	R primer (5' to 3')	Amplicon (bp)	Probe number
Arr3	GCTAACCTGCC CTGTTCAGTA	TTCAAAGTCAACC CCACAGG	75	64
Chrnb4	GCTCCTCGTCTC TCTGTTCG	CATCCATCAGCTT CTCCTCTG	77	64
En2	GACCGGCCTTC TTCAGGT	CCTGTTGGTCTGA AACTCAGC	132	110
Fgfr4	AATCGTATTGG AGGCATTCG	TCCGAGGGTACCA CACTTTC	71	47
Gnat2	CATGTCCACAC TAGGCATTGAC	GCCAGGTTGTTGA GCTGTCT	72	21
Opn1mw (M-opsin)	ATCGTGCTCTGC TACCTCCA	TTTCTGTTGCTTTG CCACTG	60	5
Pde6c	CGAAGGTGAAG GTGACTGAAG	GCTGCCTCCTAAA TCTGTGG	86	4
Rxrγ	CAGAAGTGCCT GGTCATGG	CCTCACTCTCTGCT CGCTCT	82	82
Opn1sw (S-opsin)	CAACCCCATCA TCTACTGCTT	GACACGTCAGATT CGTCTGC	96	4
Thrβ2	ATGCATCTATGT TGGCATGG	GCTTGGCTAGCCT CTTGCT	62	42
Nrl	TTCTGGTTCTGAC AGTGACTACG	TGGGACTGAGCAGA GAGAGG	77	53
Nr2e3	CAGCCAGCCTG TGAGGTT	AGAAGCTCAATGC GCTCAG	81	32
Rhodopsin	ACCTGGATCAT GGCGTTG	TGCCCTCAGGGAT GTACC	70	32
β-actin	AAGGCCAACCG TGAAAAGAT	ACCAGAGGCATAC	110	56

Table S7. Forward and Reverse primer sequences used for qRT-PCR

Supplemental Experimental Procedures

Ethics statement

All animal studies were carried out under the Animals (Scientific Procedures) Act 1986 under a project license PPL 70/8120 issued by the UK Government Home Office and conducted in accordance with protocols approved by the Animal Welfare and Ethics Committee of the UCL Institute of Ophthalmology. All animals were killed by cervical dislocation performed by trained personnel (approved under Schedule 1 as a method of humane killing). All efforts were made to minimize the number and suffering of animals used in these experiments.

Animals

Male and Female mice were group housed in the animal facility in the Institute of Ophthalmology at University College London on a standard 12-hour light/dark cycle at the same light levels throughout the experimental period. Animals were kept in individually ventilated cages on animal grade wood chip and given access to nesting material and food and water ad lib.

The following lines were used: *OPN1LW*-eGFP (Fei and Hughes, 2001) (Mutant Mouse Regional Resource Centre, MMRRC); *Chrnb4-e*GFP (Siegert et al., 2009) (MMRRC; Supplemental Fig. S2A. N.B. this model is usually homozygous for the *Pde6β rd1)* mutation, which causes rapid retinal degeneration. Mice were backcrossed with *C57Bl/6* to remove this mutation from the line); *Nr2e3*^{rd7rd7} (Haider et al., 2000), *Nrl*-GFP(Akimoto et al., 2006) and *Nrl*$^{-/-}$ (Mears et al., 2001) (gifts from A. Swaroop, National Eye Institute, Bethesda, Maryland); *Crx*-GFP(Samson et al., 2009) (gift from C. Cepko, Harvard, USA) *Nr2e3*$^{rd7/rd7}$*Crx*-GFP (bred on site from the above lines; Supplemental Fig. S1B; *Nrl*$^{-/-}$*;Nrl*-GFP$^{+/+}$ (bred on site from the above lines; Supplemental Fig. S2C; *Pde6c*$^{cpfl1/cpfl1}$ (Chang et al., 2002) (gift from X-Q Ding, University of Oklahoma); *Prph2*$^{rd2/rd2}$ (cba.rds) (Sanyal et al., 1980) (gift from D. Bok, Jules Stein Institute, Los Angeles); *Cnga3*^{cpfl5} (Pang et al., 2010) (gift from X-Q Ding, University of Oklahoma); *Nrl*$^{-/-}$*;RPE*$^{R91W/R91W}$ (Samardzija et al., 2014) (gift from C. Grimm, University of Zurich, Switzerland); B6.Cg-Tg(CAG-DsRed*MST)1Nagy/J ("*DsRed*") (Jackson Labs); *Nrl*$^{-/-}$;DsRed (bred on site from above) and Wildtype *C57Bl/6J* – Harlan Laboratories. See **Supplemental Table S5** for summary of key features and ages used for all donor and host lines.

FACS isolation of donor cells

The neural retinae of donor *Chrnb4-e*GFP, *Nrl*$^{-/-}$*;Nrl*-GFP$^{+/+}$ and *Nr2e3*$^{rd7/rd7}$*;Crx*-GFP$^{+/+}$ mice were isolated by dissection. Unless otherwise stated, the donor ages used (+/- 1.5 days) were embryonic day (E) 15.5, postnatal day (P) 1 and P8. A single-cell suspension was obtained using papain-based digestion (Worthington Biochemical, Lorne Laboratories, UK) per the manufacturer's instructions. Fluorescent Activated Cell Sorting (FACS) was used to purify the GFP expressing population utilizing a special order 5-laser BD Influx™ Cell Sorter (BD Biosciences) equipped with a 200mW 488nm blue laser to excite GFP. GFP positive cells were collected using the 488-530/40nm detector. A 70µm nozzle at 50 psi was used for sorting and cells were collected into a 1:1 FBS/ EBSS solution. Sorted GFP^{+ve} cells were re-suspended at 200,000 live cells / µl (as assessed using a Scepter hand-held cell counter; Millipore, UK) in sterile EBSS and DNase (0.05%) and kept on ice prior to transplantation. N.B. Due to the very low cellular yield, *Chrnb4-e*GFP and *OPN1LW*-eGFP donor cells were prepared at a concentration of 100,000 live cells per µl.

Preparation of donor cells from mouse ESCs

A mouse EK.CCE ES cell line (Evans and Kaufman, 1981) (129/SvEv; gift of Professor E. Robertson) was maintained in GMEM containing 10% Knockout Serum Replacement (KSR), 1% Fetal Bovine Serum (FBS), 0.1mM NEAA, 1mM pyruvate, 0.1mM 2-mercaptoethanol with 2000U/ml LIF, 0.5µM MEK inhibitor (PD0325901) and 1.5 µM GSK3 inhibitor (CHR99021)(Gonzalez-Cordero et al., 2013). Briefly, for 3D retinal differentiation, $3x10^4$ dissociated ES cells were re-suspended per millilitre of differentiation medium (GMEM containing 1.5% KSR, 0.1mM NEAA, 1mM pyruvate, 0.1mM 2-mercaptoethanol), plated into 96 well low-binding plates and incubated at 37°C, 5% CO_2. Embryoid body cell aggregates (EBs) formed within 24 hrs, on day 1 of culture, growth factor reduced Matrigel™ (GIBCO) was added to each well to give a final concentration of 2%. For whole EB retinal differentiation towards photoreceptor cell fate, EBs were transferred into retinal maturation medium (DMEM/F12 Glutamax media containing N2 supplement and Pen/strep, herein Retinal Maintenance Media, RMM) at day 9, plated in low-binding plates at a density of 6 EBs/cm^2 and incubated at 37°C, 5% CO_2. The media was changed every 2-3 days, with the addition of 1mM Taurine and 500nM retinoic acid from day 14 of culture onwards. EBs were labelled with AAV ShH10.L/MOpsin.GFP

(Kruczek et al., 2017) on day 20 of culture and harvested on d26-30. This encompasses 3 changes of media between viral administration and cell preparation.

Note that the time-course of differentiation can be heterogeneous both within a given EB and between EBs even in the same dish. This may account for the higher inter-animal variation between transplantations using mESCs-derived cones than between donor-derived cones, which tend to be more developmentally synchronized.

FACS isolation of mouse ESC-derived photoreceptors

For transplantation, EBs were dissociated at day 26-30 of culture into a single cell suspension using a modified protocol using reagents from a papain-based Neurosphere Dissociation Kit (Miltenyi Biotec, 130-095-943). Briefly, samples were incubated in a papain-based enzyme dissociation mix (Miltenyl Biotech) at 37°C for 15 min, gently triturated and then spun down at 320g for 7 minutes at room temperature. Cell pellets were re-suspended in HBSS (with 0.5mM MgCl2) with FCS (1%), 66% 25mM HEPES and DNase solution (1%) and passed through a 35 micron cell strainer at a concentration of ~20 million cells/mL. Cells were FACS sorted for GFP fluorescence using a special order 5-laser BD Influx Cell Sorter^{TM} (BD Biosciences) equipped with a 200mW 488nm blue laser to excite GFP. GFP positive cells were collected using a 488-530/40nm detector. A 70μm nozzle at 50 psi was used and cells were collected into a 20% FBS in RMM solution. Sorted GFP^{+ve} cells were re-suspended at 200, 000 live cells / μL in sterile HBSS (+Ca^{2+}, Mg^{2+}) and DNase solution (3%) and kept on ice prior to injection. The final supernatant was also retained for injection as a control for viral carry-over (Pearson et al., 2016, West et al., 2012, Gonzalez-Cordero et al., 2013).

Donor cell transplantation

Donor cells were introduced into the subretinal space of the superior (dorsal) retina by a single injection of 1μl. Injection protocols were as described in previous publications (Pearson et al., 2016, Pearson et al., 2012). Briefly, intraperitoneal injections of an anaesthetic mixture containing Dormitor, ketamine and sterile water in a 5:4:42 ratio were used before injections (0.01 ml per gram). Pupils were dilated using 1% topical tropicamide. 1μl injections were made slowly by a single surgeon with a sterile Hamilton syringe at an oblique angle through the superior sclera into the subretinal space to produce visible retinal detachment in the superior retina.

Introducing a small parathentesis in the anterior chamber prior to cell injection relieved the associated increases in intraocular pressure. Both eyes received topical treatment of chloramphenicol and viscotears. Anaesthesia was reversed using intraperitoneal injections of 0.1 mg/ml of Antisedan (Pfizer, Kent UK).

Recipients of transplanted cells were typically sacrificed between 2 and 3 wks post injection and the eyes removed and placed in 1% paraformaldehyde (PFA) and the corneas and lens carefully removed. Eyes were kept in 1% PFA for 20 – 30 minutes before being washed in PBS, cryoprotected with 20% sucrose in PBS solution and cryo-embedded in OCT (Tissue-Tek). Cryosections 18 μm thick were cut in the coronal plane. All sections were collected for analysis.

Analysis of cell counts in transplanted eyes by fluorescence microscopy

After immunostaining and enhancing GFP signal with 1:200 FITC conjugated anti-GFP goat polyclonal antibody (Abcam, UK), sections from injected eyes were viewed with a fluorescence microscope (Observer Z.1, Zeiss). Every third cryosection was viewed and all GFP+ cells with cell bodies located in the ONL were counted and assessed, when possible, for co-staining and morphology. Cells were counted if the whole cell body was correctly located within the ONL and at least one of the following was visible; spherule synapse, inner/outer processes, inner/outer-segments. The total number of cells for each injected eye was calculated as three times this count. Where relevant, counts were made in a blinded fashion, whereby the assessor was unaware of the donor cell source/recipient genotype for any given eye. Cell counts for individual eyes were excluded from the analysis: if donor cells were present in the vitreous, indicative of accidental intravitreal transplantation of the cells; if there was no cell mass present in the subretinal space, indicative of reflux at time of injection; and/or there was significant macrophage infiltration and evidence of level II/III rejection, as defined in (West et al., 2010) (but see Results for details regarding specific models).

Analysis of cell counts in transplanted eyes by flow cytometry

Transplanted retinae were taken 2-3 wks post-transplantation. Neural retinae were isolated by dissection and any overlying cell mass carefully removed using direct visualisation under a fluorescence microscope. Individual neural retinae were dissociated using the papain-based Neurosphere Dissociation Kit, described above. An aliquot from each sample was taken to determine absolute cell counts using the Beckman Coulter

Vi-Cell XR Cell Viability Analyser (Beckman Coulter). The remaining media was spun down at 320g for 7 minutes at 4°C, aspirated and then re-suspended in 350 μL of $DMEM^+$ media with 1.5 μM of SYTOX™ Blue Dead Cell Stain (ThermoFischer Scientific) for sample acquisition on the flow cytometer. All samples were acquired using a BD LSRFortessa™ X-20 flow cytometer (BD Biosciences), equipped with 5 lasers (i.e. 355nm, 405nm, 488nnm, 561nm & 640nm lasers). The entire sample was acquired for analysis due to the low total frequency of transplanted donor cells. Fluorescence gates were defined by using *NrlGFP* and *DsRed* (or *$Nrl^{-/-}$;DsRed*, where appropriate), and wild type retinae as positive and negative controls, respectively. Cellular analysis of the samples included a gating hierarchy whereby dead cells, cellular debris and cellular aggregates were excluded prior to the analysis of single, live cells, as shown in **Supplemental Fig. S3B-G**. It is important to note that while every effort is made to remove any remaining donor cell mass prior to dissociation, it is not possible to be completely certain of this. Therefore, it is possible that some GFP^+-only cells reflect donor cells within the SRS. We suggest that for these reasons, this method of assessment provides positive demonstration of CMT but does not provide an accurate quantification of the number of integration events. In this regard, FISH analysis is likely to be more accurate.

Immunohistochemistry and Histology

Eyes were dissected out after administering a small burn to the overlying sclera to provide a landmark for the superior retina. The eye cups were then carefully orientated and embedded in a standardized fashion in OCT (TissueTek) before being left overnight (o/n) at -20°C and then cut as transverse sections 18 μm thick. To avoid oblique cuts, all images shown are from the central most region of the eye, immediately adjacent to or through the optic nerve. Where comparing across developmental time points, immunohistochemistry was performed at the same time for all time points for any given marker. Specific details for each antibody and protocols used for immunohistochemistry can be found in **Supplemental Table S6**. Cryosections were air-dried for 15 - 30 min and *washed* in phosphate-buffered saline (PBS). Sections were pre-blocked for 1 hr at room temperature (RT) in a blocking solution before being incubated with appropriate primary antibody o/n at 4°C. After rinsing with PBS, sections were incubated with secondary antibody for 2 hrs at RT, rinsed and counter-stained with Hoechst 33342. Negative controls omitted the primary antibody. Note that all images showing GFP^+ cells have been counter-stained with an anti-GFP antibody.

Fluorescent In situ hybridization

Eye-cups were fixed for 1 h in 4% PFA and cryopreserved in OCT. Serial sections were cut (Note 10 μm thick, compared to 18 μm elsewhere) across 6 sets of slides and screened for GFP^+ cells. For Fluorescent In Situ Hybridization (FISH) treated with 0.2M HCl for 20 minutes, at RT, and 5 minutes wash in 2x SSC/0.05 Tween20. Followed by 2x SSC incubation 20 minutes, at 80°C. Enzyme digestion as preformed with 0.1mg/mL of Protease K solution for 10 minutes, at 37°C. After washed and dehydrated, slides were left to air dry. 10μL of mouse Y chromosome paint probe (Empire Genomics) was added to each 22x22 mm area and sealed. Following denaturation, for 10 minutes at 90°C, sections were incubated overnight at 42°C. After seal removal slides were placed in 2x SSC for 10 minutes at 42°C. After the PBS wash, slides were blocked in PSB/5%BSA/0.1%triton for 30 minutes at room temperature followed by overnight incubation, at 4°C, with anti-GFP FITC conjugated antibody in 1:100 dilution. After the PBS wash, slides were counterstained with DAPI (10 μM).

Although we were able to obtain some examples of GFP^+ signal in sections also processed for FISH (shown in **Fig. 5**), it was not possible to retain the GFP signal with sufficient reliability to obtain cell counts. To enable assessment of the number and morphology of GFP^+ cells and the number of Y-chromosome positive (Y^+) cells in each retina, serial sections were processed for either GFP or FISH. Blinded counts were made across the entire section and multiplied to give an estimate per eye. The total number of Y-chromosome+ nuclei was divided by the total number of GFP^+ cells to give a percentage of integration events versus CMT. Note that Y-chromosome staining was robust with ~85% of nuclei labelling with the Y-probe in control male retinal sections. However, since labelling was not 100%, this method of quantification is likely to yield a slight underestimate of the percentage of the GFP^+ cells that are additionally Y^+.

Image acquisition

Retinal sections were viewed on a confocal microscope (Leica TCS SPE, Leica Microsystems), typically using x40 oil-immersion objective. Hoechst 33342 was excited using a 400nm, GFP using a 488nm and Alexa546/DsRed using 543nm laser lines. Collection of the emission wavelengths were set according to Leica suggested pre-sets, adjusted to ensure minimal overlap of collected emission spectra. Series of XY optical sections (<1 μm thick) were taken at 1.0μm steps throughout the depth of the section and built into a stack to

give xyz projection image. LAS-AF image software (Leica) was used. Individual xy images were acquired using a 2-frame average and at 1024 x 1024 resolution.

Semi-thin sections and Electron Microscopy

Mice were sacrificed and the eyes removed after administering a small burn to the overlying sclera to provide a landmark for the superior retina. Eyes were fixed in 3% glutaraldehyde / 1% PFA buffered to pH 7.4 with 0.08 sodium cacodylate-HCl. The cornea and lens were removed and the eye-cups orientated and processed, as previously described (Pearson et al., 2012). Briefly, following a washing step (15 min; 2.5% glutaraldehyde and 0.1 m cacodylate buffered to pH 7.4), the eyes were osmicated for 2.5 hrs in a 1% aqueous solution of osmium tetroxide in the dark, followed by dehydration steps through ascending ethanol series (50 - 100%, 10 min per step with rotation). After three changes of 100% ethanol, specimens were passed through propylene oxide (3 x 10 min) and left in a 50:50 mixture of propylene oxide and araldite for a minimum of 3 hrs with rotation at RT. Following a single change to fresh araldite (5 hrs with rotation) the specimens were embedded and cured for 48 hrs at 60°C. Semi-thin (0.7 μm) and ultra-thin (0.07 μm) sections were cut using a Leica Ultracut S microtome fitted with an appropriate diamond knife (Diatome histoknife Jumbo or Diatome Ultra-thin respectively). Ultra-thin sections were collected on copper grids (100 mesh, Agar Scientific, UK), contrast-stained with 1 % uranyl acetate and lead citrate and analysed using a JEOL 1010 Transmission Electron Microscope (80 kV), fitted with a digital camera for image capture. Semi-thin sections were stained with 1% toluidine blue and evaluated using a Leitz Diaplan microscope fitted with a Leica digital camera DC 500 for image capture.

Real-time and RT-PCR analysis

RNA was extracted with RNeasy Micro/Mini Kit (QIAGEN) and reverse-transcribed using QuantiTect Reverse Transcription Kit (QIAGEN). The cDNA was amplified with gene-specific primers (see **Supplemental Table S7**). PCRs were conducted using at least 3 separate RNA preparations. Real-time quantitative RT-PCR was performed with a thermal cycler (7900HT; Applied Biosciences), as previously described. Reagents were obtained from Roche Diagnostics and primers were designed for specific probe-binding regions using the Roche Universal Probe Library. Samples were run in duplicate and at least 3 independent differentiation cultures were analysed.

Statistics and randomisation

All means are stated ± standard deviation, except cell counts which are stated as ± standard error of the mean. N = number of eyes examined, n = number of cells, where appropriate. For qualitative histological assessments, at least 3 eyes from independent animals per group were used. For cell counts, both eyes per animal received injections to reduce the total number of animals used but the order of animals (different models) was randomized to ensure distribution between beginning and end of any given injection session. Cell counts represent data from at least three independent donor cell preparation/transplantation sessions, wherever possible. Eyes presenting with evidence of moderate inflammation and signs of acute rejection (Grade 2/3, as described by (West et al., 2010, Warre-Cornish et al., 2013)) were omitted from the cell counts but the numbers are shown as a proportion of the total number injected e.g. N = X successful transplants meeting minimum acceptance criteria/Y total number of eyes injected.

Statistical significance was assessed using a one-way ANOVA test with Dunnett's (comparing against wild-type) or Tukey's or Bonferroni's (inter-group comparisons) correction applied for multiple comparisons, as appropriate. P values are presented as $p < 0.05$ = *, $p < 0.01$ = ** and $p < 0.001$ = ***.

Data Availability

The data sets generated during and/or analysed during the current study are available from the corresponding author on reasonable request

References for Supplemental Information

AKIMOTO, M., CHENG, H., ZHU, D., BRZEZINSKI, J. A., KHANNA, R., FILIPPOVA, E., OH, E. C., JING, Y., LINARES, J. L., BROOKS, M., ZAREPARSI, S., MEARS, A. J., HERO, A., GLASER, T. & SWAROOP, A. 2006. Targeting of GFP to newborn rods by Nrl promoter and temporal expression profiling of flow-sorted photoreceptors. *Proc Natl Acad Sci U S A,* 103**,** 3890-5.

CHANG, B., HAWES, N. L., HURD, R. E., DAVISSON, M. T., NUSINOWITZ, S. & HECKENLIVELY, J. R. 2002. Retinal degeneration mutants in the mouse. *Vision Res,* 42**,** 517-25.

FEI, Y. & HUGHES, T. E. 2001. Transgenic expression of the jellyfish green fluorescent protein in the cone photoreceptors of the mouse. *Vis Neurosci,* 18**,** 615-23.

HAIDER, N. B., JACOBSON, S. G., CIDECIYAN, A. V., SWIDERSKI, R., STREB, L. M., SEARBY, C., BECK, G., HOCKEY, R., HANNA, D. B., GORMAN, S., DUHL, D., CARMI, R., BENNETT, J., WELEBER, R. G., FISHMAN, G. A., WRIGHT, A. F., STONE, E. M. & SHEFFIELD, V. C. 2000. Mutation of a nuclear receptor gene, NR2E3, causes enhanced S cone syndrome, a disorder of retinal cell fate. *Nat Genet,* 24**,** 127-31.

MEARS, A. J., KONDO, M., SWAIN, P. K., TAKADA, Y., BUSH, R. A., SAUNDERS, T. L., SIEVING, P. A. & SWAROOP, A. 2001. Nrl is required for rod photoreceptor development. *Nat Genet,* 29**,** 447-52.

PANG, J. J., ALEXANDER, J., LEI, B., DENG, W., ZHANG, K., LI, Q., CHANG, B. & HAUSWIRTH, W. W. 2010. Achromatopsia as a potential candidate for gene therapy. *Adv Exp Med Biol,* 664**,** 639-46.

SAMSON, M., EMERSON, M. M. & CEPKO, C. L. 2009. Robust marking of photoreceptor cells and pinealocytes with several reporters under control of the Crx gene. *Dev Dyn,* 238**,** 3218-25.

SANYAL, S., DE RUITER, A. & HAWKINS, R. K. 1980. Development and degeneration of retina in rds mutant mice: light microscopy. *J Comp Neurol,* 194**,** 193-207.

SIEGERT, S., SCHERF, B. G., DEL PUNTA, K., DIDKOVSKY, N., HEINTZ, N. & ROSKA, B. 2009. Genetic address book for retinal cell types. *Nat Neurosci,* 12**,** 1197-204.

WEST, E. L., GONZALEZ-CORDERO, A., HIPPERT, C., OSAKADA, F., MARTINEZ-BARBERA, J. P., PEARSON, R. A., SOWDEN, J. C., TAKAHASHI, M. & ALI, R. R. 2012. Defining the integration capacity of embryonic stem cell-derived photoreceptor precursors. *Stem Cells,* 30**,** 1424-35.
