# Supplementary material for: Transplanted Donor- or Stem Cell-Derived Cone Photoreceptors Can Both Integrate and Undergo Material Transfer in an Environment-Dependent Manner
Source: Stem Cell Reports. 2018 Jan 4;10(2):406–21. doi: 10.1016/j.stemcr.2017.12.008 (PMC5830910; doi:10.1016/j.stemcr.2017.12.008)
Supplement: Document S2. Article plus Supplemental Information [file mmc2.pdf]

Stem Cell Reports
Article

OPEN ACCESS

Transplanted Donor- or Stem Cell-Derived Cone Photoreceptors Can Both Integrate and Undergo Material Transfer in an Environment-Dependent Manner

Paul V. Waldron,[1] Fabiana Di Marco,[2,3] Kamil Kruczek,[1,3] Joana Ribeiro,[1,3] Anna B. Graca,[1] Claire Hippert,[1] Nozie D. Aghaizu,[1] Aikaterini A. Kalargyrou,[1] Amanda C. Barber,[1,4] Giulia Grimaldi,[2,5] Yanai Duran,[1] Samuel J.I. Blackford,[1] Magdalena Kloc,[1] Debbie Goh,[1] Eduardo Zabala Aldunate,[2] Robert D. Sampson,[1] James W.B. Bainbridge,[1] Alexander J. Smith,[1] Anai Gonzalez-Cordero,[1] Jane C. Sowden,[2] Robin R. Ali,[1] and Rachael A. Pearson[1,*]
[1]UCL Institute of Ophthalmology, 11–43 Bath Street, London EC1V 9EL, UK
[2]Stem Cells and Regenerative Medicine Section, UCL Great Ormond Street Institute of Child Health, University College London, 30 Guilford Street, London WC1N 1EH, UK
[3]These authors contributed equally
[4]Present address: John van Geest Centre for Brain Repair, Robinson Way, Cambridge CB2 0PY, UK
[5]Present address: Institute of Basic Medical Sciences, University of Oslo, Oslo, Norway
*Correspondence: rachael.pearson@ucl.ac.uk
https://doi.org/10.1016/j.stemcr.2017.12.008

SUMMARY

Human vision relies heavily upon cone photoreceptors, and their loss results in permanent visual impairment. Transplantation of healthy photoreceptors can restore visual function in models of inherited blindness, a process previously understood to arise by donor cell integration within the host retina. However, we and others recently demonstrated that donor rod photoreceptors engage in material transfer with host photoreceptors, leading to the host cells acquiring proteins otherwise expressed only by donor cells. We sought to determine whether stem cell- and donor-derived cones undergo integration and/or material transfer. We find that material transfer accounts for a significant proportion of rescued cells following cone transplantation into non-degenerative hosts. Strikingly, however, substantial numbers of cones integrated into the $Nrl^{-/-}$ and $Prph2^{rd2/rd2}$, but not $Nrl^{-/-};RPE65^{R91W/R91W}$, murine models of retinal degeneration. This confirms the occurrence of photoreceptor integration in certain models of retinal degeneration and demonstrates the importance of the host environment in determining transplantation outcome.

INTRODUCTION

Loss of vision due to photoreceptor degeneration is a leading cause of blindness in the developed world, and replacing lost photoreceptors by the transplantation of healthy cells represents a promising therapeutic strategy. We, and others, have previously reported the effective transplantation of post-mitotic rod precursors either isolated from developing retinas or derived from murine or human pluripotent stem cells (PSCs) (MacLaren et al., 2006; Bartsch et al., 2008; Lakowski et al., 2010; Pearson et al., 2012; Gonzalez-Cordero et al., 2013). When transplanted into murine models of retinal disease, and if present in sufficiently large numbers, these cells have been shown to improve various measures of visual function (Barnea-Cramer et al., 2016; Singh et al., 2013; Barber et al., 2013; MacLaren et al., 2006; Pearson et al., 2012). Together, these findings demonstrate that transplanted donor rod photoreceptor cells have the potential to restore vision.

Human vision relies heavily on cone photoreceptors, and diseases that lead to their loss, such as age-related macular degeneration (AMD), are particularly devastating. We previously provided the first report of cone transplantation (Lakowski et al., 2010) using a *Crx*-GFP transgenic line that labels rod and cone photoreceptors. We transplanted embryonic *Crx*-GFP^{+} donors at a stage when the majority was committed to a cone fate. While large numbers of GFP-labeled photoreceptors were found in the host outer nuclear layer (ONL), many resembled rods in their morphology. The mixed nature of the *Crx*-GFP^{+} donor population presented the question of whether the preponderance of rod-like cells was due to plasticity in the fate of the donor photoreceptors (Siegert et al., 2012) or the result of more successful integration of the rod precursors present within the mixed population.

NRL and NR2E3 act together with CRX to activate rod-specific genes and suppress cone gene expression. Rod differentiation is thus impaired in *Nrl* and *Nr2e3* deficient retinas; the $Nr2e3^{rd7/rd7}$ mouse has increased numbers of S-opsin^{+} cone-like photoreceptors, while in the $Nrl^{-/-}$ mouse, all photoreceptors fated to become rods instead acquire a cone-like (so-called "cod") hybrid phenotype. In keeping with the idea that photoreceptors might retain plasticity after terminal mitosis, Ader and colleagues (Santos-Ferreira et al., 2015) noted that following transplantation of postnatally derived

This is an open access article under the CC BY license (http://creativecommons.org/licenses/by/4.0/).

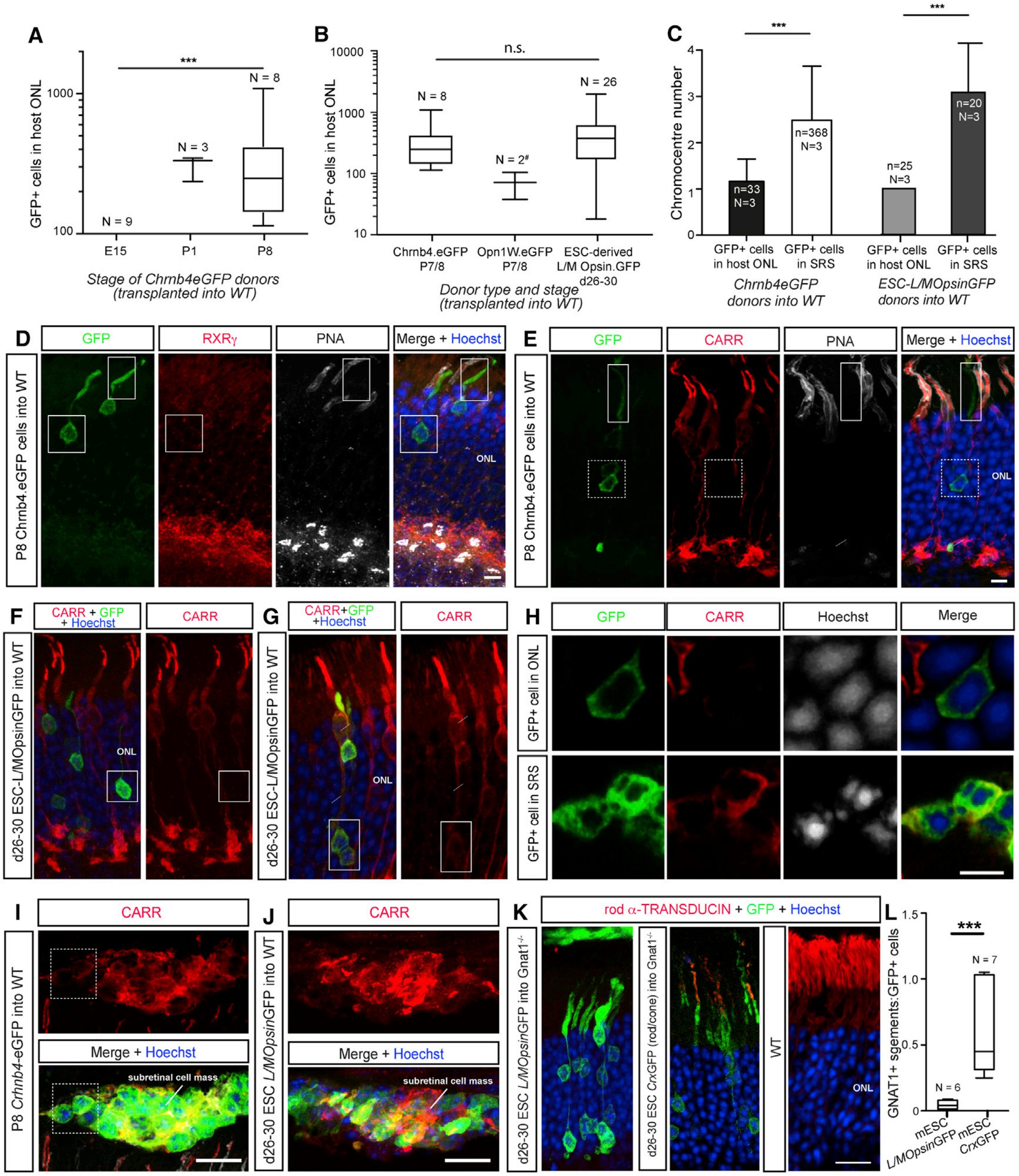

Figure 1. Transplantation of Donor- and Stem Cell-Derived Cone Precursors Leads to the Presence of Rod-like Cells within the Host ONL

(A and B) Box-and-whiskers (SD) plot of number of GFP^+ cells within wild-type (WT) host ONL after transplantation of (A) *Chrnb4*-$EGFP^+$ cells from different stages of development and (B) donor-derived *OPN1LW*-$EGFP^+$ and mESC-derived *L/MOpsin*-GFP^+ cone precursors, compared with donor-derived *Chrnb4*-$EGFP^+$ cells at an equivalent stage of development (~P7/8).

(C) Histogram of mean number of chromocenters/nucleus (mean + SD) of GFP^+ cells within the host ONL and in the subretinal space (SRS) following transplantation of P7/8 *Chrnb4*-$EGFP^+$ and day 26–30 mESC-derived *L/MOpsin*-GFP^+ donors.

(D–G) IHC shows that, regardless of donor origin, most GFP^+ cells within host ONL are rod-like in morphology and display a heterogeneous expression profile with respect to cone markers (compare F and G).

(H–J) GFP^+ cells within the host ONL typically have single chromocenter nuclei (H), while GFP^+ donor cells in the SRS have nuclei with multiple chromocenters and (I) and (J) express cone markers.

(legend continued on next page)

Nrl$^{-/-}$GFP$^+$ cone-like cells, GFP$^+$ cells within the retina of *Pde6c*$^{cpfl1/cpfl1}$ model of cone degeneration bore rod-like morphological features, including small spherule synapses and elongated outer segments. Strikingly, though, these cells also expressed cone arrestin (CARR) and S-OPSIN and appeared capable of driving responses to photopic stimuli (Santos-Ferreira et al., 2015). Another recent study by Wallace and colleagues (Smiley et al., 2016) described the transplantation of *Nrl*$^{-/-}$ cells and those derived from a novel cone-GFP reporter mouse line (*Ccdc136*-GFP). Similarly, apparently integrated donor cells exhibited morphologies more typical of rods than cones, but in this study cone marker expression was not observed.

During our own investigations into photoreceptor transplantation, we made observations that led us to question the underlying cellular mechanisms behind functional rescue following donor photoreceptor transplantation (Pearson et al., 2016). While donor rod photoreceptor migration and integration occurs, it accounts for far fewer of the reporter-labeled cells observed than previously thought; post-mitotic rod precursors can also undergo a process of material transfer with photoreceptors within the recipient retina (Pearson et al., 2016) (see also Ortin-Martinez et al., 2017; Singh et al., 2016; Decembrini et al., 2017; Santos-Ferreira et al., 2016). The cellular mechanisms by which this occurs have yet to be determined but they do not appear to involve permanent donor-host nuclear or cell fusion, or the uptake of free protein or nucleic acid from the extracellular environment. Instead, it appears that a wide array of either RNAs and/or proteins might be exchanged between stage-specific donor rod precursors and adult host photoreceptors *in vivo* (Pearson et al., 2016), apparently in quantities sufficient to render the recipient cells functional.

Here, we sought to determine whether purified cone photoreceptors, derived either from donor retinas or from embryonic stem cell (ESC)-derived retinas, undergo cell integration and/or engage in material transfer with host photoreceptors after transplantation into different models of retinal degeneration. Specifically, we sought to determine whether the host environment influenced the relative contributions of these two mechanisms to transplantation outcome.

RESULTS

Transplantation of Donor- and Stem Cell-Derived Cone Precursors into Wild-Type Recipient Results in GFP$^+$ Cells within Host ONL with Rod-like Morphologies

We first assessed the outcomes of transplantation of cone photoreceptors isolated from a variety of donor- and stem cell-derived sources. To transplant purified populations of cone precursors at different stages of development, we used the *Chrnb4*-EGFP (Figures S1A and S2A) and *OPN1LW*-EGFP cone reporter mouse lines to isolate early- and late-stage cone precursors, respectively. *L/MOpsin*-GFP reporter virally labeled cones were additionally derived from murine ESC (mESC) retinal organoid cultures, as we have described previously (Kruczek et al., 2017; Gonzalez-Cordero et al., 2013). Previously we, and others, have found rod photoreceptor transplantation outcome to be significantly affected by the developmental stage of the donor cell at the time of transplantation. Therefore, GFP$^+$ cone precursors were isolated at various stages of development: embryonic day 15 (E15) and post-natal day 1 (P1) (peaks of cone and rod birth [Young, 1985]), and P8 (stage most effective for rod transplantation [Pearson et al., 2012]).

In the developing *Chrnb4*-EGFP retina, GFP expression was heterogeneous but a population of brightly fluorescent GFP$^+$ cells ($[GFP]_{high}$) could be readily isolated at P1, P8, and adult stages by fluorescence-activated cell sorting (FACS) that comprised cone precursors (Figures S1A, S1D, S1E, S2A, and S2B; Tables S1 and S2). At E15, it was not possible to isolate sufficient numbers of $[GFP]_{high}$ cells so, for this age, all GFP$^+$ cells were collected. In each case, purified GFP$^+$ cells were transplanted into adult wild-type recipients and assessed 2–3 weeks after transplantation. In contrast to rod transplantation (Pearson et al., 2012), we observed a high transplantation failure rate (N = 9 successful transplants/20 total transplanted eyes). Donor cell masses, usually an indicator of successful transplantation, were frequently absent from the subretinal space (SRS), yet there was little evidence of acute rejection (see Warre-Cornish et al., 2014; West et al., 2010). In those transplants meeting the criteria (see Supplemental Experimental Procedures), a small number of GFP$^+$ cells were seen in the host ONL. Similar numbers were seen using P1 (n = 306 ± 34; N = 3/6) and P8 (352 ± 112; N = 8/14) donors (Figure 1A). This is lower than that

(K) Confocal images showing rod α-transducin staining in *Gnat1*$^{-/-}$ host retina after transplantation of mESC-derived *L/MOpsin*-GFP (cone) or mESC-derived *Crx*-GFP (rod and cone) donor cells.

(L) Box-and-whiskers (SD) plot showing quantification of the number of ONL-located GFP$^+$ cells expressing rod α-transducin. ANOVA with correction for multiple comparisons.

Cells in (H) show regions of interest depicted in (E) and (I), respectively. ***$p < 0.001$ (# indicates statistical tests not applied to *OPN1LW*-EGFP dataset due to low N). N, number of eyes; n, number of cells. n.s., not significant; CARR, cone arrestin; PNA, peanut agglutinin. Scale bars, 10 μm.

reported previously following the transplantation of *Nrl*-GFP$^+$ rod photoreceptors, where thousands of GFP$^+$ cells can be found in the wild-type host ONL (Warre-Cornish et al., 2014; Pearson et al., 2012). Transplanted populations of purified *OPN1LW*-EGFP$^+$ donor cells yielded similarly small numbers of GFP$^+$ cells within the recipient ONL (72 ± 47 cells; N = 2/6; Figure 1B), although loss of the line prevented further investigation.

To date, reports of cone transplantation have focused on donor-derived cells. We therefore sought to examine the behavior of stem cell-derived cones. mESC-derived retinal organoids were differentiated and transduced with a viral vector (ShH10.*L/MOpsin*.GFP) to label L/M cones, which could be purified by FACS, as previously described (Kruczek et al., 2017; Gonzalez-Cordero et al., 2013). *L/MOpsin*-GFP$^+$ cells were taken from days 26–30 of differentiation, equivalent to ~ P6–P8 (Kruczek et al., 2017), and transplanted into adult wild-type recipients. Similar numbers of GFP$^+$ cells were found in the host wild-type ONL (466 ± 86 GFP$^+$ cells; N = 26/26) (Figure 1B) as those seen following transplants of *Chrnb4*-EGFP$^+$ or *OPN1LW*-EGFP$^+$ donor-derived cones.

Transplantation of mESC- and donor-derived cones into the SRS both yielded the presence of GFP$^+$ cells within the wild-type host ONL. However, these cells presented with morphologies more typical of rods, including some or all of: rounded cell bodies distributed throughout the ONL, rather than at the apical margin like mature cones (Figures 1D–1G and S2C–S2E); round spherule-like synapses; long segments; and highly condensed nuclei with a single chromocenter (Figures 1C, 1H, S2C, and S2D). Immunohistochemistry (IHC) showed that these cells typically did not express cone markers (Figures 1D–1F), although some examples were seen (e.g., Figure 1G). Conversely, the mass of injected donor cells that remained in the SRS had cone-like nuclei with multiple chromocenters (Figures 1C, 1H, and S2E) and many expressed the cone marker CARR (Figures 1H and 1I), consistent with the expression profile of the donor cells *in vivo* (Figure S1A) and by mESC-derived L/MOpsinGFP$^+$ cells *in vitro* (Kruczek et al., 2017).

Given that cone precursors continue to express robust levels of rod-specific genes for many days after terminal mitosis (Table S2), we considered the possibility that the rod-like GFP$^+$ cells located within the host ONL might co-express rod markers and represent a hybrid state. Co-staining for rod markers was attempted, but the very high levels of expression by neighboring wild-type host rods prevented us from making assessments of co-localization with any certainty. We therefore transplanted d26-29 *L/MOpsin*GFP$^+$ mESC-derived cone precursors into the *Gnat1*$^{-/-}$ (rod α-transducin knockout) mouse model, in which rods are non-functional but do not degenerate. Despite their rod-like appearance and condensed nuclei (Figure S2F), rod α-TRANSDUCIN expression was typically absent after transplantation of mESC-derived *L/MOpsin*-GFP$^+$ cones (Figures 1K and 1L). The rare rod α-transducin$^+$, GFP$^+$ events seen most likely reflect inclusion of occasional rods in the transplanted donor population after FACS. Conversely, rod α-TRANSDUCIN was co-expressed by most GFP$^+$ cells within the host ONL following transplantation of *Crx*-GFP$^+$ ESC-derived (predominantly rod) photoreceptors (Figures 1K and 1L), as reported previously for *Nrl*-GFP$^+$ (rod) donor-derived photoreceptors (Pearson et al., 2016).

Transplantation of Cone-like Photoreceptor Precursors into Wild-Type Recipients

Given the preponderance of rod-like morphologies seen following transplantation of purified cones, we sought to genetically restrict the donor cell population's potential by deletion of key rod differentiation genes (*Nrl* and *Nr2e3*). By crossing *Nrl*$^{-/-}$ mice with *Nrl*-GFP mice, all cone-like cells express GFP, albeit at a lower level than in *Nrl*-GFP (Figures S1B, S1D, and S1E). In the *Nr2e3*$^{rd7/rd7}$ retina, early-born immature rods switch fate to become true S cones and late-born rods become "cone-like." By crossing *Nr2e3*$^{rd7/rd7}$ mice with *Crx*-GFP mice, both true cones and cone-like cells carry the GFP label. Interestingly, IHC and qRT-PCR for cone markers demonstrated a hybrid status of the genetically engineered GFP$^+$ photoreceptors from these two crosses (Figure S1; Tables S3 and S4). For example, RXRγ was widespread in the vast majority of GFP$^+$ cells in both lines, but CARR and THRβ2 were not markedly higher than in wild-type mice.

Transplantation of either *Nrl*$^{-/-}$;*Nrl*-GFP$^+$ (Figures 2A and 2C–2F) or *Nr2e3*$^{rd7/rd7}$;*Crx*-GFP$^+$ (Figures 2B and 2G) resulted in the presence of GFP$^+$ cells in the wild-type adult host ONL. Significantly higher numbers of GFP$^+$ cells were seen using post-natal, compared with embryonic (Figures 2A and 2B), donors as reported previously for rod and cone/rod populations (MacLaren et al., 2006; Lakowski et al., 2010; Gonzalez-Cordero et al., 2013) and the pure cone populations described above. There was no significant difference in outcome between the two different types of donor cell, but both yielded much lower numbers of ONL-located GFP$^+$ cells than seen previously using the respective rod-only (*Nrl*-GFP) (Pearson et al., 2012) and cone/rod (*Crx*-GFP) (Lakowski et al., 2010) donor cell controls. We used IHC to examine the identity of the GFP$^+$ cells. Again, most GFP$^+$ cells within the host ONL had a morphological appearance consistent with rod photoreceptors (Figures 2C–2G). Often, they had condensed rod-like nuclei (Figures 2C′, 2C″, and S2F) and typically did not express RXRγ (Figure 2E). Rare examples of cone-like

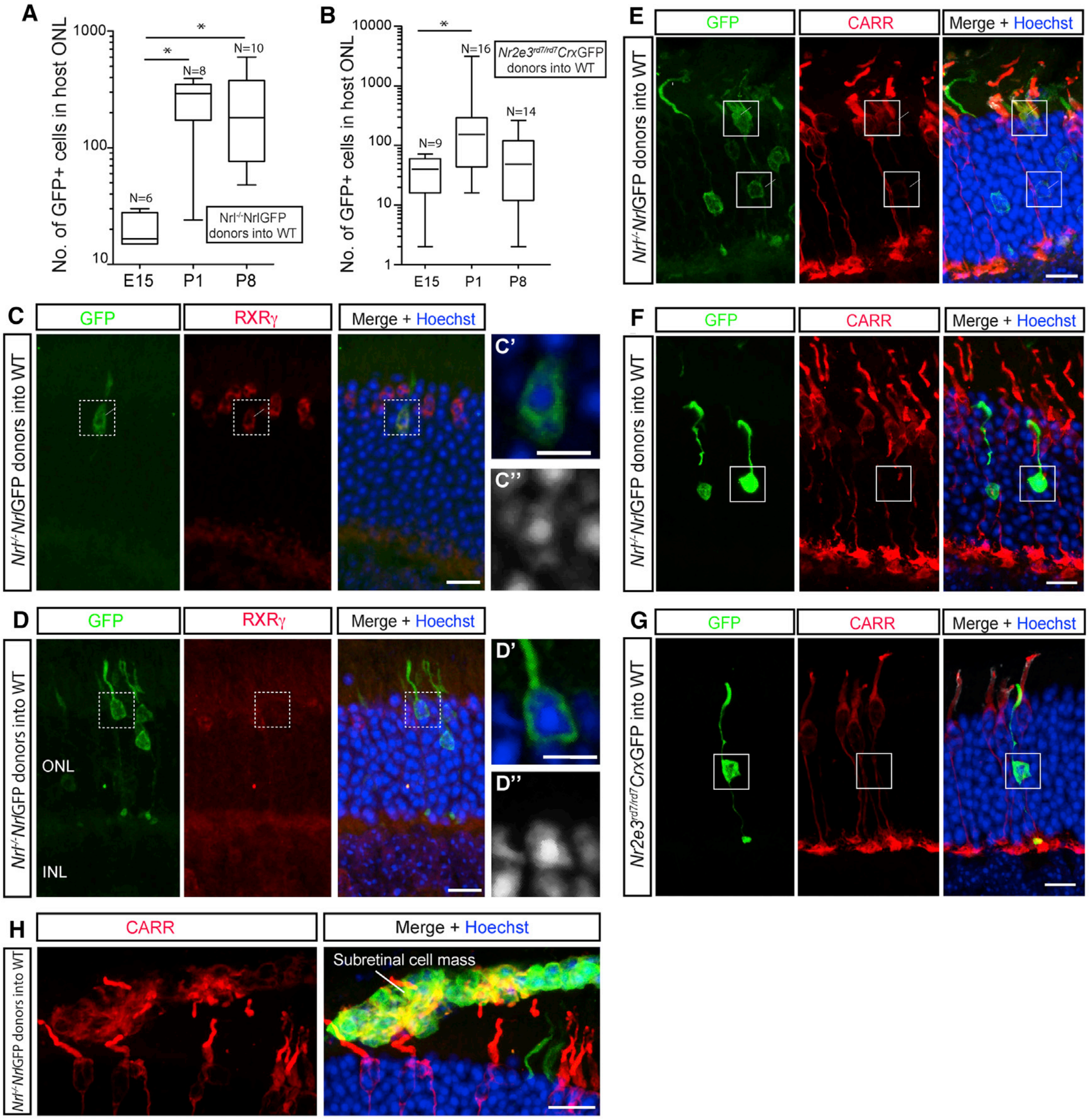

Figure 2. Transplantation of Genetically Engineered Cone-like Cells Leads to the Presence of Rod-like Cells within the Host ONL

(A and B) Box-and-whiskers (SD) plot showing the effect of donor cells age on the number of GFP$^+$ cells found within wild-type host retina after transplantation of (A) *Nrl*$^{-/-}$;*Nrl*-GFP or (B) *Nr2e3*$^{rd7/rd7}$;*Crx-GFP*. ANOVA with correction for multiple comparisons; *$p < 0.05$. N, number of eyes.

(C–H) Confocal images showing heterogeneous expression profile with respect to cone markers including (C) RXRγ$^+$ and (D) RXRγ$^-$ cells, and (E) CARR$^+$ and (F–H) CARR$^-$ cells in wild-type host ONL after transplantation of *Nrl*$^{-/-}$;*Nrl*-GFP donors (C–F) and *Nr2e3*$^{rd7/rd7}$;*Crx*-GFP donors (G). Cells in (C′, C″) and (D′, D″) show regions of interest depicted in (C) and (D), respectively. Scale bars, 10 μm.

cells were seen (<10 cells per eye); these additionally had nuclei more typical of cones and were RXRγ$^+$ (Figure 2D). Other cone photoreceptor markers were expressed in a heterogeneous manner with some (Figure 2E), but not all (Figures 2F and 2G), expressing cone markers. In keeping with the heterogeneous expression of cone markers in the donor mouse lines (Figures S1B and S1C), GFP$^+$ donor cells from *Nrl*$^{-/-}$;*Nrl*-GFP and *Nr2e3*$^{rd7/rd7}$;*Crx*-GFP donors that remained within the SRS displayed a mixed expression profile, with some but not all expressing CARR (e.g., Figure 2H).

Together, these data confirm that regardless of origin, mESC- and donor-derived cones, as well as photoreceptors genetically restricted from becoming rods, behave in a similar manner following their transplantation into the intact wild-type retina: transplantation resulted in the

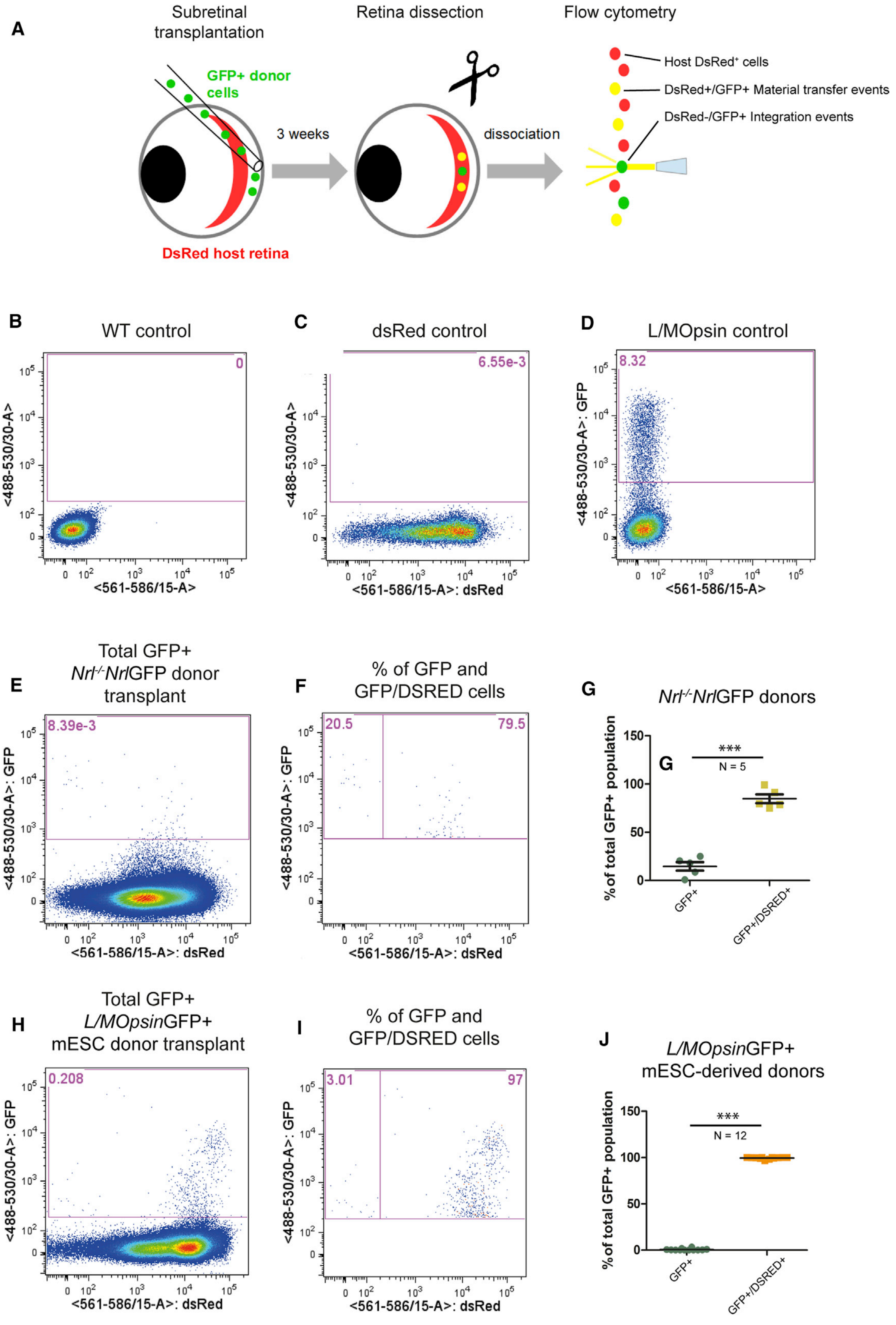

(legend on next page)

presence of small numbers of predominantly rod-like GFP$^+$ cells within the host ONL.

Transplanted Cone and Cone-like Photoreceptors Engage in Material Transfer with Host Photoreceptors in the Intact Wild-Type Retina

We (Pearson et al., 2016), and others (Santos-Ferreira et al., 2016; Singh et al., 2016), recently reported that transplanted donor-derived rod photoreceptor precursors engage in material transfer with photoreceptors in the intact host retina. Given that most of the GFP$^+$ cells within the wild-type recipients bore a striking morphological resemblance to rod photoreceptors, we sought to determine whether these arose from a process of material transfer. Donor-derived P8 *Nrl*$^{-/-}$;*Nrl*-GFP$^+$ cells or mESC-derived d26-29 *L/MOpsin*-GFP$^+$ cells were transplanted into *dsRed*$^{+/-}$ recipients, which have normal retinas but with all the cells ubiquitously expressing the fluorescent reporter, dsRed. At 2–3 weeks post transplantation, host retinas were carefully dissected free from any remaining SRS cell mass, dissociated, and analyzed using flow cytometry (Figure 3). As we reported previously for rods (Pearson et al., 2016), the vast majority of the apparently integrated GFP$^+$ cells co-expressed dsRed (85% ± 10% SD, N = 5 following transplantation of *Nrl*$^{-/-}$;*Nrl*-GFP cells and 99% ± 1%, N = 12 following transplantation of *L/MOpsin*-GFP mESC cones; see note in Supplemental Experimental Procedures). This suggests that transplanted cones and cone-like cells can undergo material transfer with rod photoreceptors in the intact wild-type host retina in a manner resembling that recently described for rod-to-rod transfer (Pearson et al., 2016; Santos-Ferreira et al., 2016; Singh et al., 2016), albeit with apparently poorer efficiency.

Transplantation of Nrl$^{-/-}$ Cone-like Photoreceptor Precursors into Different Retinal Environments

Material transfer appears to account for a significant proportion of the GFP$^+$ cells found within the intact wild-type host ONL after transplantation of rod (Singh et al., 2016; Santos-Ferreira et al., 2016; Pearson et al., 2016) and cone (this paper and Ortin-Martinez et al., 2017; Decembrini et al., 2017) photoreceptors. However, we have previously demonstrated the real-time integration of rod photoreceptors into the disrupted retina of the *Prph2*$^{rd2/rd2}$ (*retinal degeneration slow, rds*) mouse (Pearson et al., 2016) and that some degenerating retinas support better transplantation outcomes (number of GFP$^+$ cells in the host ONL) than others (Barber et al., 2013).

We sought to examine what impact the recipient retinal environment has on transplantation outcome and the relative contributions of material transfer and/or integration by transplanting P8 *Nrl*$^{-/-}$;*Nrl*-GFP$^+$ cone-like cells into models of cone dysfunction and degeneration and into cone-enriched, rod-depleted environments. The first group included *Cnga3*$^{cpfl5/cpfl5}$, which have mislocalized, non-functional cones that degenerate slowly over several weeks; *Pde6c*$^{cpfl1/cpfl1}$, which have non-functional, rapidly degenerating cones; and *Prph2*$^{rd2/rd2}$, in which all photoreceptors fail to produce outer segments and undergo degeneration (rods, then cones) over a period of several months. The second group included the largely non-degenerative but cone-enriched *Nr2e3*$^{rd7/rd7}$ and *Nrl*$^{-/-}$ models.

At 2–3 weeks post transplantation, similar numbers of GFP$^+$ cells were found in the ONL of *Cnga3*$^{cpfl5/cpfl5}$ (354 ± 108, N = 13/17), *Pde6c*$^{cpfl1/cpfl1}$ (149 ± 45, N = 11/13), and *Nr2e3*$^{rd7/rd7}$ (476 ± 147, N = 9/11) hosts as found in wild-type hosts (242 ± 62, N = 10/15) (Figure 4A). They also presented morphological profiles resembling those seen in wild-type retinas; both rod-like (Figure 4B) and cone-like (Figure 4C) morphologies were observed, with most resembling rods in their morphology, location, and IHC profile. Rarely, GFP$^+$/RXRγ$^+$ cells with multichromocenter nuclei were seen correctly located at the apical margin (Figure 4C). No obvious increases in the incidence of cone-like GFP$^+$ cells were seen in the *Pde6c*$^{cpfl1/cpfl1}$ and *Cnga3*$^{cpfl5/cpfl5}$ retinas.

In contrast, markedly higher numbers of GFP$^+$ cells (3,780 ± 1,265, N = 13/15; $p < 0.001$) were seen in the

Figure 3. Transplanted Cone Photoreceptors Undergo Material Transfer with Wild-Type Host Photoreceptors

Nrl$^{-/-}$;*Nrl-GFP* or mESC-derived *L/MOpsin*-GFP post-mitotic photoreceptor precursor donor cells were transplanted into *dsRed* hosts and examined by flow cytometry 2–3 weeks post transplantation.

(A) Schematic of the experimental protocol.

(B–D) Representative flow-cytometry plots for adult (B) wild-type (negative control), (C) *dsRed* (positive control), and (D) *L/MOpsin-GFP* (positive control) retinas. Pink box shows gating for GFP$^+$ cells.

(E and F) Representative plots from an example of a host retina transplanted with *Nrl*$^{-/-}$;*Nrl*-GFP donor cells showing (E) percentage of total retinal cells that were GFP$^+$ (pink box) and (F) the proportion of these that were GFP$^+$ only (left pink box) or GFP$^+$/dsRed$^+$ (right pink box).

(G) Box-and-whiskers plot showing median and range for percentage of GFP$^+$ only and GFP$^+$/dsRed$^+$ photoreceptors within each host retina after transplantation of *Nrl*$^{-/-}$;*Nrl*-GFP donor cells.

(H–J) representative plots (H and I) and box-and-whiskers (SD) plot (J) from retinas transplanted with mESC-derived *L/MOpsin*-GFP donor cells.

***$p < 0.001$, unpaired t test. N, number of eyes.

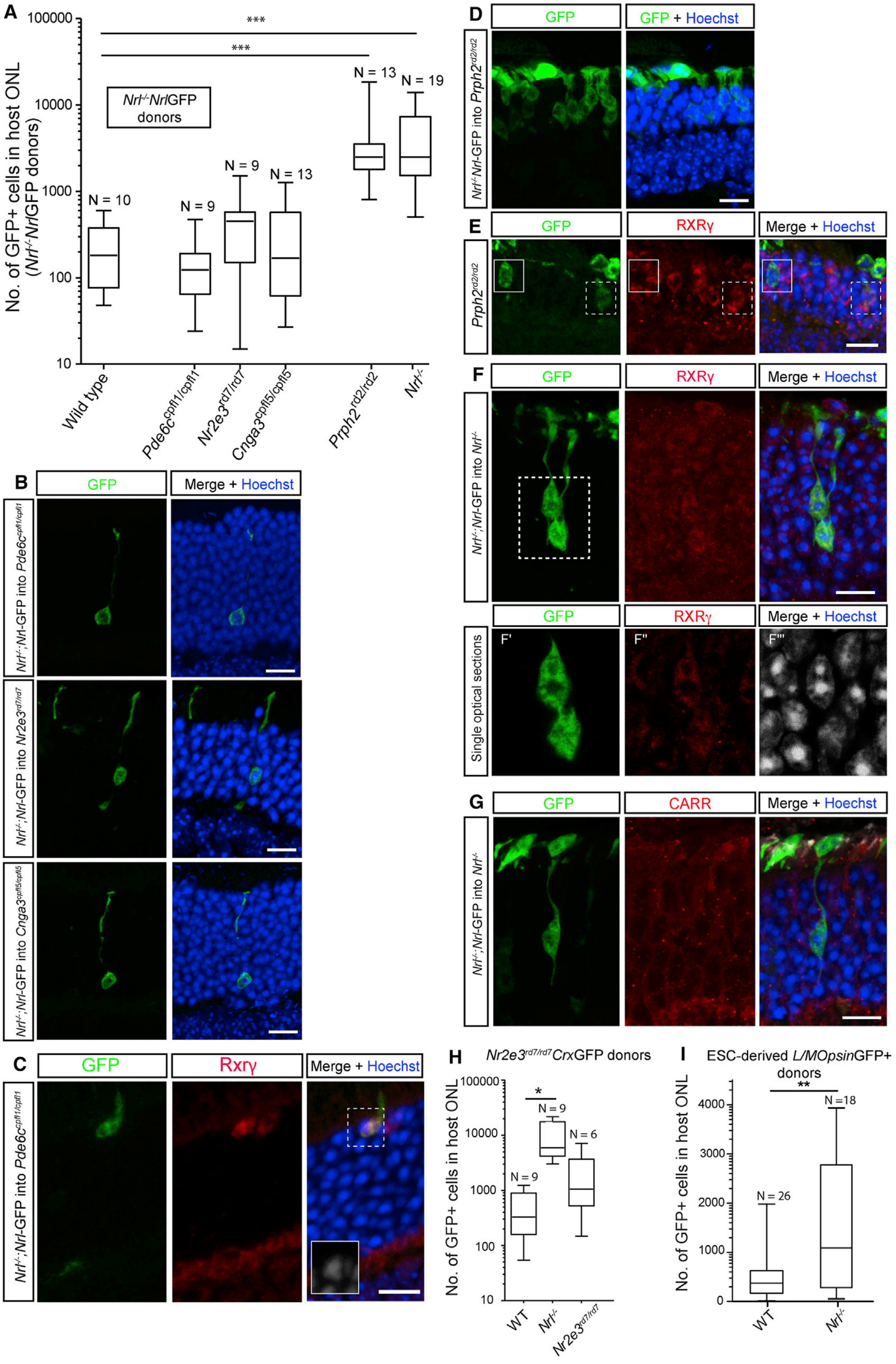

(legend on next page)

Prph2$^{rd2/rd2}$ recipient, compared with wild-type (Figures 4A and 4D). These cells showed a variety of morphologies and IHC profiles, with qualitatively more located at the apical margin and expressing RXRγ (Figure 4E, dotted-line box), although others did not (Figure 4E, solid-line box). Similarly, the number of GFP^{+} cells within the *Nrl*$^{-/-}$ host ONL was significantly higher (4,631 ± 971, N = 19/24; $p < 0.001$) (Figure 4A) than that found in wild-type or any of the other models of cone degeneration, as also recently reported by others (Santos-Ferreira et al., 2015; Smiley et al., 2016). Imaging revealed that GFP^{+} cells within the *Nrl*$^{-/-}$ host presented a cone-like phenotype with enlarged multichromocenter nuclei, typical of both normal cones and the host cone-like cells (Figures 4F and S2G), and expressed RXRγ (Figure 4F), while cone arrestin was more heterogeneous (Figure 4G). Transplantation of P8 *Nr2e3*$^{rd7/rd7}$*Crx*-GFP^{+} cells (Figure 4H) and day 26–29 *L/MOpsin*-GFP^{+} mESC-derived cells (Figure 4I) similarly resulted in significantly higher numbers of GFP^{+} cells within the *Nrl*$^{-/-}$ host ONL (9,690 ± 2,442, N = 9/9, $p < 0.01$ unpaired t test; and 1,415 ± 311, N = 18/18, $p < 0.01$ unpaired t test, respectively) compared with wild-type hosts (503 ± 144, N = 9/18; 466 ± 86, N = 26/26, respectively), indicating that the increase was a consequence of the host environment. Other recipient models were not tested with these donor cells.

Increased Donor Cell Integration Partially Accounts for Increased Numbers of GFP^{+} Cells in Cone-Only Nrl$^{-/-}$ Host Retina

While material transfer likely explains the presence of rod-like cells in the wild-type retina following transplantation of cones, it does not necessarily explain the significantly higher numbers of GFP^{+} cells in disease models, such as the *Nrl*$^{-/-}$ and *Prph*$^{rd2/rd2}$ recipients. We therefore sought to directly investigate the level of donor cell integration into the recipient ONL by performing fluorescence *in situ* hybridization (FISH) for the Y chromosome (Y^{+}) following the transplantation of male donor cells (Figure 5). Male *Nrl*$^{-/-}$;*Nrl*-GFP^{+} donors were transplanted into either female *Nrl*$^{-/-}$ or wild-type hosts. Few, if any, cells within the wild-type ONL bore the Y chromosome (Figure 5A, top). However, we observed many Y^{+} nuclei that were incontrovertibly located within the ONL of host *Nrl*$^{-/-}$ retinas (Figures 5A [middle] and S3A), demonstrating that robust donor cell integration can occur into this model. Technical limitations meant that it was difficult to routinely obtain robust labeling for both GFP and the Y chromosome probe in the same retinal section, but examples are shown in Figure 5B. To quantify the proportion of events ascribable to integration, as opposed to material transfer, in this model we counted the number of GFP^{+} cells within the ONL and performed FISH on different, but consecutive, sections in the same retina. Y^{+} nuclei were found at a ratio of ~1:5, with respect to the number of GFP^{+} cells in consecutive sections in the same eyes, compared with only ~1:100 in wild-type hosts. When expressed as a percentage of the total GFP^{+} cells/eye, Y^{+} nuclei accounted for 23% (±3%; N = 11) of GFP^{+} cells in the same *Nrl*$^{-/-}$ eyes, compared with 1% (±0.8%; N = 5) in the wild-type host (Figure 5B). This indicates that around one-fifth of the GFP^{+} cells seen in the *Nrl*$^{-/-}$ host arise from donor cell integration. Similarly, we observed an increased number of Y^{+} nuclei in the *Prph2*$^{rd2/rd2}$ model (14% ± 3% of total GFP^{+} cells; N = 6). We considered whether there were consistent differences in morphologies potentially arising from integrated cells versus those arising from material transfer, since integrated cells might fail to fully develop. Unfortunately, much of the finer details of the GFP signal, such as the apical and basal processes, are lost when combining with Y-probe staining in the same section (see Figure 5B). This makes it difficult to draw firm conclusions, but at a gross level the Y^{+}, GFP^{+} cell bodies appear quite normal.

In contrast to a very recent report (Ortin-Martinez et al., 2017), these data strongly suggest that integration of cone photoreceptors can occur alongside material transfer, at least in certain host environments. This raises the question of why the *Nrl*$^{-/-}$ retinal environment supports integration while the intact wild-type retina does not. The *Nrl*$^{-/-}$ retina is notably disturbed in its cytoarchitecture: whorls and rosettes are common and the outer limiting membrane (OLM) is also disturbed in this model (Stuck et al., 2012). Since we have previously demonstrated that OLM integrity influences transplantation outcome (Pearson et al., 2010; West et al., 2008; Barber et al.,

Figure 4. Transplantation Outcome Is Dependent on the Host Environment

(A) Box-and-whiskers (SD) plot showing the influence of host environment on the number of GFP^{+} cells in the host ONL after transplantation of genetically engineered cone-like *Nrl*$^{-/-}$;*Nrl*-GFP cells. Many more cells were found in the *Nrl*$^{-/-}$ and *Prph2*$^{rd2/rd2}$ hosts than any other model.

(B–G) Confocal images showing representative examples of GFP^{+} cells in the different host retinas. In (E) and (F) solid-line box depicts GFP^{+}/RXRγ^{-} cells and dashed-line box depicts GFP^{+}/RXRγ^{+} cells.

(H and I) Box-and-whiskers plots showing that the *Nrl*$^{-/-}$ host environment supports similarly increased numbers of GFP^{+} cells after transplantation of (H) *Nr2e3*$^{rd7/rd7}$;*Crx*-GFP photoreceptors and (I) mESC-derived *L/MOpsin*-GFP^{+} cones.

ANOVA with correction for multiple comparisons: ***$p < 0.001$, **$p < 0.01$, *$p < 0.05$. N, number of eyes. Scale bars, 10 μm.

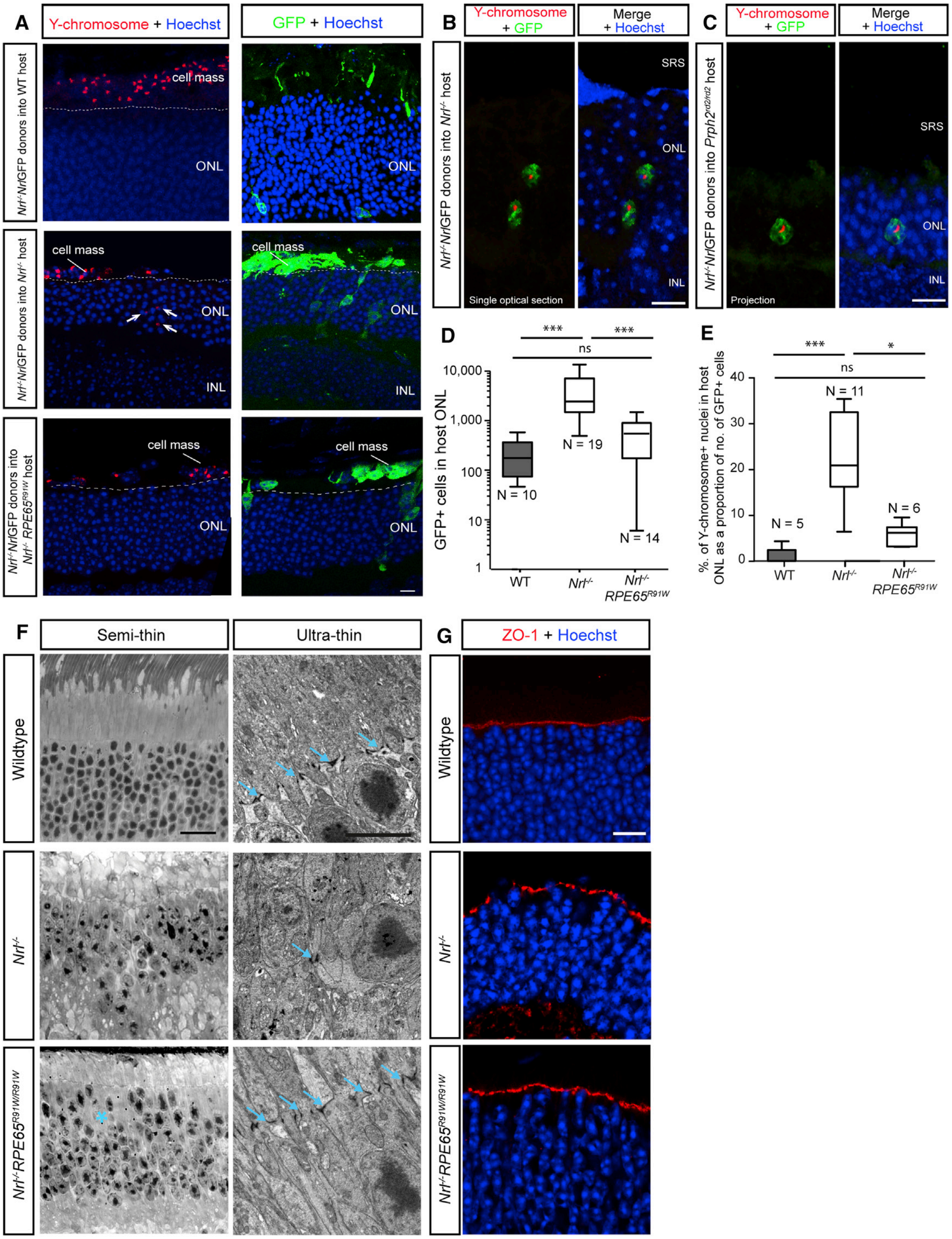

Figure 5. Transplanted Cone Photoreceptors Integrate in Some Models of Retinal Degeneration

(A) Confocal images of FISH and GFP labeling in consecutive serial sections (exception being wild-type where GFP is from a different region), showing significant numbers of Y chromosome^{+} (red) nuclei in *Nrl*$^{-/-}$ host ONL (arrows) but few, if any, in wild-type or *Nrl*$^{-/-}$;*RPE65*$^{R91W/R91W}$ host ONL, after transplantation of P7/8 *Nrl*$^{-/-}$;*Nrl*-GFP donors. Cell masses located in SRS also demonstrate widespread labeling.

(B and C) examples of retinal sections co-stained for both GFP and Y chromosome following transplantation of *Nrl*$^{-/-}$;*Nrl*-GFP into (B) *Nrl*$^{-/-}$ and (C) *Prph2*$^{rd2/rd2}$ hosts.

(legend continued on next page)

2013), we considered whether the increases in cone precursor integration observed in the *Nrl*$^{-/-}$ might be explained by its disturbed cytoarchitecture. A variant of the *Nrl*$^{-/-}$ model, the *Nrl*$^{-/-}$;*RPE65*$^{R91W/R91W}$ mouse, carries an additional point mutation on the *RPE65* gene, which is reported to prevent the appearance of whorls and rosettes and preserve the OLM (Samardzija et al., 2014). We performed ultrastructural analysis (Figure 5F) and IHC (Figure 5G) analysis of both the *Nrl*$^{-/-}$ and *Nrl*$^{-/-}$;*RPE65*$^{R91W/R91W}$ retina. In the *Nrl*$^{-/-}$, the adherens junctions are much sparser compared with wild-type, with the majority forming between Müller glial cells (Figure 5F, blue arrows; and A.B.G. and R.A.P., unpublished data). These alterations were particularly evident around regions of rosette formation. Conversely, rosettes were absent in the *Nrl*$^{-/-}$;*RPE65*$^{R91W/R91W}$ retina, as previously reported, although there were some subtle disturbances in ONL lamination (Figure 5F, blue asterisk). Despite this, ultrastructural analysis demonstrated the presence of typical photoreceptor-Müller glial cell adherens junctions, indicating that the OLM is largely intact in this model (Figure 5F, blue arrows). A significantly lower number of GFP^{+} cells was seen in the host ONL following transplantation of P8 *Nrl*$^{-/-}$;*Nrl*-GFP donor cells into the *Nrl*$^{-/-}$;*RPE65*$^{R91W/R91W}$ model, compared with *Nrl*$^{-/-}$ hosts (584 ± 116; N = 10/14; compared with 4,631 ± 971, N = 19/23; Figures 5A and 5D). Moreover, the proportion of these cells that were Y^{+} was significantly lower than in the *Nrl*$^{-/-}$ host (6% ± 1%; N = 6) (Figures 5A and 5E) and more similar to that seen in wild-type hosts.

Together, these data indicate that the retinal environment of *Nrl*$^{-/-}$ and *Prph2*$^{rd2/rd2}$ hosts is able to support donor cone photoreceptor integration alongside material transfer, while the wild-type retina supports only very limited cone integration. The cytoarchitecture of the host retina is likely to play a major role in determining the relative contributions of these two mechanisms to transplantation outcome.

DISCUSSION

The transplantation of healthy photoreceptor precursors, derived from both stem cells and donor retinas, has been shown to restore aspects of visual function in animal models of retinal degeneration (Santos-Ferreira et al., 2015; Pearson et al., 2012; Singh et al., 2013; Barnea-Cramer et al., 2016). Previously, this was understood to occur by a process of donor cell migration and integration within the partially intact recipient retina. However, we (Pearson et al., 2016) and others (Santos-Ferreira et al., 2016; Singh et al., 2016) have recently shown that instead, when transplanted into the non-degenerative retina, where host photoreceptors remain, donor rod photoreceptors engage in a process of material transfer with host photoreceptors, which appears to lead to the exchange of RNA and/or protein in a robust, transient, and repeatable manner (Pearson et al., 2016).

Much less is known about the transplantation of cone photoreceptors and whether they could engage in a similar process. Early studies on cone transplantation reported a preponderance of GFP-labeled cells that morphologically resembled rods (Lakowski et al., 2010) but, since a mixed population of rod and cone donors was used, it was hypothesized that this may represent a change in cell fate or a preference of rods to integrate over cones. Recently, others reported similar findings using a variety of cone and cone-like donor cells (Santos-Ferreira et al., 2015; Smiley et al., 2016). In light of our recent findings regarding material transfer between rods, we sought to determine whether the rod-like cells seen after transplantation of cones also arise from material transfer. Here, we demonstrate that, irrespective of their origin, cone photoreceptors can engage in material transfer with host rod and cone photoreceptors. Given that this occurred both for stem cell-derived cones and genetically engineered cone-like cells, material transfer is likely to account for all previous reports of rod-like phenotypes following cone transplantation into the intact, non-degenerative murine retina (Smiley et al., 2016; Santos-Ferreira et al., 2015; Lakowski et al., 2010). Importantly, however, we also find that cone donors are capable of reasonably efficient integration when the host retinal structure is disrupted. This is important in the light of the recent publications on material transfer. It suggests that it may be possible to improve the levels of integration with appropriate manipulations of the host environment, as previously envisaged (see Pearson, 2014). It also highlights the need for careful

(D) Box-and-whiskers (SD) plot of total number of GFP^{+} cells in the Nrl$^{-/-}$ host ONL, compared with *Nrl*$^{-/-}$;*RPE65*$^{R91W/R91W}$ and wild-type. ANOVA with correction for multiple comparisons: ***p < 0.001; ns, not significant.
(E–G) Box-and-whiskers (SD) plot (E) of total number of Y chromosome^{+} nuclei, as a proportion of total number of GFP^{+} cells, located within the host ONL in wild-type, *Nrl*$^{-/-}$, and *Nrl*$^{-/-}$;*RPE65*$^{R91W/R91W}$ recipients. Kruskal-Wallis test: ***p < 0.001; *p < 0.05; ns, not significant. N, number of eyes. Ultrastructural analysis (F) and IHC (G) for the OLM marker, ZO-1 (red) and the gliosis marker, Gfap (green) show disruption in OLM integrity in the *Nrl*$^{-/-}$, but not the *Nrl*$^{-/-}$;*RPE65*$^{R91W/R91W}$ or wild-type, retina. Blue arrows denote adherens junctions; blue asterisk denotes region of ONL disruption.
Scale bars, 10 μm (A–C, G), 25 μm (F, semi-thin), and 5 μm (F, ultrathin).

characterization of the relative contributions each made by material transfer and donor integration when assessing transplantation outcome in future studies.

The recent reports of material transfer between donor and host photoreceptors (Santos-Ferreira et al., 2016; Pearson et al., 2016; Decembrini et al., 2017; Singh et al., 2016; Ortin-Martinez et al., 2017) requires a significant re-evaluation of the cellular mechanisms underlying rescue by photoreceptor transplantation, at least in those recipients where some host photoreceptors remain. While the mechanisms are at present unknown, this current study and other recently published papers begin to provide some direction. Transplantation outcome, defined as the number of GFP$^+$ cells within the host ONL, is dependent upon the developmental stage of the donor cell and that post-mitotic cells yielded better outcomes than progenitors (Pearson et al., 2012; Lakowski et al., 2010; MacLaren et al., 2006; Gonzalez-Cordero et al., 2013). Although we now understand the predominant mechanism to be material transfer, the developmental correlation with this process remains true: post-mitotic rods (Pearson et al., 2016; Santos-Ferreira et al., 2016) and cones derived from post-natal stages engage in material transfer more effectively than immature retinal cells. In a very recent report, Arsenijevic and colleagues (Decembrini et al., 2017) transplanted *Chrnb4*-EGFP donor-derived cones, as we have done here, and similarly concluded that material transfer is a developmentally regulated phenomenon.

A comparison of the findings reported here and by others (Smiley et al., 2016; Ortin-Martinez et al., 2017; Decembrini et al., 2017) following transplantation of various cone and cone-like populations into the intact wild-type retina, with those following transplantation of rod photoreceptors (Santos-Ferreira et al., 2016; Pearson et al., 2016) shows that the numbers of GFP$^+$ cells within the host ONL are much lower using cone, compared with rod, donors (hundreds versus many thousands). This might indicate that material transfer is less efficient from donor cones to host rods (and cones). Alternatively, material transfer may require the continued presence of donor cells in the SRS and cones may survive less well than rods, or a combination of these. Supporting the need for donor survival, we recorded a surprisingly high number of host eyes with very few donor cones within the SRS, compared with equivalent transplants made with rod or mixed populations of donors (Pearson et al., 2012, 2016; Lakowski et al., 2010) or indeed the genetically engineered cone-like donor populations. Previously, we reported that manipulation of the immune system prolonged the presence of GFP$^+$ cells in the wild-type host ONL (West et al., 2010), a finding that can be, perhaps, alternatively explained by prolonging the survival of donor cells available to provide material for transfer. Similarly, two other recent reports reported that modulation of the host immune environment permitted sustained survival of transplanted cells in the SRS (Zhu et al., 2017; Neves et al., 2016), and, from our interpretation of the published images, what appears to include robust levels of material transfer (see also MacLaren, 2017). The dependency on donor developmental stage and the apparent need for the sustained presence of donor cells may provide clues as to the cellular mechanisms underlying material transfer.

Transplants into wild-type hosts showed surprising heterogeneity with respect to cone marker expression by the GFP$^+$ cells, with variable expression of CARR and very rare expression of RXRγ. Wallace and colleagues reported minimal cone marker expression (Smiley et al., 2016), while Ader and colleagues reported expression of a number of cone markers, although they did not indicate how common such expression was (Santos-Ferreira et al., 2015). Future investigations will require careful quantitative analysis of the marker expression profile of both donor cells and GFP$^+$ within the host; these will be important to our understanding of what can, and cannot, be exchanged by material transfer and what the broader implications of material transfer and its potential utility might be.

As previously observed for rod transplantation (Barber et al., 2013), we report here that the efficacy of cone transplantation, as determined by the number of GFP$^+$ cells in the host ONL, is critically dependent on the host environment. Non-degenerative models behave like wild-type hosts, resulting in the presence of small numbers of GFP$^+$ cells within the ONL, the majority arising through material transfer. In another recent study, Arsenijevic and colleagues (Decembrini et al., 2017) transplanted *Chrnb4*-EGFP$^+$ cones in the *Cnga3*$^{-/-}$ and the *Nrl*$^{-/-}$*RPE65*$^{R91W/R91W}$ mouse lines. They reported low numbers of reporter-labeled cells, similar to what we report here following transplantation of genetically engineered cone-like cells into the same models. They note that GFP$^+$ cell numbers could be higher in localized areas and propose that these may reflect areas of OLM disruption, possibly through injection trauma. This fits with our own observations that significantly higher numbers of GFP$^+$ cells were found in the *Nrl*$^{-/-}$ and *Prph2*$^{rd2/rd2}$ host retinas, both models that display highly disrupted OLM (this study; also Samardzija et al., 2014; Hippert et al., 2015; Barber et al., 2013). These models are also unusual in that they are composed largely of cone, rather than rod, cells. This might suggest that a cone-enriched environment better supports cone integration. Arguing against this, however, are the low levels of integration seen in the *Nrl*$^{-/-}$*RPE65*$^{R91W/R91W}$ mouse retina, which is also cone enriched but whose cytoarchitecture is largely intact.

Interestingly, Wallace and colleagues (Ortin-Martinez et al., 2017) transplanted *Ccdc136*-GFP$^+$ (cones) and

$Nrl^{-/-}$*Ccdc136*-GFP^+ (cone-like) cells into the $Nrl^{-/-}$ host retina and, like we report here, noted a significant increase in the number of GFP cells in the $Nrl^{-/-}$ hosts compared with wild-type. However, they concluded that this increase was due entirely to an increase in material transfer and that this preferentially occurred in regions of OLM disruption. Their conclusions were based on the use of 5-ethynyl-2′-deoxyuridine (EdU) to pre-label a proportion of the donor cone nuclei and the failure to see any GFP^+ cells within the host ONL that also bore EdU. In contrast, by using FISH to label the Y chromosome of male donors, we could conclude that at least a proportion of the increase in GFP^+ cells in the host $Nrl^{-/-}$ ONL is the result of actual donor cell integration within the host retina. The reasons for the discrepancy are not clear. Some reports have described potentially toxic effects of EdU, and we (Warre-Cornish et al., 2014) and others (Andersen et al., 2013; Ligasova et al., 2015; Neef and Luedtke, 2011) have reported detrimental effects on cell migration and survival. It is possible that such effects may account for the absence of integrated cells in the study by Wallace and colleagues (Ortin-Martinez et al., 2017). Conversely, our experimental design makes use of an endogenous marker revealed after transplantation, which is less likely to have any unintended impact upon the normal behavior of transplanted photoreceptors. Alternatively, the age of the host animal and concomitant stage of degeneration at the time of transplantation may affect the degree of donor cell integration, but may not be material transfer, as other potential barriers, such as glial hypertrophy, may start to impede donor cell integration (Hippert et al., 2015, 2016; Barber et al., 2013). Regardless, it is important to emphasize that the increase seen in this model is not due *only* to increased integration. A significant proportion of the increase in GFP^+ cells in $Nrl^{-/-}$, compared with wild-type, must be due also to increased material transfer, alongside increased integration. Poor transplantation outcomes, which we currently assume encompasses a varying proportion of material transfer and donor cell integration, are often correlated with models that have high levels of CSPG deposition (Hippert et al., 2015; Barber et al., 2013) and/or maintain OLM integrity. Conversely, disruption of the OLM (Pearson et al., 2010; West et al., 2008) and breakdown of CSPGs (Singhal et al., 2008; Barber et al., 2013; Suzuki et al., 2007) each facilitate better transplantation outcomes. Future work needs to determine to what extent the parallel processes of integration and material transfer contribute to transplantation outcome. Moreover, elucidation of the cellular mechanisms behind material transfer will help establish how manipulations of the degenerative environment act to improve the efficiency of this unusual and surprising process.

Recent reports have described the transplantation of genetically engineered cone-like cells (this study and Smiley et al., 2016; Santos-Ferreira et al., 2015; Ortin-Martinez et al., 2017; Decembrini et al., 2017) and their ability to rescue cone-mediated function (Santos-Ferreira et al., 2015). Similarly, we reported restoration of rod-mediated function following the transplantation of rod donor cells into the intact, but non-functional, *Gnat1*$^{-/-}$ recipient (Pearson et al., 2012). In view of the recent findings, it is likely that these rescues were achieved by material transfer mediating the restoration of functional levels of the proteins missing in the host photoreceptors. This opens new interesting avenues of investigation, as it may be possible to harness the mechanisms mediating material transfer for the restoration of visual function in progressive retinal degeneration. Regarding the current study, the numbers of GFP^+ cells found following cone transplantation is markedly lower than that seen for rod transplantation, even in the $Nrl^{-/-}$ host. For this reason, we did not explore whether these cells were functional, although others have reported some restoration of function following transplantation of the same donor population ($Nrl^{-/-}$ [Santos-Ferreira et al., 2015]). In the current study we find a significant increase in the number of true integration events, compared with material transfer; it will be of significant future interest to determine to what degree these cells are capable of contributing to vision, compared with those resulting from material transfer.

Together, our data demonstrate that the transplantation of cone photoreceptors results in both material transfer and donor cell integration, and the relative contribution of these two processes is likely to depend on the etiology of disease and the host retinal environment.

EXPERIMENTAL PROCEDURES

All animal studies were carried out under the Animals (Scientific Procedures) Act 1986 under a project license PPL 70/8120 issued by the UK Government Home Office and in accordance with protocols approved by the Animal Welfare and Ethics Committee of the UCL Institute of Ophthalmology.

All means are stated ±SD except for cell counts, which are stated as ±SEM. N denotes number of eyes examined and n the number of cells, where appropriate. Full details of experimental methods are provided in Supplemental Experimental Procedures.

Mouse ESC Culture and Retinal Differentiation

Retinal differentiation was achieved using a mouse EK.CCE ESC line (Evans and Kaufman, 1981) and an adapted 2D/3D culture system, as described in Kruczek et al. (2017), which includes the addition of 1 mM taurine and 500 nM retinoic acid from day 14 of culture onward. Embryoid bodies were labeled with ShH10.L/MOpsin.GFP on day 20 of culture and dissociated for transplantation on days 26–30.

Dissociation and Fluorescence-Activated Cell Sorting

Embryonic or postnatal neural retinas or mESC-derived oganoids were dissociated using a papain-based Neural Tissue Dissociation Kit (Miltenyl Biotec) prior to sorting on a BD Influx Cell Sorter (Becton Dickinson). Flow-sorted GFP^+ cells were on average >95% pure and >80% viable. Cells were resuspended at a final concentration of 200,000 live cells/μL in sterile EBSS and DNase I (50 U/mL) before injection, unless otherwise stated.

Transplantation of Donor Photoreceptors

One microliter of cell suspension was injected subretinally into the superior retina. Adult mice from different strains were used at ages as denoted in Table S1. All animals were housed under a normal 12/12-hr light/dark cycle. Eyes were harvested 2 weeks after transplantation.

Immunohistochemistry and Cell Counts

Eyes were fixed for 30 min in 4% formaldehyde, washed with PBS, and incubated overnight in 20% (w/v) sucrose, prior to embedding in OCT matrix. Tissue was cut into 18-μm cryosections mounted on glass slides, air-dried for 20 min, and kept frozen at −20°C for use in immunostaining. Staining protocol and the antibodies used are listed in Supplemental Experimental Procedures.

Cell counts were performed in a blinded manner after immunostaining with fluorescein isothiocyanate-conjugated anti-GFP antibody. In every third cryosection, all GFP^+ cells with cell bodies located in the ONL were counted and assessed, when possible, for co-staining and morphology. The total number of cells for each injected eye was calculated as three times this count.

SUPPLEMENTAL INFORMATION

Supplemental Information includes Supplemental Experimental Procedures, three figures, and seven tables and can be found with this article online at https://doi.org/10.1016/j.stemcr.2017.12.008.

AUTHOR CONTRIBUTIONS

P.V.W. contributed to the conception, design, execution, and analysis of most experiments; F.d.M. contributed to the execution and analysis of several of the donor-derived transplantation experiments; K.K. contributed to the design, execution, and analysis of all mESC-derived transplantation experiments; J.R. designed and executed experiments regarding FISH and contributed to histological processing; A.B.G. designed and executed OLM experiments; C.H. generated mouse lines; N.D.A., G.G., A.A.K., and D.G. contributed to transplantation experiments; A.C.B. performed the *OPN1LW*-EGFP experiments; Y.D. contributed to histological processing and surgery; S.J.L.B. and M.K. contributed to ESC maintenance and histological processing; E.Z.A. contributed to cell preparations and animal maintenance; R.D.S. performed FACS and flow analysis; J.W.B.B. contributed to surgery; A.J.S. and A.G.-C. contributed to experimental design and interpretation of experiments; R.R.A. and J.C.S. contributed to the conception and design of experiments, manuscript writing, and funding; R.A.P. contributed to the conception, design, execution, analysis, and interpretation of experiments, subretinal surgery, and funding and wrote the manuscript.

ACKNOWLEDGMENTS

We thank R. Maswood, O. Semenyuk, S. Azam, and A. Michacz for technical support in IoO vector production facility, A. Naeem and M. Fernando for technical support in mESC maintenance, L. Abelleira-Hervas for animal husbandry, and Dr. P. Munro and Dr. M. Hayes for electron microscopy support. We thank the UCL Institute of Child Health (A. Eddaoudi, T. Adejumo) flow cytometry facilities for FACS support. This work was supported by the Medical Research Council UK (mr/j004553/1); Fight for Sight (1448/1449 and 1351/2); the European Research Council (ERC-2012-ADG_20120314); RP Fighting Blindness (GR576); Moorfields Eye Charity; the National Institute for Health Research (NIHR) Biomedical Research Centre for Ophthalmology at Moorfields Eye Hospital and UCL Institute of Ophthalmology (BRC2-007); the Macular Vision Research Foundation; the Child Health Research Appeal Trust; and the NIHR Biomedical Research Centre for Pediatric Research at Great Ormond Street Hospital for Children and UCL Institute of Child Health. P.W. is a Wellcome Trust/MRC 4th-year Neuroscience PhD student. K.K. is a Wellcome Trust 4th-year Stem Cells and Development PhD student. A.G. is an MRC DTP Clinical Neuroscience PhD student. N.A. is a UCL Grand Challenge PhD student. A.G.C. is UCL Sensory Systems and Therapies Fellow. J.W.B.B. is an NIHR Research Professor. J.C.S. is supported by the NIHR Great Ormond Street Hospital Biomedical Research Centre (GOSH BRC) and GOSHCC. R.A.P. is a Royal Society University Research Fellow (UF120046) and supported by an Alcon Research Institute Young Investigator Award.

Received: June 14, 2017
Revised: December 8, 2017
Accepted: December 8, 2017
Published: January 4, 2018

REFERENCES

Andersen, D.C., Skovrind, I., Christensen, M.L., Jensen, C.H., and Sheikh, S.P. (2013). Stem cell survival is severely compromised by the thymidine analog EdU (5-ethynyl-2′-deoxyuridine), an alternative to BrdU for proliferation assays and stem cell tracing. Anal. Bioanal. Chem. *405*, 9585–9591.

Barber, A.C., Hippert, C., Duran, Y., West, E.L., Bainbridge, J.W., Warre-Cornish, K., Luhmann, U.F., Lakowski, J., Sowden, J.C., Ali, R.R., and Pearson, R.A. (2013). Repair of the degenerate retina by photoreceptor transplantation. Proc. Natl. Acad. Sci. USA *110*, 354–359.

Barnea-Cramer, A.O., Wang, W., Lu, S.J., Singh, M.S., Luo, C., Huo, H., Mcclements, M.E., Barnard, A.R., Maclaren, R.E., and Lanza, R. (2016). Function of human pluripotent stem cell-derived photoreceptor progenitors in blind mice. Sci. Rep. *6*, 29784.

Bartsch, U., Oriyakhel, W., Kenna, P.F., Linke, S., Richard, G., Petrowitz, B., Humphries, P., Farrar, G.J., and Ader, M. (2008). Retinal cells integrate into the outer nuclear layer and differentiate into mature photoreceptors after subretinal transplantation into adult mice. Exp. Eye Res. *86*, 691–700.

Decembrini, S., Martin, C., Sennlaub, F., Chemtob, S., Biel, M., Samardzija, M., Moulin, A., Behar-Cohen, F., and Arsenijevic, Y.

(2017). Cone genesis tracing by the Chrnb4-EGFP mouse line: evidences of cellular material fusion after cone precursor transplantation. Mol. Ther. *25*, 634–653.

Evans, M.J., and Kaufman, M.H. (1981). Establishment in culture of pluripotential cells from mouse embryos. Nature *292*, 154–156.

Gonzalez-Cordero, A., West, E.L., Pearson, R.A., Duran, Y., Carvalho, L.S., Chu, C.J., Naeem, A., Blackford, S.J., Georgiadis, A., Lakowski, J., et al. (2013). Photoreceptor precursors derived from three-dimensional embryonic stem cell cultures integrate and mature within adult degenerate retina. Nat. Biotechnol. *31*, 741–747.

Hippert, C., Graca, A.B., Barber, A.C., West, E.L., Smith, A.J., Ali, R.R., and Pearson, R.A. (2015). Muller glia activation in response to inherited retinal degeneration is highly varied and disease-specific. PLoS One *10*, e0120415.

Hippert, C., Graca, A.B., and Pearson, R.A. (2016). Gliosis can impede integration following photoreceptor transplantation into the diseased retina. Adv. Exp. Med. Biol. *854*, 579–585.

Kruczek, K., Gonzalez-Cordero, A., Goh, D., Naeem, A., Jonikas, M., Blackford, S.J.I., Kloc, M., Duran, Y., Georgiadis, A., Sampson, R.D., et al. (2017). Differentiation and transplantation of embryonic stem cell-derived cone photoreceptors into a mouse model of end-stage retinal degeneration. Stem Cell Reports *8*, 1659–1674.

Lakowski, J., Baron, M., Bainbridge, J., Barber, A.C., Pearson, R.A., Ali, R.R., and Sowden, J.C. (2010). Cone and rod photoreceptor transplantation in models of the childhood retinopathy Leber congenital amaurosis using flow-sorted Crx-positive donor cells. Hum. Mol. Genet. *19*, 4545–4559.

Ligasova, A., Strunin, D., Friedecky, D., Adam, T., and Koberna, K. (2015). A fatal combination: a thymidylate synthase inhibitor with DNA damaging activity. PLoS One *10*, e0117459.

MacLaren, R.E. (2017). Cone fusion confusion in photoreceptor transplantation. Stem Cell Investig. *4*, 71.

MacLaren, R.E., Pearson, R.A., Macneil, A., Douglas, R.H., Salt, T.E., Akimoto, M., Swaroop, A., Sowden, J.C., and Ali, R.R. (2006). Retinal repair by transplantation of photoreceptor precursors. Nature *444*, 203–207.

Neef, A.B., and Luedtke, N.W. (2011). Dynamic metabolic labeling of DNA in vivo with arabinosyl nucleosides. Proc. Natl. Acad. Sci. USA *108*, 20404–20409.

Neves, J., Zhu, J., Sousa-Victor, P., Konjikusic, M., Riley, R., Chew, S., Qi, Y., Jasper, H., and Lamba, D.A. (2016). Immune modulation by MANF promotes tissue repair and regenerative success in the retina. Science *353*, aaf3646.

Ortin-Martinez, A., Tsai, E.L., Nickerson, P.E., Bergeret, M., Lu, Y., Smiley, S., Comanita, L., and Wallace, V.A. (2017). A reinterpretation of cell transplantation: GFP transfer from donor to host photoreceptors. Stem Cells *35*, 932–939.

Pearson, R.A. (2014). Advances in repairing the degenerate retina by rod photoreceptor transplantation. Biotechnol. Adv. *32*, 485–491.

Pearson, R.A., Barber, A.C., Rizzi, M., Hippert, C., Xue, T., West, E.L., Duran, Y., Smith, A.J., Chuang, J.Z., Azam, S.A., et al. (2012). Restoration of vision after transplantation of photoreceptors. Nature *485*, 99–103.

Pearson, R.A., Barber, A.C., West, E.L., Maclaren, R.E., Duran, Y., Bainbridge, J.W., Sowden, J.C., and Ali, R.R. (2010). Targeted disruption of outer limiting membrane junctional proteins (Crb1 and ZO-1) increases integration of transplanted photoreceptor precursors into the adult wild-type and degenerating retina. Cell Transplant. *19*, 487–503.

Pearson, R.A., Gonzalez-Cordero, A., West, E.L., Ribeiro, J.R., Aghaizu, N., Goh, D., Sampson, R.D., Georgiadis, A., Waldron, P.V., Duran, Y., et al. (2016). Donor and host photoreceptors engage in material transfer following transplantation of post-mitotic photoreceptor precursors. Nat. Commun. *7*, 13029.

Samardzija, M., Caprara, C., Heynen, S.R., Willcox Deparis, S., Meneau, I., Traber, G., Agca, C., von Lintig, J., and Grimm, C. (2014). A mouse model for studying cone photoreceptor pathologies. Invest. Ophthalmol. Vis. Sci. *55*, 5304–5313.

Santos-Ferreira, T., Llonch, S., Borsch, O., Postel, K., Haas, J., and Ader, M. (2016). Retinal transplantation of photoreceptors results in donor-host cytoplasmic exchange. Nat. Commun. *7*, 13028.

Santos-Ferreira, T., Postel, K., Stutzki, H., Kurth, T., Zeck, G., and Ader, M. (2015). Daylight vision repair by cell transplantation. Stem Cells *33*, 79–90.

Siegert, S., Cabuy, E., Scherf, B.G., Kohler, H., Panda, S., Le, Y.Z., Fehling, H.J., Gaidatzis, D., Stadler, M.B., and Roska, B. (2012). Transcriptional code and disease map for adult retinal cell types. Nat. Neurosci. *15*, 487–495, S1-2.

Singh, M.S., Balmer, J., Barnard, A.R., Aslam, S.A., Moralli, D., Green, C.M., Barnea-Cramer, A., Duncan, I., and Maclaren, R.E. (2016). Transplanted photoreceptor precursors transfer proteins to host photoreceptors by a mechanism of cytoplasmic fusion. Nat. Commun. *7*, 13537.

Singh, M.S., Charbel Issa, P., Butler, R., Martin, C., Lipinski, D.M., Sekaran, S., Barnard, A.R., and Maclaren, R.E. (2013). Reversal of end-stage retinal degeneration and restoration of visual function by photoreceptor transplantation. Proc. Natl. Acad. Sci. USA *110*, 1101–1106.

Singhal, S., Lawrence, J.M., Bhatia, B., Ellis, J.S., Kwan, A.S., Macneil, A., Luthert, P.J., Fawcett, J.W., Perez, M.T., Khaw, P.T., and Limb, G.A. (2008). Chondroitin sulfate proteoglycans and microglia prevent migration and integration of grafted Muller stem cells into degenerating retina. Stem Cells *26*, 1074–1082.

Smiley, S., Nickerson, P.E., Comanita, L., Daftarian, N., El-Sehemy, A., Tsai, E.L., Matan-Lithwick, S., Yan, K., Thurig, S., Touahri, Y., et al. (2016). Establishment of a cone photoreceptor transplantation platform based on a novel cone-GFP reporter mouse line. Sci. Rep. *6*, 22867.

Stuck, M.W., Conley, S.M., and Naash, M.I. (2012). Defects in the outer limiting membrane are associated with rosette development in the Nrl$^{-/-}$ retina. PLoS One *7*, e32484.

Suzuki, T., Akimoto, M., Imai, H., Ueda, Y., Mandai, M., Yoshimura, N., Swaroop, A., and Takahashi, M. (2007). Chondroitinase ABC treatment enhances synaptogenesis between transplant and host neurons in model of retinal degeneration. Cell Transplant. *16*, 493–503.

Warre-Cornish, K., Barber, A.C., Sowden, J.C., Ali, R.R., and Pearson, R.A. (2014). Migration, integration and maturation of

photoreceptor precursors following transplantation in the mouse retina. Stem Cells Dev. *23*, 941–954.

West, E.L., Pearson, R.A., Barker, S.E., Luhmann, U.F., Maclaren, R.E., Barber, A.C., Duran, Y., Smith, A.J., Sowden, J.C., and Ali, R.R. (2010). Long-term survival of photoreceptors transplanted into the adult murine neural retina requires immune modulation. Stem Cells *28*, 1997–2007.

West, E.L., Pearson, R.A., Tschernutter, M., Sowden, J.C., Maclaren, R.E., and Ali, R.R. (2008). Pharmacological disruption of the outer limiting membrane leads to increased retinal integration of transplanted photoreceptor precursors. Exp. Eye Res. *86*, 601–611.

Young, R.W. (1985). Cell differentiation in the retina of the mouse. Anat. Rec. *212*, 199–205.

Zhu, J., Cifuentes, H., Reynolds, J., and Lamba, D.A. (2017). Immunosuppression via loss of IL2rgamma enhances long-term functional integration of hESC-derived photoreceptors in the mouse retina. Cell Stem Cell *20*, 374–384.e5.

Stem Cell Reports, Volume *10*

Supplemental Information

Transplanted Donor- or Stem Cell-Derived Cone Photoreceptors Can Both Integrate and Undergo Material Transfer in an Environment-Dependent Manner

Paul V. Waldron, Fabiana Di Marco, Kamil Kruczek, Joana Ribeiro, Anna B. Graca, Claire Hippert, Nozie D. Aghaizu, Aikaterini A. Kalargyrou, Amanda C. Barber, Giulia Grimaldi, Yanai Duran, Samuel J.I. Blackford, Magdalena Kloc, Debbie Goh, Eduardo Zabala Aldunate, Robert D. Sampson, James W.B. Bainbridge, Alexander J. Smith, Anai Gonzalez-Cordero, Jane C. Sowden, Robin R. Ali, and Rachael A. Pearson

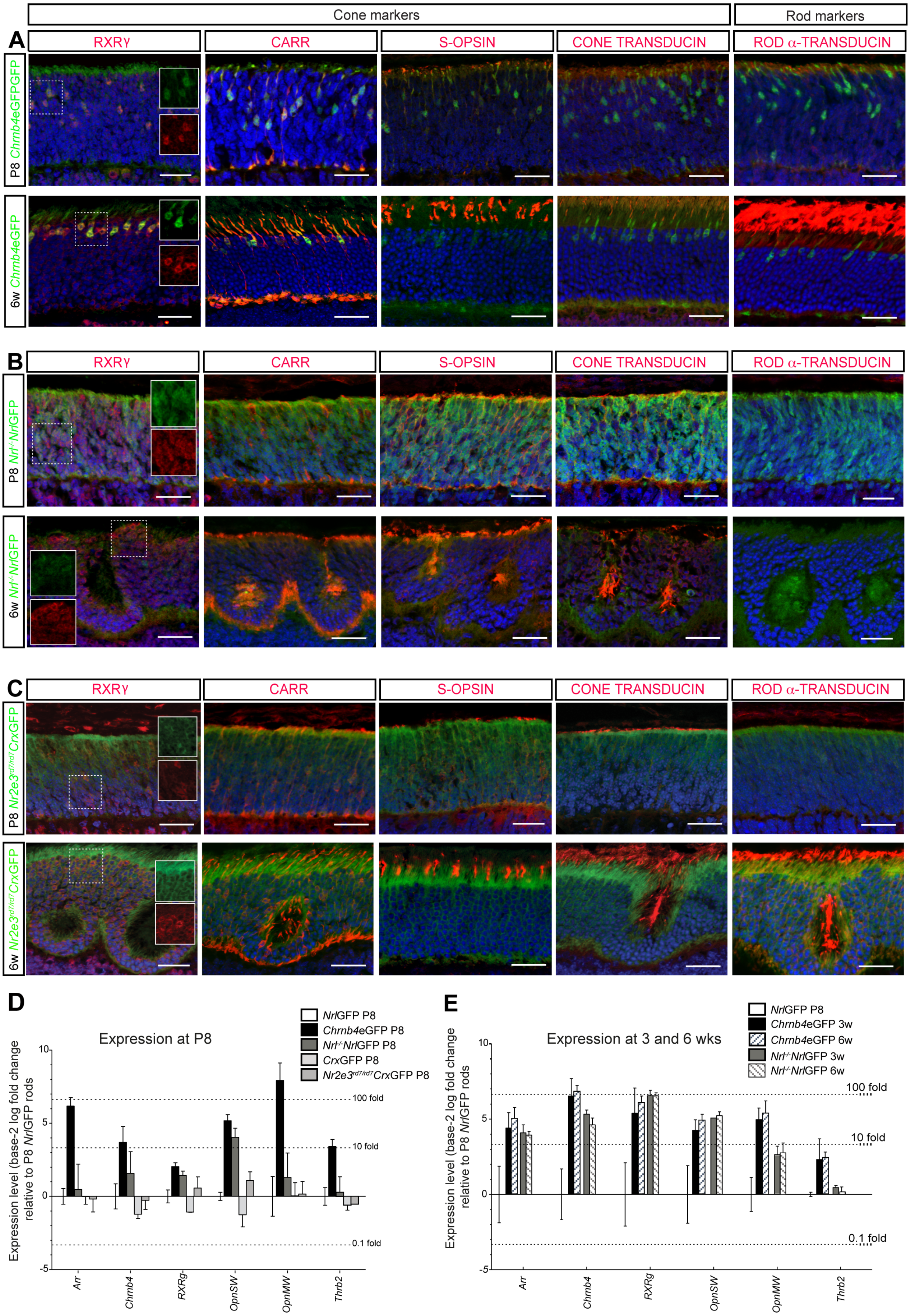
Cone markers
Rod markers
A
RXRγ
CARR
S-OPSIN
CONE TRANSDUCIN
ROD α-TRANSDUCIN
P8 Chrnb4eGFP
6w Chrnb4eGFP
B
P8 Nrl-/-NrlGFP
6w Nrl-/-NrlGFP
C
P8 Nr2e3rd7/rd7CrxGFP
6w Nr2e3rd7/rd7CrxGFP
D
Expression at P8
NrlGFP P8
Chrnb4eGFP P8
Nrl-/-NrlGFP P8
CrxGFP P8
Nr2e3rd7/rd7CrxGFP P8
Expression level (base-2 log fold change relative to P8 NrlGFP rods
100 fold
10 fold
0.1 fold
10
5
0
-5
Arr
Chrnb4
RXRg
OpnSW
OpnMW
Thrb2
E
Expression at 3 and 6 wks
NrlGFP P8
Chrnb4eGFP 3w
Chrnb4eGFP 6w
Nrl-/-NrlGFP 3w
Nrl-/-NrlGFP 6w
Expression level (base-2 log fold change relative to P8 NrlGFP rods
100 fold
10 fold
0.1 fold
10
5
0
-5
Arr
Chrnb4
RXRg
OpnSW
OpnMW
Thrb2

Figure S1. Immunohistological characterization of mouse lines used for donor cell generation, related to Figs. 1-3. Images show confocal projections of the central retina of postnatal day 8 and 6 wk (adult) old (**A**) *Chrnb4*eGFP, (**B**) *Nrl$^{-/-}$;NrlGFP,* and (**C**) *Nr2e3$^{rd7/rd7}$;Crx*GFP mice. Retinal sections were stained for the cone markers RXRγ, CONE ARRESTIN (CARR), S-OPSIN, CONE TRANSDUCIN (*Gnat2*) and the rod marker ROD α-TRANSDUCIN (*Gnat1*). Note that not all photoreceptor cells in the *Nrl$^{-/-}$;NrlGFP, and Nr2e3$^{rd7/rd7}$;CrxGFP* lines appear to express a full complement of cone markers in the adult. Scale bar 25μm. **D, E,** Quantitative PCR analysis of cone marker gene expression in GFP-expressing donor populations. (**D**) relative expression levels of cone genes in GFP+ cells isolated from the different donor retinae at P8, compared to P8 *Nrl*GFP retina cells. (**E**) changes in relative expression levels of cone genes in *Chrnb4e*GFP cones and *Nrl$^{-/-}$;NrlGFP* cone-like cells between 3 and 6 wks of age, compared to *Nrl*GFP rod precursor cells. Adult cells were taken from mice between 5.5 - 9 weeks old. Values given are -ΔΔCt, the difference in signal detection found for the gene and the housekeeping gene β-actin, compared to that in *Nrl*GFP cells of similar age, which was set to zero. This corresponds to the base-2 log of the fold change. Measurements were taken as the means of duplicates and the plotted values are +/- S.D. Statistical analysis is shown in **Supplemental Tables 1-4**.

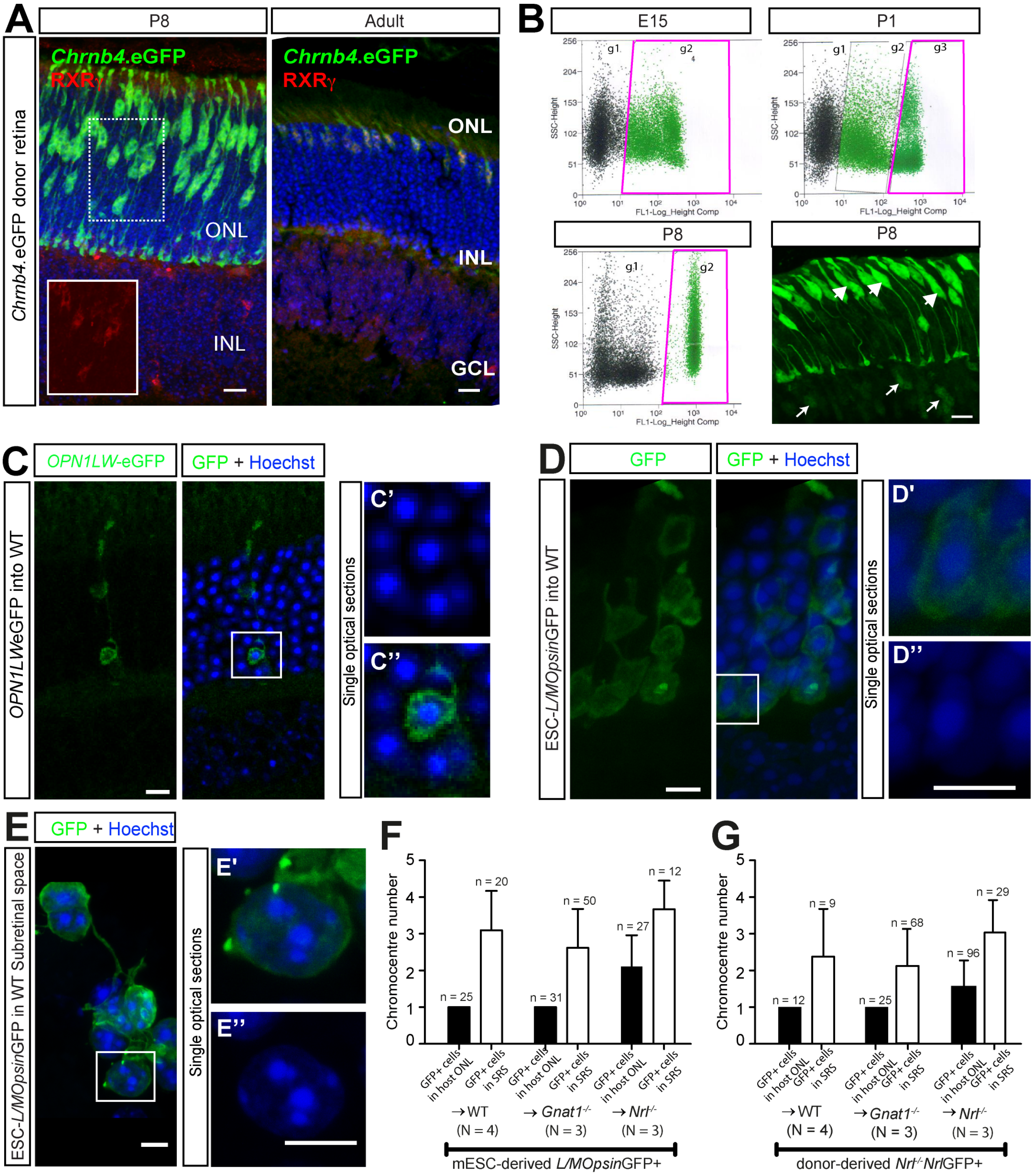

A
P8
Adult
Chrnb4.eGFP donor retina
Chrnb4.eGFP
RXRγ
ONL
INL
GCL
B
E15
P1
P8
g1
g2
g3
SSC-Height
FL1-Log_Height Comp
C
OPN1LW-eGFP
GFP + Hoechst
OPN1LWeGFP into WT
Single optical sections
C'
C''
D
GFP
GFP + Hoechst
ESC-L/MOpsinGFP into WT
Single optical sections
D'
D''
E
GFP + Hoechst
ESC-L/MOpsinGFP in WT Subretinal space
Single optical sections
E'
E''
F
Chromocentre number
n = 25
n = 20
n = 31
n = 50
n = 27
n = 12
GFP+ cells in host ONL
GFP+ cells in SRS
→WT (N = 4)
→ Gnat1-/- (N = 3)
→ Nrl-/- (N = 3)
mESC-derived L/MOpsinGFP+
G
n = 12
n = 9
n = 25
n = 68
n = 96
n = 29
donor-derived Nrl-/-NrlGFP+

Figure S2. Transplantation of donor- and mESC-derived GFP+ cone precursors leads to the presence of small numbers of predominantly rod-like cells within the host ONL, related to Fig. 1. (A) confocal images of P8 and adult *Chrnb4*-eGFP retinal sections co-stained with the cone and ganglion cell marker Rxr- γ. The $[GFP]_{high}$ cells become restricted to the apical margin of the ONL and are RXRγ+. $[GFP]_{low}$ cells were predominantly found in the inner retina. (**B**) FACS plots show the presence of different populations of GFP+ cells within the *Chrnb4*-EGFP+ population during embryonic and early postnatal development. Magenta boxes denote the population collected for transplantation at each age. **C-D**) confocal images of rod-like GFP+ cells within host ONL after transplantation of (**C**) *OPN1LW*-eGFP+ donor-derived cone photoreceptors and (**D**) mESC-derived *L/MOpsin*-GFP+ cone precursors. (**E**) confocal image of mESC-derived GFP+ cells within host subretinal space after transplantation. (**F-G**) chromocentre counts for GFP+ cells within the ONL and subretinal space of wildtype, *Gnat1*$^{-/-}$ and *Nrl*$^{-/-}$ hosts following transplantation of, (**F**) mESC-derived or (**G**) *Nrl*$^{-/-}$*Nrl*GFP donor-derived cone precursors. N = no. of eyes; n = no. of cells. Stats not applied. Scale bars 10 μm (**B, C, G**) and 5 μm (**E-F**).

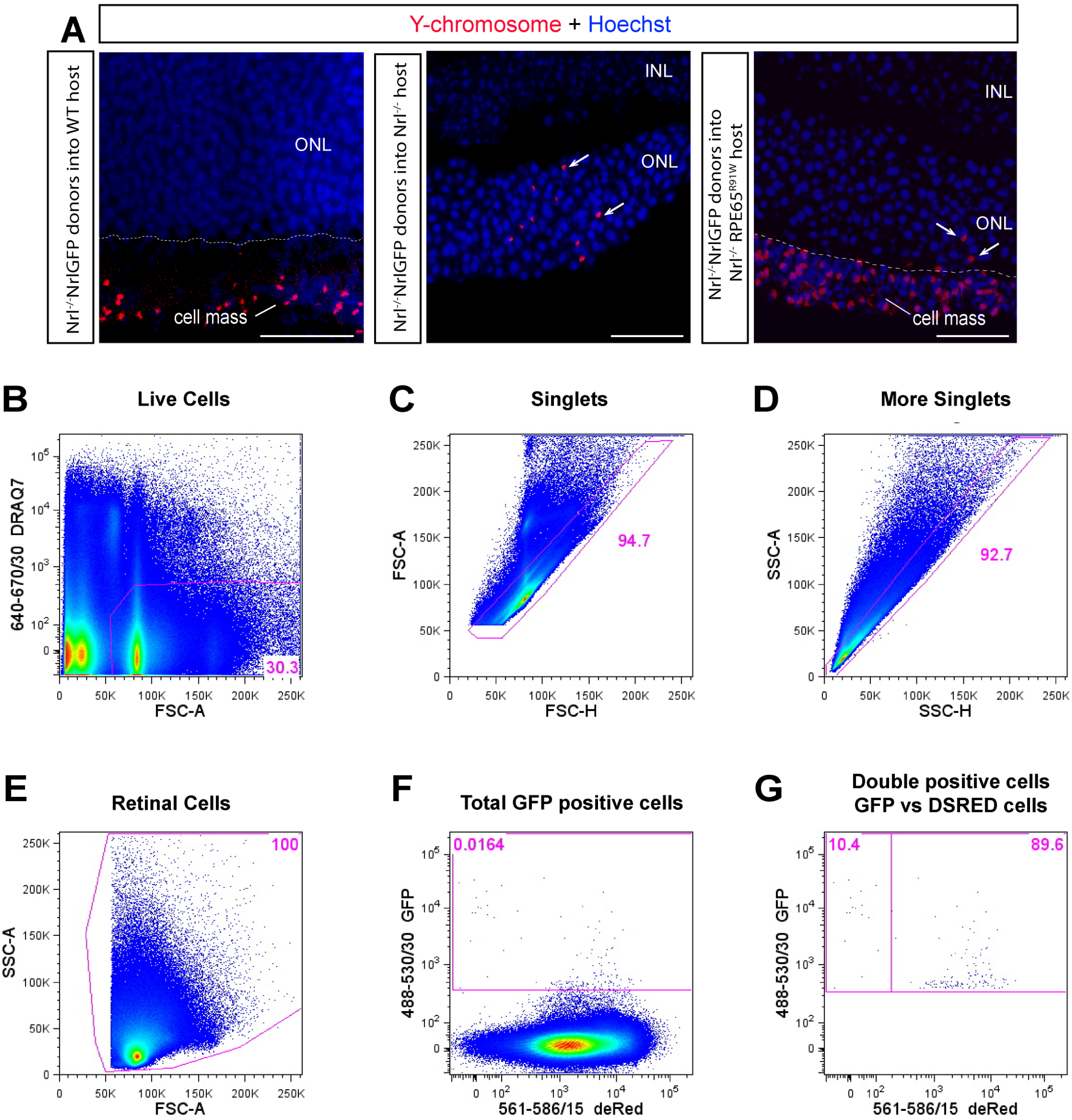
A
Y-chromosome + Hoechst
Nrl-/-NrlGFP donors into WT host
ONL
cell mass
Nrl-/-NrlGFP donors into Nrl-/- host
INL
ONL
Nrl-/-NrlGFP donors into Nrl-/- RPE65R91W host
INL
ONL
cell mass
B
Live Cells
640-670/30 DRAQ7
FSC-A
30.3
C
Singlets
FSC-A
FSC-H
94.7
D
More Singlets
SSC-A
SSC-H
92.7
E
Retinal Cells
SSC-A
FSC-A
100
F
Total GFP positive cells
488-530/30 GFP
561-586/15 deRed
0.0164
G
Double positive cells
GFP vs DSRED cells
488-530/30 GFP
561-586/15 deRed
10.4
89.6

Figure S3. FISH and Flow cytometry analysis of integration and material transfer, related to Fig. 3 and 5 and Supplemental Methods. A, Cone photoreceptors can integrate in some models of retinal degeneration. Confocal images of FISH labelling, showing significant numbers of Y-chromosome+ (*red*) nuclei in *Nrl*$^{-/-}$ host ONL but few, if any, in wildtype or *Nrl*$^{-/-}$*;RPE65*$^{R91W/R91W}$ host ONL, after transplantation of P8 *Nrl*$^{-/-}$*;Nrl*GFP donors. Cell masses located in SRS also demonstrate widespread labelling. **B-G,** Flow cytometry gating strategy for analysis of host (*DsRed* or *Nrl*$^{-/-}$*;DsRed*) and donor-derived (*Nrl*$^{-/-}$*;NrlGFP*) reporter proteins following transplantation. This example displays a *DsRed* host retina examined by flow cytometry 2-3 wks post-transplantation of *Nrl*$^{-/-}$*;NrlGFP* post-mitotic cone-like donor cells. (**B**) representative plot showing a dissociated retina stained for SYTOX Blue Dead Cell Stain™ used to exclude dead cells and cellular debris and identify the live cell population. (**C-D**) plots showing the exclusion of cellular aggregates to isolate single cells. (**E**) a representative plot depicting the relative size and granularity of single, live retinal cells. (**F-G)** plots of a representative transplanted sample showing (**F**) total GFP^{+} cells (*pink box*) and (**G**) subsequent gating showing the % of GFP^{+}-only (*left pink box*) and GFP^{+}/DsRed^{+} (*right pink box*). Gates were set based on controls, as presented in **Fig. 3** in the main text. Abbreviation: MFI (mean fluorescent intensity).

Supplemental Tables 1-4. qPCR comparisons of the expression of various cone and rod photoreceptor-specific marker genes in developing (P8) and Adult FACS purified GFP+ cells. Cell sources chosen for comparison are cone photoreceptors from *Chrnb4*eGFP retinas, cone like photoreceptors from *Nrl*$^{-/-}$*NrlGFP*, a mixed photoreceptor population from *Crx*GFP and a mixed photoreceptor population with increased numbers of cone like cells from *Nr2e3*$^{rd7/rd7}$*Crx*GFP. For *Chrnb4*eGFP and *Nrl*$^{-/-}$*NrlGFP* cells, 3 wk old cells were also obtained and compared with Adult NrlGFP cells. Groupwise and individual comparisons can be found in the tables below. Separate comparisons with *Nrl*GFP cells were made with *Chrnb4*eGFP, and with *Nrl*$^{-/-}$*NrlGFP* cells. *Nr2e3*$^{rd7/rd7}$*Crx*GFP cells were compared with *Crx*GFP cells as a closer control. Tables show the results of two-way ANOVAs, the effects of Cell type, Gene, and the interaction between the two, and post-test comparisons. Statistical comparisons are made using the base-2 log of the fold change of expression, as shown in **Supplemental Fig. S1D, E**.

**Chrnb4.eGFP - P8**	**Cone markers**					
	Effects					
	Cell		Gene		Interaction	
	F(1,54)	p	F(5,54)	p	F(5,54)	p
	636.62	<0.0001 ****	21.39	<0.0001 ****	21.39	<0.0001 ****
	Post-test comparisons (Bonferroni-corrected) – significance with p values					
	Arr	*Chrnb4*	*Rxrγ*	*OpnSW*	*OpnMW*	*Thrβ2*
	<0.0001	<0.0001	<0.001	<0.0001	<0.0001	<0.0001
**Chrnb4.eGFP - 3 weeks and Adult**	**Cone markers**					
	Effects					
	Cell		Gene		Interaction	
	F(2,36)	p	F(5,36)	p	F(10,36)	p
	100.46	<0.0001 ****	5.83	0.0005 ****	1.49	0.184 n.s.
	Post-test comparisons (Bonferroni-corrected) – significance with p values					
	Arr	*Chrnb4*	*Rxrγ*	*OpnSW*	*OpnMW*	*Thrβ2*
	3 wks: <0.001	<0.0001	<0.0001	<0.01<0.001	<0.001	n.s.
	6 wks: <0.001	<0.0001	<0.0001	<0.001	<0.0001	n.s.

Table S1. qPCR comparisons of cone marker expression between (top) P8 Chrnb4.eGFP and P8 NrlGFP flow sorted cells and (bottom) between 3 wk and adult Chrnb4.eGFP and adult NrlGFP flow sorted cells

<table>
<tr><td rowspan="10">Chrnb4.eGFP - P8</td><td colspan="6">Rod markers</td></tr>
<tr><td colspan="6">Effects</td></tr>
<tr><td colspan="2">Cell</td><td colspan="2">Gene</td><td colspan="2">Interaction</td></tr>
<tr><td>F(1,27)</td><td>p</td><td>F(2,27)</td><td>p</td><td>F(2,27)</td><td>p</td></tr>
<tr><td>13</td><td>0.0012
**</td><td>0.06</td><td>0.9384
n.s</td><td>0.07</td><td>0.936
n.s.</td></tr>
<tr><td colspan="6"></td></tr>
<tr><td colspan="6">Post-test comparisons (Bonferroni-corrected) – significance with p values</td></tr>
<tr><td colspan="2">Nrl</td><td colspan="2">Nr2e3</td><td colspan="2">Rhodopsin</td></tr>
<tr><td colspan="2">n.s</td><td colspan="2">n.s</td><td colspan="2">n.s</td></tr>
<tr><td colspan="6"></td></tr>
<tr><td rowspan="10">Chrnb4.eGFP - 3 weeks and Adult</td><td colspan="6">Rod markers</td></tr>
<tr><td colspan="6">Effects</td></tr>
<tr><td colspan="2">Cell</td><td colspan="2">Gene</td><td colspan="2">Interaction</td></tr>
<tr><td>F(2,18)</td><td>p</td><td>F(2,18)</td><td>p</td><td>F(2,18)</td><td>p</td></tr>
<tr><td>48.11</td><td><0.0001
****</td><td>0.27</td><td>0.7694
n.s.</td><td>0.13</td><td>0.9679
n.s.</td></tr>
<tr><td colspan="6"></td></tr>
<tr><td colspan="6">Post-test comparisons (Bonferroni-corrected) – significance with p values</td></tr>
<tr><td colspan="2">Nrl</td><td colspan="2">Nr2e3</td><td colspan="2">Rhodopsin</td></tr>
<tr><td colspan="2">3 wks: n.s</td><td colspan="2">n.s</td><td colspan="2"><0.05</td></tr>
<tr><td colspan="2">6 wks: <0.001</td><td colspan="2"><0.0001</td><td colspan="2"><0.001</td></tr>
</table>

Table S2. qPCR comparisons of rod marker expression (top) between P8 *Chrnb4*-eGFP and P8 *Nrl*GFP flow sorted cells and (bottom) between 3 wk and adult *Chrnb4*-eGFP and adult *Nrl*GFP flow sorted cells

<table>
<tr><td rowspan="9">Nrl$^{-/-}$NrlGFP - P8</td><td colspan="6">Cone markers</td></tr>
<tr><td colspan="6">Effects</td></tr>
<tr><td colspan="2">Cell</td><td colspan="2">Gene</td><td colspan="2">Interaction</td></tr>
<tr><td>F(1,42)</td><td>p</td><td>F(5,42)</td><td>p</td><td>F(5,42)</td><td>P</td></tr>
<tr><td>30.37</td><td><0.0001</td><td>3.97</td><td><0.0049</td><td>3.97</td><td>0.0049</td></tr>
<tr><td colspan="6"></td></tr>
<tr><td colspan="6">Post-test comparisons (Bonferroni-corrected) – significance with p values</td></tr>
<tr><td>Arr</td><td>Chrnb4</td><td>Rxrγ</td><td>OpnSW</td><td>OpnMW</td><td>Thrβ2</td></tr>
<tr><td>n.s</td><td>n.s</td><td>n.s</td><td><0.0001</td><td>n.s</td><td>n.s</td></tr>
<tr><td rowspan="10">Nrl$^{-/-}$NrlGFP – 3 weeks and adult</td><td colspan="6">Cone markers</td></tr>
<tr><td colspan="6">Effects</td></tr>
<tr><td colspan="2">Cell</td><td colspan="2">Gene</td><td colspan="2">Interaction</td></tr>
<tr><td>F(2,36)</td><td>p</td><td>F(5,36)</td><td>p</td><td>F(10,36)</td><td>p</td></tr>
<tr><td>95.81</td><td><0.0001
****</td><td>19.67</td><td><0.0001
****</td><td>5</td><td>0.0002
***</td></tr>
<tr><td colspan="6"></td></tr>
<tr><td colspan="6">Post-test comparisons (Bonferroni-corrected) – significance with p values</td></tr>
<tr><td>Arr</td><td>Chrnb4</td><td>Rxrγ</td><td>OpnSW</td><td>OpnMW</td><td>Thrβ2</td></tr>
<tr><td>3 wks:
<0.001</td><td><0.0001</td><td><0.0001</td><td><0.0001</td><td><0.05</td><td>n.s.</td></tr>
<tr><td>6 wks:
<0.001</td><td><0.0001</td><td><0.0001</td><td><0.0001</td><td><0.05</td><td>n.s.</td></tr>
</table>

Table S3. qPCR comparisons of cone marker expression (top) between P8 *Nrl*$^{-/-}$*Nrl*GFP and P8 *Nrl*GFP flow sorted cells and (bottom) between 3 wk and adult *Nrl*$^{-/-}$*Nrl*GFP and adult *Nrl*GFP flow sorted cells.

**Nr2e3$^{rd7/rd7}$CrxGFP - P8**	**Cone markers**					
	Effects					
	Cell		Gene		Interaction	
	F(1,24)	p	F(5,24)	p	F(5,24)	p
	4.81	0.0382 *	0.49	0.7837 n.s	1.18	0.3494 n.s
	Post-test comparisons (Bonferroni-corrected) – significance with p values					
	Arr	Chrnb4	Rxrγ	OpnSW	OpnMW	Thrβ2
	n.s	n.s	n.s	n.s	n.s	n.s
**Nr2e3rd7/rd7CrxGFP - Adult**	**Cone markers**					
	Effects					
	Cell		Gene		Interaction	
	F(1,24)	p	F(5,24)	p	F(5,24)	p
	10.64	0.0033 **	11.05	<0.0001 ****	0.85	0.5271 n.s.
	Post-test comparisons (Bonferroni-corrected) – significance with p values					
	Arr	*Chrnb4*	*Rxrγ*	*OpnSW*	*OpnMW*	*Thrβ2*
	6 wks: n.s.	n.s.	n.s.	n.s.	n.s.	n.s.

Table S4. qPCR comparisons of cone marker expression (top) between P8 *Nr2e3*$^{rd7/rd7}$*Crx*GFP and P8 *Crx*GFP flow sorted cells and (bottom) between adult *Nr2e3*$^{rd7/rd7}$*Crx*GFP and adult *Crx*GFP flow sorted cells.

Mouse model	Donor/ Recipient	Key features	Degeneration	Age at time of use
*Nrl*GFP	Donor	Normal retina, all rods are GFP+	None	E15.5 - P8
$Nrl^{-/-}$ *Nrl*GFP	Donor	Cone-like ('cod') only retina. All cone-like cells are GFP+, true cones are GFP-	Mild	E15.5 - P8
$Nr2e3^{rd7/rd7}$ *Crx*GFP	Donor	Normal cones and supra-normal numbers of S cone-like hybrid cells. All PRs are GFP+	Mild, late	E15.5 - P8
*Chrnb4*GFP	Donor	Normal retina. All cones are GFP+ (RGCs also weakly GFP+)	None	E15.5 - P8
Wildtype (*C57Bl/6*)	Recipient	Normal retina	None	2 - 3 mnth
$Prph2^{rds/rds}$	Recipient	Inherited RP model. Moderate rate of degeneration - 50% loss of rods by 8 wks. Secondary loss of cones	Moderate	2 - 3 mnth
$Nrl^{-/-}$	Recipient	Cone-like ('cod') only retina. Rosettes, mild degeneration completed by ~6wks	Mild	2 - 3 mnth
$Nr2e3^{rd7/rd7}$	Recipient	Cone-only retina comprising mix of cods and supra-normal number of S cones. Mild degeneration at very late stages	Mild, late	2 - 4 mnth
cpfl5	Recipient	Missense mutation in CNGA3 gene. Cones are non-functional and degenerate over a period of 2-5 months. Loss of S cones precedes M/L cones	Moderate (cones only)	2 - 4 mnth
cpfl1	Recipient	$PDE6c^{cpfl1}$ spontaneous mutation causing frame shift and premature stop in PDE6c. Non-functional cones, which degenerate over a period of 5 months	Moderate (cones only)	2 - 4 mnth

Table S5. Key features of the donor and recipient models and the ages at time of use.

Primary antibody Target	Animal	Source	Concentration	Block	Secondary antibody (AlexaFluor)
RXRγ	Rabbit	Abcam (ab15518)	1:200	10% FBS<br>1% BSA<br>0.1% Triton	Goat anti-rabbit 546
Biotinylated Peanut agglutinin (PNA)	N/A (plant lectin)	Vector Labs (L-1070)	1:500	10% FBS<br>1% BSA<br>0.1% Triton	Streptavidin 633
S-OPSIN (Opn1sw)	Goat	Santa Cruz (sc-14365)	1:100	2% NDS<br>1% BSA<br>0.1% Triton	Donkey anti-goat 546
PERIPHERIN2	Rabbit	Gift from G.Evans	1:500	10% FBS<br>1% BSA<br>0.1% Triton	Goat anti-rabbit 546
ROD α-TRANSDUCIN	Rabbit	Santa Cruz (sc389)	1:500	2% NDS<br>1% BSA<br>0.1% Triton	Donkey anti-goat 546
CONE ARRESTIN (CARR)	Rabbit	Millipore (ab15282)	1:200	2% NDS<br>1% BSA<br>0.1% Triton	Donkey anti-rabbit 647
PROTEIN KINASE C-α	Mouse	Millipore (05-983)	1:500	10% NGS<br>1% BSA<br>0.1% Triton	Goat anti-mouse 546
GFP.FITC	Goat	Abcam (ab6662)	1:200	10% FBS<br>1% BSA<br>0.1% Triton	N/A

Table S6. Antibodies and blocking conditions used for immunohistochemistry. FBS – foetal bovine serum; BSA- Bovine Serum Albumin; NDS – Normal Donkey Serum; NGS – Normal Goat Serum

Gene	F primer (5' to 3')	R primer (5' to 3')	Amplicon (bp)	Probe number
Arr3	GCTAACCTGCC CTGTTCAGTA	TTCAAAGTCAACC CCACAGG	75	64
Chrnb4	GCTCCTCGTCTC TCTGTTCG	CATCCATCAGCTT CTCCTCTG	77	64
En2	GACCGGCCTTC TTCAGGT	CCTGTTGGTCTGA AACTCAGC	132	110
Fgfr4	AATCGTATTGG AGGCATTCG	TCCGAGGGTACCA CACTTTC	71	47
Gnat2	CATGTCCACAC TAGGCATTGAC	GCCAGGTTGTTGA GCTGTCT	72	21
Opn1mw (M-opsin)	ATCGTGCTCTGC TACCTCCA	TTTCTGTTGCTTTG CCACTG	60	5
Pde6c	CGAAGGTGAAG GTGACTGAAG	GCTGCCTCCTAAA TCTGTGG	86	4
Rxrγ	CAGAAGTGCCT GGTCATGG	CCTCACTCTCTGCT CGCTCT	82	82
Opn1sw (S-opsin)	CAACCCCATCA TCTACTGCTT	GACACGTCAGATT CGTCTGC	96	4
Thrβ2	ATGCATCTATGT TGGCATGG	GCTTGGCTAGCCT CTTGCT	62	42
Nrl	TTCTGGTTCTGAC AGTGACTACG	TGGGACTGAGCAGA GAGAGG	77	53
Nr2e3	CAGCCAGCCTG TGAGGTT	AGAAGCTCAATGC GCTCAG	81	32
Rhodopsin	ACCTGGATCAT GGCGTTG	TGCCCTCAGGGAT GTACC	70	32
β-actin	AAGGCCAACCG TGAAAAGAT	ACCAGAGGCATAC	110	56

Table S7. Forward and Reverse primer sequences used for qRT-PCR

Supplemental Experimental Procedures

Ethics statement

All animal studies were carried out under the Animals (Scientific Procedures) Act 1986 under a project license PPL 70/8120 issued by the UK Government Home Office and conducted in accordance with protocols approved by the Animal Welfare and Ethics Committee of the UCL Institute of Ophthalmology. All animals were killed by cervical dislocation performed by trained personnel (approved under Schedule 1 as a method of humane killing). All efforts were made to minimize the number and suffering of animals used in these experiments.

Animals

Male and Female mice were group housed in the animal facility in the Institute of Ophthalmology at University College London on a standard 12-hour light/dark cycle at the same light levels throughout the experimental period. Animals were kept in individually ventilated cages on animal grade wood chip and given access to nesting material and food and water ad lib.

The following lines were used: *OPN1LW*-eGFP (Fei and Hughes, 2001) (Mutant Mouse Regional Resource Centre, MMRRC); *Chrnb4-e*GFP (Siegert et al., 2009) (MMRRC; Supplemental Fig. S2A. N.B. this model is usually homozygous for the *Pde6β rd1)* mutation, which causes rapid retinal degeneration. Mice were backcrossed with *C57Bl/6* to remove this mutation from the line); *Nr2e3*^{rd7rd7} (Haider et al., 2000), *Nrl*-GFP(Akimoto et al., 2006) and *Nrl*$^{-/-}$ (Mears et al., 2001) (gifts from A. Swaroop, National Eye Institute, Bethesda, Maryland); *Crx*-GFP(Samson et al., 2009) (gift from C. Cepko, Harvard, USA) *Nr2e3*$^{rd7/rd7}$*Crx*-GFP (bred on site from the above lines; Supplemental Fig. S1B; *Nrl*$^{-/-}$*;Nrl*-GFP$^{+/+}$ (bred on site from the above lines; Supplemental Fig. S2C; *Pde6c*$^{cpfl1/cpfl1}$ (Chang et al., 2002) (gift from X-Q Ding, University of Oklahoma); *Prph2*$^{rd2/rd2}$ (cba.rds) (Sanyal et al., 1980) (gift from D. Bok, Jules Stein Institute, Los Angeles); *Cnga3*^{cpfl5} (Pang et al., 2010) (gift from X-Q Ding, University of Oklahoma); *Nrl*$^{-/-}$*;RPE*$^{R91W/R91W}$ (Samardzija et al., 2014) (gift from C. Grimm, University of Zurich, Switzerland); B6.Cg-Tg(CAG-DsRed*MST)1Nagy/J ("*DsRed*") (Jackson Labs); *Nrl*$^{-/-}$;DsRed (bred on site from above) and Wildtype *C57Bl/6J* – Harlan Laboratories. See **Supplemental Table S5** for summary of key features and ages used for all donor and host lines.

FACS isolation of donor cells

The neural retinae of donor *Chrnb4-e*GFP, *Nrl*$^{-/-}$*;Nrl*-GFP$^{+/+}$ and *Nr2e3*$^{rd7/rd7}$*;Crx*-GFP$^{+/+}$ mice were isolated by dissection. Unless otherwise stated, the donor ages used (+/- 1.5 days) were embryonic day (E) 15.5, postnatal day (P) 1 and P8. A single-cell suspension was obtained using papain-based digestion (Worthington Biochemical, Lorne Laboratories, UK) per the manufacturer's instructions. Fluorescent Activated Cell Sorting (FACS) was used to purify the GFP expressing population utilizing a special order 5-laser BD Influx™ Cell Sorter (BD Biosciences) equipped with a 200mW 488nm blue laser to excite GFP. GFP positive cells were collected using the 488-530/40nm detector. A 70µm nozzle at 50 psi was used for sorting and cells were collected into a 1:1 FBS/ EBSS solution. Sorted GFP^{+ve} cells were re-suspended at 200,000 live cells / µl (as assessed using a Scepter hand-held cell counter; Millipore, UK) in sterile EBSS and DNase (0.05%) and kept on ice prior to transplantation. N.B. Due to the very low cellular yield, *Chrnb4-e*GFP and *OPN1LW*-eGFP donor cells were prepared at a concentration of 100,000 live cells per µl.

Preparation of donor cells from mouse ESCs

A mouse EK.CCE ES cell line (Evans and Kaufman, 1981) (129/SvEv; gift of Professor E. Robertson) was maintained in GMEM containing 10% Knockout Serum Replacement (KSR), 1% Fetal Bovine Serum (FBS), 0.1mM NEAA, 1mM pyruvate, 0.1mM 2-mercaptoethanol with 2000U/ml LIF, 0.5µM MEK inhibitor (PD0325901) and 1.5 µM GSK3 inhibitor (CHR99021)(Gonzalez-Cordero et al., 2013). Briefly, for 3D retinal differentiation, $3x10^4$ dissociated ES cells were re-suspended per millilitre of differentiation medium (GMEM containing 1.5% KSR, 0.1mM NEAA, 1mM pyruvate, 0.1mM 2-mercaptoethanol), plated into 96 well low-binding plates and incubated at 37°C, 5% CO_2. Embryoid body cell aggregates (EBs) formed within 24 hrs, on day 1 of culture, growth factor reduced Matrigel™ (GIBCO) was added to each well to give a final concentration of 2%. For whole EB retinal differentiation towards photoreceptor cell fate, EBs were transferred into retinal maturation medium (DMEM/F12 Glutamax media containing N2 supplement and Pen/strep, herein Retinal Maintenance Media, RMM) at day 9, plated in low-binding plates at a density of 6 EBs/cm^2 and incubated at 37°C, 5% CO_2. The media was changed every 2-3 days, with the addition of 1mM Taurine and 500nM retinoic acid from day 14 of culture onwards. EBs were labelled with AAV ShH10.L/MOpsin.GFP

(Kruczek et al., 2017) on day 20 of culture and harvested on d26-30. This encompasses 3 changes of media between viral administration and cell preparation.

Note that the time-course of differentiation can be heterogeneous both within a given EB and between EBs even in the same dish. This may account for the higher inter-animal variation between transplantations using mESCs-derived cones than between donor-derived cones, which tend to be more developmentally synchronized.

FACS isolation of mouse ESC-derived photoreceptors

For transplantation, EBs were dissociated at day 26-30 of culture into a single cell suspension using a modified protocol using reagents from a papain-based Neurosphere Dissociation Kit (Miltenyi Biotec, 130-095-943). Briefly, samples were incubated in a papain-based enzyme dissociation mix (Miltenyl Biotech) at 37°C for 15 min, gently triturated and then spun down at 320g for 7 minutes at room temperature. Cell pellets were re-suspended in HBSS (with 0.5mM MgCl2) with FCS (1%), 66% 25mM HEPES and DNase solution (1%) and passed through a 35 micron cell strainer at a concentration of ~20 million cells/mL. Cells were FACS sorted for GFP fluorescence using a special order 5-laser BD Influx Cell Sorter^{TM} (BD Biosciences) equipped with a 200mW 488nm blue laser to excite GFP. GFP positive cells were collected using a 488-530/40nm detector. A 70µm nozzle at 50 psi was used and cells were collected into a 20% FBS in RMM solution. Sorted GFP^{+ve} cells were re-suspended at 200, 000 live cells / µL in sterile HBSS (+Ca^{2+}, Mg^{2+}) and DNase solution (3%) and kept on ice prior to injection. The final supernatant was also retained for injection as a control for viral carry-over (Pearson et al., 2016, West et al., 2012, Gonzalez-Cordero et al., 2013).

Donor cell transplantation

Donor cells were introduced into the subretinal space of the superior (dorsal) retina by a single injection of 1µl. Injection protocols were as described in previous publications (Pearson et al., 2016, Pearson et al., 2012). Briefly, intraperitoneal injections of an anaesthetic mixture containing Dormitor, ketamine and sterile water in a 5:4:42 ratio were used before injections (0.01 ml per gram). Pupils were dilated using 1% topical tropicamide. 1µl injections were made slowly by a single surgeon with a sterile Hamilton syringe at an oblique angle through the superior sclera into the subretinal space to produce visible retinal detachment in the superior retina.

Introducing a small parathentesis in the anterior chamber prior to cell injection relieved the associated increases in intraocular pressure. Both eyes received topical treatment of chloramphenicol and viscotears. Anaesthesia was reversed using intraperitoneal injections of 0.1 mg/ml of Antisedan (Pfizer, Kent UK).

Recipients of transplanted cells were typically sacrificed between 2 and 3 wks post injection and the eyes removed and placed in 1% paraformaldehyde (PFA) and the corneas and lens carefully removed. Eyes were kept in 1% PFA for 20 – 30 minutes before being washed in PBS, cryoprotected with 20% sucrose in PBS solution and cryo-embedded in OCT (Tissue-Tek). Cryosections 18 μm thick were cut in the coronal plane. All sections were collected for analysis.

Analysis of cell counts in transplanted eyes by fluorescence microscopy

After immunostaining and enhancing GFP signal with 1:200 FITC conjugated anti-GFP goat polyclonal antibody (Abcam, UK), sections from injected eyes were viewed with a fluorescence microscope (Observer Z.1, Zeiss). Every third cryosection was viewed and all GFP+ cells with cell bodies located in the ONL were counted and assessed, when possible, for co-staining and morphology. Cells were counted if the whole cell body was correctly located within the ONL and at least one of the following was visible; spherule synapse, inner/outer processes, inner/outer-segments. The total number of cells for each injected eye was calculated as three times this count. Where relevant, counts were made in a blinded fashion, whereby the assessor was unaware of the donor cell source/recipient genotype for any given eye. Cell counts for individual eyes were excluded from the analysis: if donor cells were present in the vitreous, indicative of accidental intravitreal transplantation of the cells; if there was no cell mass present in the subretinal space, indicative of reflux at time of injection; and/or there was significant macrophage infiltration and evidence of level II/III rejection, as defined in (West et al., 2010) (but see Results for details regarding specific models).

Analysis of cell counts in transplanted eyes by flow cytometry

Transplanted retinae were taken 2-3 wks post-transplantation. Neural retinae were isolated by dissection and any overlying cell mass carefully removed using direct visualisation under a fluorescence microscope. Individual neural retinae were dissociated using the papain-based Neurosphere Dissociation Kit, described above. An aliquot from each sample was taken to determine absolute cell counts using the Beckman Coulter

Vi-Cell XR Cell Viability Analyser (Beckman Coulter). The remaining media was spun down at 320g for 7 minutes at 4°C, aspirated and then re-suspended in 350 µL of DMEM^{+} media with 1.5 µM of SYTOX™ Blue Dead Cell Stain (ThermoFischer Scientific) for sample acquisition on the flow cytometer. All samples were acquired using a BD LSRFortessa™ X-20 flow cytometer (BD Biosciences), equipped with 5 lasers (i.e. 355nm, 405nm, 488nnm, 561nm & 640nm lasers). The entire sample was acquired for analysis due to the low total frequency of transplanted donor cells. Fluorescence gates were defined by using *NrlGFP* and *DsRed* (or *Nrl*$^{-/-}$*;DsRed*, where appropriate), and wild type retinae as positive and negative controls, respectively. Cellular analysis of the samples included a gating hierarchy whereby dead cells, cellular debris and cellular aggregates were excluded prior to the analysis of single, live cells, as shown in **Supplemental Fig. S3B-G**. It is important to note that while every effort is made to remove any remaining donor cell mass prior to dissociation, it is not possible to be completely certain of this. Therefore, it is possible that some GFP^{+}-only cells reflect donor cells within the SRS. We suggest that for these reasons, this method of assessment provides positive demonstration of CMT but does not provide an accurate quantification of the number of integration events. In this regard, FISH analysis is likely to be more accurate.

Immunohistochemistry and Histology

Eyes were dissected out after administering a small burn to the overlying sclera to provide a landmark for the superior retina. The eye cups were then carefully orientated and embedded in a standardized fashion in OCT (TissueTek) before being left overnight (o/n) at -20°C and then cut as transverse sections 18 µm thick. To avoid oblique cuts, all images shown are from the central most region of the eye, immediately adjacent to or through the optic nerve. Where comparing across developmental time points, immunohistochemistry was performed at the same time for all time points for any given marker. Specific details for each antibody and protocols used for immunohistochemistry can be found in **Supplemental Table S6**. Cryosections were air-dried for 15 - 30 min and *washed* in phosphate-buffered saline (PBS). Sections were pre-blocked for 1 hr at room temperature (RT) in a blocking solution before being incubated with appropriate primary antibody o/n at 4°C. After rinsing with PBS, sections were incubated with secondary antibody for 2 hrs at RT, rinsed and counter-stained with Hoechst 33342. Negative controls omitted the primary antibody. Note that all images showing GFP^{+} cells have been counter-stained with an anti-GFP antibody.

Fluorescent In situ hybridization

Eye-cups were fixed for 1 h in 4% PFA and cryopreserved in OCT. Serial sections were cut (Note 10 μm thick, compared to 18 μm elsewhere) across 6 sets of slides and screened for GFP^+ cells. For Fluorescent In Situ Hybridization (FISH) treated with 0.2M HCl for 20 minutes, at RT, and 5 minutes wash in 2x SSC/0.05 Tween20. Followed by 2x SSC incubation 20 minutes, at 80°C. Enzyme digestion as preformed with 0.1mg/mL of Protease K solution for 10 minutes, at 37°C. After washed and dehydrated, slides were left to air dry. 10μL of mouse Y chromosome paint probe (Empire Genomics) was added to each 22x22 mm area and sealed. Following denaturation, for 10 minutes at 90°C, sections were incubated overnight at 42°C. After seal removal slides were placed in 2x SSC for 10 minutes at 42°C. After the PBS wash, slides were blocked in PSB/5%BSA/0.1%triton for 30 minutes at room temperature followed by overnight incubation, at 4°C, with anti-GFP FITC conjugated antibody in 1:100 dilution. After the PBS wash, slides were counterstained with DAPI (10 μM).

Although we were able to obtain some examples of GFP^+ signal in sections also processed for FISH (shown in **Fig. 5**), it was not possible to retain the GFP signal with sufficient reliability to obtain cell counts. To enable assessment of the number and morphology of GFP^+ cells and the number of Y-chromosome positive (Y^+) cells in each retina, serial sections were processed for either GFP or FISH. Blinded counts were made across the entire section and multiplied to give an estimate per eye. The total number of Y-chromosome+ nuclei was divided by the total number of GFP^+ cells to give a percentage of integration events versus CMT. Note that Y-chromosome staining was robust with ~85% of nuclei labelling with the Y-probe in control male retinal sections. However, since labelling was not 100%, this method of quantification is likely to yield a slight underestimate of the percentage of the GFP^+ cells that are additionally Y^+.

Image acquisition

Retinal sections were viewed on a confocal microscope (Leica TCS SPE, Leica Microsystems), typically using x40 oil-immersion objective. Hoechst 33342 was excited using a 400nm, GFP using a 488nm and Alexa546/DsRed using 543nm laser lines. Collection of the emission wavelengths were set according to Leica suggested pre-sets, adjusted to ensure minimal overlap of collected emission spectra. Series of XY optical sections (<1 μm thick) were taken at 1.0μm steps throughout the depth of the section and built into a stack to

give xyz projection image. LAS-AF image software (Leica) was used. Individual xy images were acquired using a 2-frame average and at 1024 x 1024 resolution.

Semi-thin sections and Electron Microscopy

Mice were sacrificed and the eyes removed after administering a small burn to the overlying sclera to provide a landmark for the superior retina. Eyes were fixed in 3% glutaraldehyde / 1% PFA buffered to pH 7.4 with 0.08 sodium cacodylate-HCl. The cornea and lens were removed and the eye-cups orientated and processed, as previously described (Pearson et al., 2012). Briefly, following a washing step (15 min; 2.5% glutaraldehyde and 0.1 m cacodylate buffered to pH 7.4), the eyes were osmicated for 2.5 hrs in a 1% aqueous solution of osmium tetroxide in the dark, followed by dehydration steps through ascending ethanol series (50 - 100%, 10 min per step with rotation). After three changes of 100% ethanol, specimens were passed through propylene oxide (3 x 10 min) and left in a 50:50 mixture of propylene oxide and araldite for a minimum of 3 hrs with rotation at RT. Following a single change to fresh araldite (5 hrs with rotation) the specimens were embedded and cured for 48 hrs at 60°C. Semi-thin (0.7 μm) and ultra-thin (0.07 μm) sections were cut using a Leica Ultracut S microtome fitted with an appropriate diamond knife (Diatome histoknife Jumbo or Diatome Ultra-thin respectively). Ultra-thin sections were collected on copper grids (100 mesh, Agar Scientific, UK), contrast-stained with 1 % uranyl acetate and lead citrate and analysed using a JEOL 1010 Transmission Electron Microscope (80 kV), fitted with a digital camera for image capture. Semi-thin sections were stained with 1% toluidine blue and evaluated using a Leitz Diaplan microscope fitted with a Leica digital camera DC 500 for image capture.

Real-time and RT-PCR analysis

RNA was extracted with RNeasy Micro/Mini Kit (QIAGEN) and reverse-transcribed using QuantiTect Reverse Transcription Kit (QIAGEN). The cDNA was amplified with gene-specific primers (see **Supplemental Table S7**). PCRs were conducted using at least 3 separate RNA preparations. Real-time quantitative RT-PCR was performed with a thermal cycler (7900HT; Applied Biosciences), as previously described. Reagents were obtained from Roche Diagnostics and primers were designed for specific probe-binding regions using the Roche Universal Probe Library. Samples were run in duplicate and at least 3 independent differentiation cultures were analysed.

Statistics and randomisation

All means are stated ± standard deviation, except cell counts which are stated as ± standard error of the mean. N = number of eyes examined, n = number of cells, where appropriate. For qualitative histological assessments, at least 3 eyes from independent animals per group were used. For cell counts, both eyes per animal received injections to reduce the total number of animals used but the order of animals (different models) was randomized to ensure distribution between beginning and end of any given injection session. Cell counts represent data from at least three independent donor cell preparation/transplantation sessions, wherever possible. Eyes presenting with evidence of moderate inflammation and signs of acute rejection (Grade 2/3, as described by (West et al., 2010, Warre-Cornish et al., 2013)) were omitted from the cell counts but the numbers are shown as a proportion of the total number injected e.g. N = X successful transplants meeting minimum acceptance criteria/Y total number of eyes injected.

Statistical significance was assessed using a one-way ANOVA test with Dunnett's (comparing against wild-type) or Tukey's or Bonferroni's (inter-group comparisons) correction applied for multiple comparisons, as appropriate. P values are presented as $p < 0.05$ = *, $p < 0.01$ = ** and $p < 0.001$ = ***.

Data Availability

The data sets generated during and/or analysed during the current study are available from the corresponding author on reasonable request

References for Supplemental Information

AKIMOTO, M., CHENG, H., ZHU, D., BRZEZINSKI, J. A., KHANNA, R., FILIPPOVA, E., OH, E. C., JING, Y., LINARES, J. L., BROOKS, M., ZAREPARSI, S., MEARS, A. J., HERO, A., GLASER, T. & SWAROOP, A. 2006. Targeting of GFP to newborn rods by Nrl promoter and temporal expression profiling of flow-sorted photoreceptors. *Proc Natl Acad Sci U S A,* 103**,** 3890-5.

CHANG, B., HAWES, N. L., HURD, R. E., DAVISSON, M. T., NUSINOWITZ, S. & HECKENLIVELY, J. R. 2002. Retinal degeneration mutants in the mouse. *Vision Res,* 42**,** 517-25.

FEI, Y. & HUGHES, T. E. 2001. Transgenic expression of the jellyfish green fluorescent protein in the cone photoreceptors of the mouse. *Vis Neurosci,* 18**,** 615-23.

HAIDER, N. B., JACOBSON, S. G., CIDECIYAN, A. V., SWIDERSKI, R., STREB, L. M., SEARBY, C., BECK, G., HOCKEY, R., HANNA, D. B., GORMAN, S., DUHL, D., CARMI, R., BENNETT, J., WELEBER, R. G., FISHMAN, G. A., WRIGHT, A. F., STONE, E. M. & SHEFFIELD, V. C. 2000. Mutation of a nuclear receptor gene, NR2E3, causes enhanced S cone syndrome, a disorder of retinal cell fate. *Nat Genet,* 24**,** 127-31.

MEARS, A. J., KONDO, M., SWAIN, P. K., TAKADA, Y., BUSH, R. A., SAUNDERS, T. L., SIEVING, P. A. & SWAROOP, A. 2001. Nrl is required for rod photoreceptor development. *Nat Genet,* 29**,** 447-52.

PANG, J. J., ALEXANDER, J., LEI, B., DENG, W., ZHANG, K., LI, Q., CHANG, B. & HAUSWIRTH, W. W. 2010. Achromatopsia as a potential candidate for gene therapy. *Adv Exp Med Biol,* 664**,** 639-46.

SAMSON, M., EMERSON, M. M. & CEPKO, C. L. 2009. Robust marking of photoreceptor cells and pinealocytes with several reporters under control of the Crx gene. *Dev Dyn,* 238**,** 3218-25.

SANYAL, S., DE RUITER, A. & HAWKINS, R. K. 1980. Development and degeneration of retina in rds mutant mice: light microscopy. *J Comp Neurol,* 194**,** 193-207.

SIEGERT, S., SCHERF, B. G., DEL PUNTA, K., DIDKOVSKY, N., HEINTZ, N. & ROSKA, B. 2009. Genetic address book for retinal cell types. *Nat Neurosci,* 12**,** 1197-204.

WEST, E. L., GONZALEZ-CORDERO, A., HIPPERT, C., OSAKADA, F., MARTINEZ-BARBERA, J. P., PEARSON, R. A., SOWDEN, J. C., TAKAHASHI, M. & ALI, R. R. 2012. Defining the integration capacity of embryonic stem cell-derived photoreceptor precursors. *Stem Cells,* 30**,** 1424-35.
